# Extracellular-vesicle-mediated transfer of let-7b/7c promotes the proliferation of transition-state spermatogonia in neonatal mouse testis

**DOI:** 10.1016/j.stemcr.2025.102681

**Published:** 2025-10-23

**Authors:** Tingting Zheng, Kathleen Hoi Kei Choy, Sze Yan Chan, Min Zheng, Xiaotong Luo, Hao Chen, Ting Xie, Ellis Kin Lam Fok

**Affiliations:** 1 School of Biomedical Sciences, Faculty of Medicine, The Chinese University of Hong Kong, Hong Kong SAR, China; 2 Center for Tissue Regeneration and Engineering, JC STEM Laboratory for Regenerative Biology, Division of Life Science, School of Science, The Hong Kong University of Science and Technology, Hong Kong SAR, China; 3 Guangdong Institute of Gastroenterology, Biomedical Innovation Center, The Sixth Affiliated Hospital, Sun Yat-sen University, Guangzhou, China; 4 Guangzhou Medical University, Guangzhou, China; 5 Sichuan University-The Chinese University of Hong Kong Joint Laboratory for Reproductive Medicine, West China Second University Hospital, Chengdu, China

**Keywords:** spermatogonial stem cells, spermatogonia, extracellular vesicle, let-7 miRNA, intercellular communication

## Abstract

The self-renewal and differentiation of spermatogonial stem cells (SSCs) play essential roles in spermatogenesis. Extracellular vesicle (EV) is a universal strategy for intercellular communications in stem cell niches. However, the involvement of EVs in regulating SSCs remains largely unknown. This study revealed that testis EVs from postnatal day 7 (PND7) neonatal mouse testis guided spermatogonia into a transit-amplifying state with increased proliferation while retaining their differentiation potential. We profiled the repertoires of proteins and small RNAs by proteomic and small RNA transcriptomic analyses, respectively. We further showed that the EVs secreted by undifferentiated spermatogonia and the Sertoli cell lines, but not from more differentiated germ cell lines, conveyed let-7b/7c microRNA (miRNA) cargoes to spermatogonia, which mediated the effect of EVs on spermatogonial transit amplification. Together, this study has deciphered crucial let-7b/7c cargoes of EV-mediated communication within the spermatogonial niche, providing a new insight into the regulation of SSCs and spermatogenesis.

## Highlights

• Neonatal testis EVs drive spermatogonial transit amplification
• Neonatal and adult testis EVs have distinct small RNA and protein profiles
• Spermatogonia and Sertoli cell line EVs contribute to spermatogonial regulation
• The effect of testis and cell line EVs is mediated by the let-7b/7c miRNA cargoes

## Teaser

In this article, Fok and colleagues show that extracellular vesicles (EVs) from spermatogonia and Sertoli cells within the neonatal mouse testicular niche transport the let-7b/7c miRNAs, promoting spermatogonial transit amplification. These findings uncover an EV-based communication between spermatogonial subpopulation and Sertoli cells, offering new insights into the regulation of spermatogonial stem cell during the first wave of spermatogenesis.

## Introduction

Spermatogonial stem cells (SSCs) are the most primitive spermatogonia that support spermatogenesis through self-renewal and differentiation. Emerging evidence demonstrates that the delicate balance between self-renewal and differentiation of SSCs is strongly associated with the surrounding microenvironment, also termed a “niche.”

The SSC niche is supported by the germ cells and Sertoli cells within seminiferous tubules, which provide structural support, form the blood-testis barrier (BTB) (Oatley and Brinster, 2012), and secrete factors for the self-renewal (Ishii et al., 2012; Kubota et al., 2004; Yang et al., 2013) and differentiation of SSCs (Vernet et al., 2006). Somatic cells like peritubular myoid cells, Leydig cells, interstitial macrophages, and vasculature in the interstitial space also contribute to SSC maintenance (Oatley et al., 2009; Wang et al., 2015).

Extracellular vesicles (EVs) are known to act as messengers in intercellular communication via delivering cargoes, including DNAs, RNAs, proteins, and lipids from donor cells to the recipient cells (Welsh et al., 2024). Several studies have reported that EVs originating from specific cellular components of the SSC niche contribute to SSC regulation. For instance, Thy1^+^ EVs secreted by spermatogonia have been identified in the testicular microenvironment and to suppress the proliferation of SSCs (Lin et al., 2020). The EVs derived from primary Sertoli cells protect SSCs from oxidative stress and promote the proliferation and differentiation of SSCs via microRNA (miRNA) cargoes miR-486-5p and miR-30a-5p (Salek et al., 2021; Wang et al., 2023; Li et al., 2021). Nevertheless, the effects of EVs in regulating SSCs and the cargoes of EVs mediating these effects under a physiological condition remain largely unexplored. We hypothesize that EVs mediate intercellular communication within the SSC niche and that their cargos regulate SSC fate decisions.

This study comprehensively characterized the small RNA and protein cargoes of testis EVs at postnatal day 7, revealing their direct regulation of SSC proliferation and fate decision. Furthermore, we identified specific EV cargoes, let-7b/c miRNAs, as candidate regulators of SSC fate decisions that coordinate niche crosstalk between SSCs and supporting cells.

## Results

### Postnatal day 7 testis EVs promote spermatogonial proliferation

**Figure 1:**
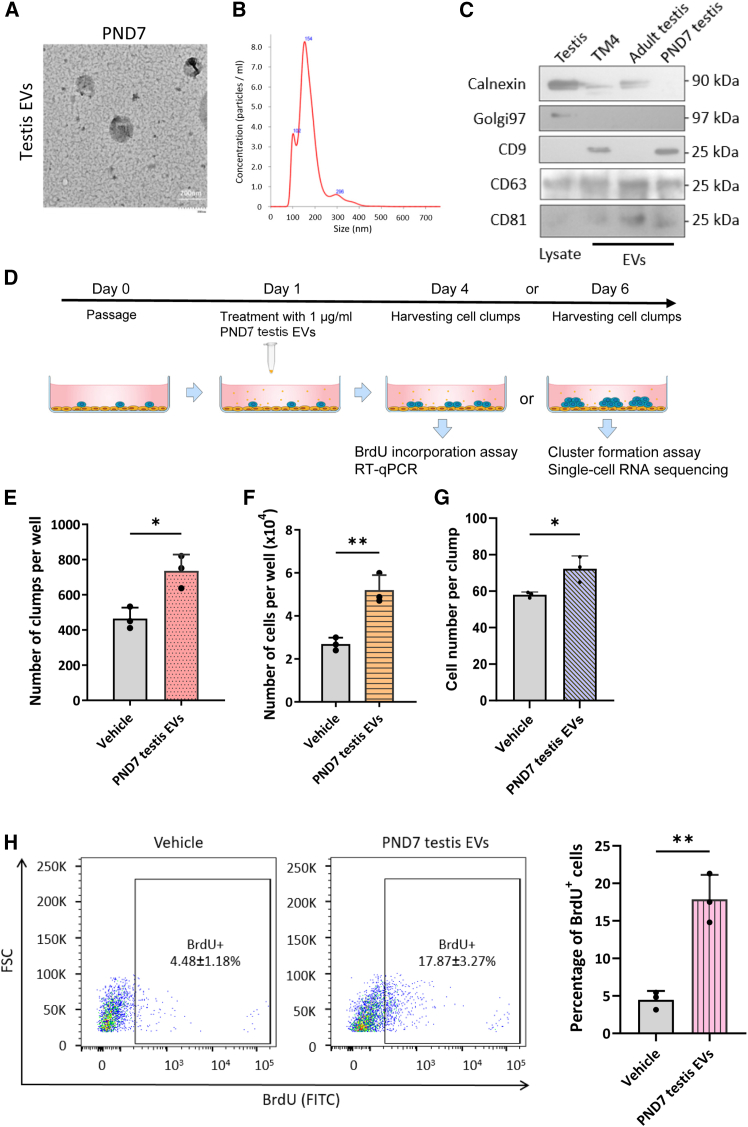
EVs in neonatal testis promote spermatogonial proliferation (A) Representative transmission electron microscopy image of PND7 mouse testis EVs by affinity columns. Scale bars, 200 nm. (B) Size distribution of PND7 testis EVs isolated as in (A) determined by nanoparticle tracking analysis. (C) Representative western blot analysis of EV markers CD9, CD63, and CD81 and endoplasmic reticulum markers calnexin and Golgi marker golgin 97 in the PND7 testis EVs and TM4-derived EVs. Protein lysate from mouse testes was used as control. (D) Schematic diagram showing the treatment protocol of PND7 testis EVs on primary spermatogonial culture. Counting of the numbers of SSC clumps (E) and total cell number of primary spermatogonia (F) after exposure to PND7 testis EVs (1 μg/mL) for 5 days. (G) The ratio of SSC clumps per 1,000 spermatogonial cells. (H) Representative flow cytometry analysis of BrdU uptake in primary spermatogonia exposed to PND7 testis EVs for 6 days (1 μg/mL) (see also Figure S2). *n* = 3 independent experiments.

We first asked if EVs isolated from the testis at postnatal day 7 (PND7), where most spermatogonial subpopulations are established, pose any effect on the SSCs. We isolated EVs from the whole decapsulated PND7 mouse testis at a stage where spermatogonia are the primary germ cells to minimize heterogeneity of EV source, using our recently developed protocol (Choy et al., 2022). This facilitates the characterization of EVs within the SSC niche. PND7 testis EVs exhibited hallmark cup-shaped morphologies (Figure 1A) of sizes 50–300 nm, with a major peak at around 150 nm (Figure 1B). These EVs expressed EV-specific markers CD9, CD81, and CD63, while lacking organelle markers calnexin and golgin 97 (Figure 1C). Small EVs represent the major population in the neonatal mouse testis, consistent with adult testis EVs (Choy et al., 2022; Yun et al., 2019).

To probe for the potential functions of PND7 testis EVs on SSCs, we added EVs to primary spermatogonial culture (Kanatsu-Shinohara et al., 2003), a heterogeneous culture consisting of different spermatogonial subpopulations including SSCs and committed progenitors (Figure 1D). Flow cytometry confirmed time-dependent uptake of PKH67-labeled EVs, with 9%, 19%, and 80% of primary spermatogonia positive for PKH67 signals after testis EV treatment for 3 h, 6 h, and 24 h, respectively, suggesting an efficient uptake after 24-h exposure (Figure S1). Exposure of primary spermatogonia to testis EVs for 3 days dose-dependently reduced the expression of undifferentiated spermatogonial markers, with significant effects at 1 μg/mL of testis EVs, a concentration used in subsequent experiments (Figure S1). A 5-day treatment did not alter the morphology but increased the number of SSC clumps (Figure 1E). Since the primary spermatogonial culture is heterogeneous with SSCs representing only 10% of the cells, we have further counted the cell number. Intriguingly, PND7 testis EV treatment significantly increased the total cell number (1.93-fold) and clump number (1.58-fold) of the heterogeneous spermatogonial culture (Figure 1F), suggesting that the PND7 testis EVs enhanced the spermatogonial proliferation or survival and more cell number present per clumps (Figure 1G). Consistent with the increase in proliferation, PND7 testis EV increased DNA replication of spermatogonia as revealed by an increased number of cells incorporating BrdU (Figure 1H), without inducing apoptosis or necrosis (Figure S2). Taken together, these results suggest that PND7 testis EVs promote spermatogonial proliferation or survival.

### Testis EVs guide spermatogonia to the transit-amplifying state

**Figure 2:**
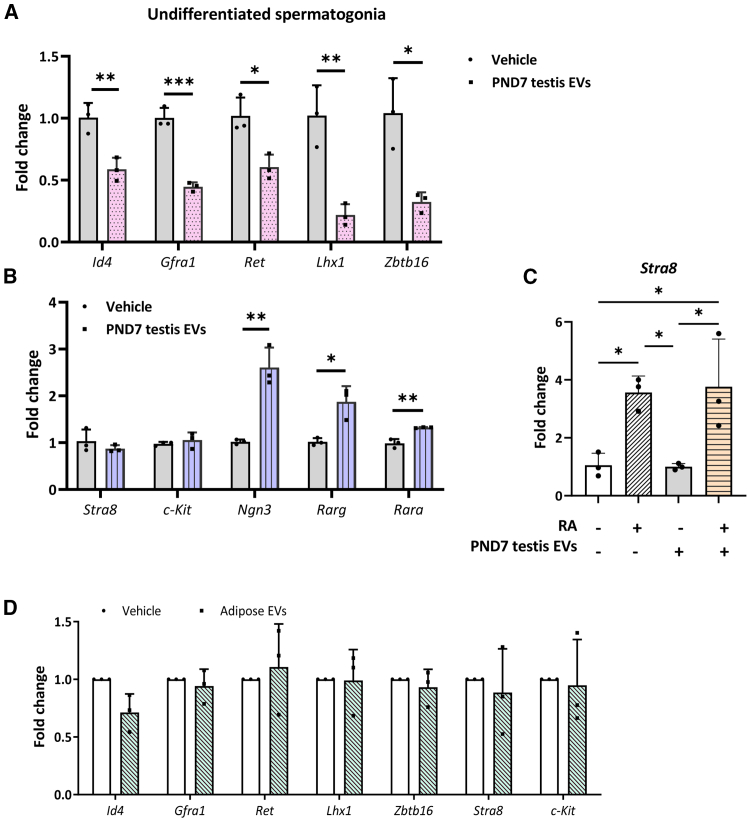
EVs from neonatal testis guide spermatogonia into a transit-amplifying state Real-time PCR results show that compared to the vehicle-treated group, treatment of PND7 testis EVs (1 μg/mL) (A) significantly decreased the expression of undifferentiated markers *Id4*, *Gfra1*, *Ret*, *Lhx1*, and *Zbtb16* in primary spermatogonia, (B) but had no effect on the expression of the differentiation markers *Stra8* and *c-Kit* (see also Figure S1). (B) The expression of *Ngn3*, *Rarg*, and *Rara* increases in primary spermatogonial culture after exposure to PND7 testis EVs. (C) PND7-testis-EV-treated spermatogonia showed a comparable induction of *Stra8* after RA treatment as compared to those without testis EV treatment. (D) Real-time PCR results showing the expression levels of undifferentiated and differentiating spermatogonia markers in primary spermatogonia after exposure to EVs isolated from adipose tissues. *n* = 3 independent experiments.

Next, we assessed the effect of testis EVs on the expression of self-renewal and differentiation markers of SSCs. We found that PND7 testis EVs inhibited the expression levels of SSC marker *Id4*; A_s_ spermatogonia marker *Gfra1*; and pan-undifferentiated spermatogonia markers *Zbtb16*, ret receptor tyrosine kinase (*Ret*), and LIM homeobox 1 (*Lhx1*) in the cultured spermatogonia (Figure 2A). Intriguingly, while differentiation markers *Stra8* and *c-Kit* were comparable, we observed a significant increase in the expression of *Ngn3*, retinoic acid (RA) receptors alpha (*Rara*) and gamma (*Rarg*), and markers of undifferentiated spermatogonia primed for differentiation (Figure 2B) (Gely-Pernot et al., 2015; Yoshida et al., 2004). Unexpectedly, despite the elevated *Rara* and *Rarg*, the PND7 testis EV-treated spermatogonia demonstrated a normal Stra8 response to RA (Figure 2C). Besides, adipose tissue EVs did not alter spermatogonial marker expression (Figure 2D), indicating that the observed effects of the PND7 testis EVs on primary spermatogonial culture are specific.

**Figure 3:**
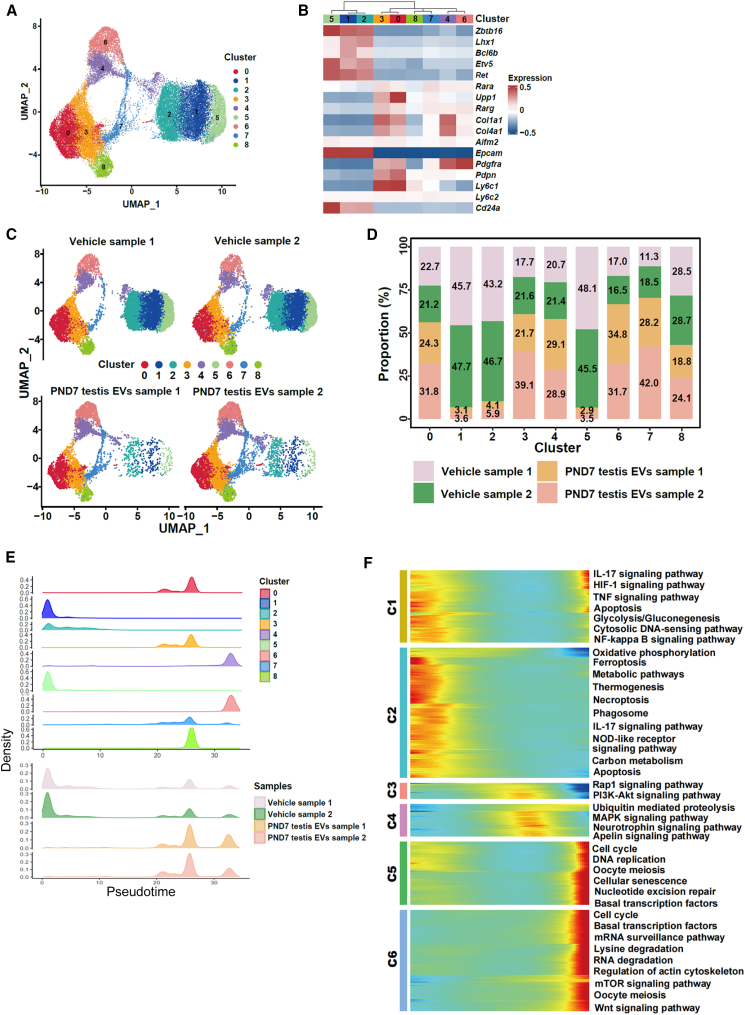
Single-cell analysis of primary spermatogonial culture upon neonatal testis EVs treatment (A) UMAP plots of a merged dataset containing vehicle and PND7-testis-EV-treated spermatogonia cultures, revealing nine clusters. (B) Heatmap displaying the expression of marker genes for stem cells, progenitors, and fibroblasts, collapsed from each cluster, with mRNA levels represented on a *Z* score scale. (C) UMAP plots of nine cell clusters within each sample. (D) Proportions of vehicle and PND7-testis-EV-treated spermatogonia across the nine clusters. (E) Pseudotime analysis of spermatogonia, illustrated using density maps to show the distribution of cells from different populations and samples in pseudotime. (F) Differentially expressed genes along pseudotime were hierarchically clustered into six profiles, with representative gene functions and pathways highlighted for each profile. *n* = 2 independent samples (see also Figure S3).

In corroboration with this, single-cell RNA sequencing analysis revealed that PND7 testis EV treatment significantly increased the progenitor population. Unsupervised clustering generated nine populations in primary spermatogonial culture (Figure 3A). Based on the known markers of various spermatogonial subpopulations (Figure 3B), nine clusters were classified into SSCs (clusters 1, 2, and 5) and progenitor spermatogonia (clusters 0, 3, 4, 6, and and 7). The UMAP analyses revealed that the PND7-testis-EV-treated spermatogonial cultures exhibited a marked shift from stem cells to progenitors compared to the control group (Figure 3C). The proportion of SSCs decreased from >40% in vehicle group (cluster 1: 46.7% ± 1.4%; cluster 2: 45.0% ± 2.5%; and cluster 5: 46.8% ± 1.8%) to <5% in the PND7-testis-EV-treated group (cluster 1: 3.4% ± 0.4%; cluster 2: 5.0% ± 1.3%; and cluster 5: 3.2% ± 0.4%), whereas a 1.4- and 2-fold increase in clusters 3 and 4 and 6 and 7, respectively (Figure 3D). Pseudotime analysis revealed six gene clusters that aligned well with spermatogonial development *in vivo*, including glial-cell-line-derived neurotrophic factor (GDNF) signaling (PI3K/Akt signaling) in SSC, the activation of cell cycle, and enhanced protein turnover (ubiquitin-mediated proteolysis) in progenitors (Figures 3E and 3F). Similarly, we observed PND7 testis EVs shift SSCs to progenitors (Figure 3E). This was supported by altered gene expression in key pathways: differential gene expression analysis within each cell cluster revealed significant alterations in key signaling pathways involved in SSC self-renewal (PI3K/Akt, mitogen-activated protein kinase [MAPK], and Ras signaling pathways) and progenitor differentiation (cell adhesion and extracellular matrix [ECM]-receptor interaction and Rap1 and HIF1 signaling pathways) (Figure S3), supporting the role of EVs in regulating the gene expression of recipient cells, ensued by the shift in cell status.

These results suggest that the PND7 testis EV treatment guides the spermatogonia into a transit-amplifying state.

### Repertoires of small RNAs and proteins in the PND7 testis EVs

Next, we investigated the cargoes of the PND7 testis EVs to identify potential candidates in mediating the effect on SSCs. Small RNA sequencing identified 2,131 small RNAs, representing the dominant RNA species in testis EVs (Choy et al., 2022), in PND7 testis EVs. Thirty-five percent of the clean reads were annotated as rRNA, tRNA, small nucleolar RNA (snoRNA), small nuclear RNA (snRNA), Rfam small non-coding RNA (sncRNA), and microRNAs (miRNAs), including precursor, mature and hairpin, whereas the remainder mapping to other genomic loci (Figure S4A). Notably, miRNAs represented 6.06% of the reads and were the predominant small RNA subtypes. Most of the 50 most abundant miRNAs in testis EVs have been demonstrated to mediate the maintenance and differentiation of stem cells (Table S1). For example, miR125b, miR-21, miR-378, and miR-652 serve as signals for maintaining the self-renewal of snail-induced stem cells, mesenchymal stem cells, and colon cancer stem cells, respectively (Deng et al., 2013; Yang et al., 2020; Yu et al., 2015); miR-34, miR-451, and let-7 families promote the differentiation of neural stem cells and cancer stem cells (Aranha et al., 2011; Bitarte et al., 2011; Büssing et al., 2008; Peng et al., 2017). We have subsequently selected 10 highly expressed miRNAs as the candidate for subsequent investigations.

To identify protein cargoes, our proteomic analysis identified 943 proteins in the PND7 testis EVs (Figure S4B; Table S2). These proteins represent the core proteome of the EVs in the neonatal mouse testis. Among these proteins, 88 were reported to be exosome protein markers (http://www.exocarta.org/) (Table S3), validating the successful EV isolation. Then, we mapped the proteins identified in the PND7 testis EVs to genes and conducted gene ontology (GO) analysis. The GO cellular component analysis showed that in line with the affinity column purification of membranous vesicles, proteins found in the PND7 testis EVs were enriched in the membrane and integral components of the membrane (Figure S4C). GO biological process analysis revealed proteins involved in cell adhesion, cell migration, and positive regulation of cell proliferation that were critical processes for the establishment of spermatogonia and Sertoli cells before the first wave of spermatogenesis (Figure S4D) (Manku and Culty, 2015). GO molecular function analysis identified protein binding as the major function of proteins in the PND7 testis EVs. Notably, genes encoding the proteins identified in the PND7 testis EVs were known to regulate the SSCs or other tissue stem cells (Figure S4E; Table S4). Together, our results have identified the small RNA and protein signatures of the PND7 testis EVs that may regulate the establishment and function of the SSC niche.

### EVs from spermatogonia and Sertoli cell lines mimic the effects of the PND7 testis EVs

**Figure 4:**
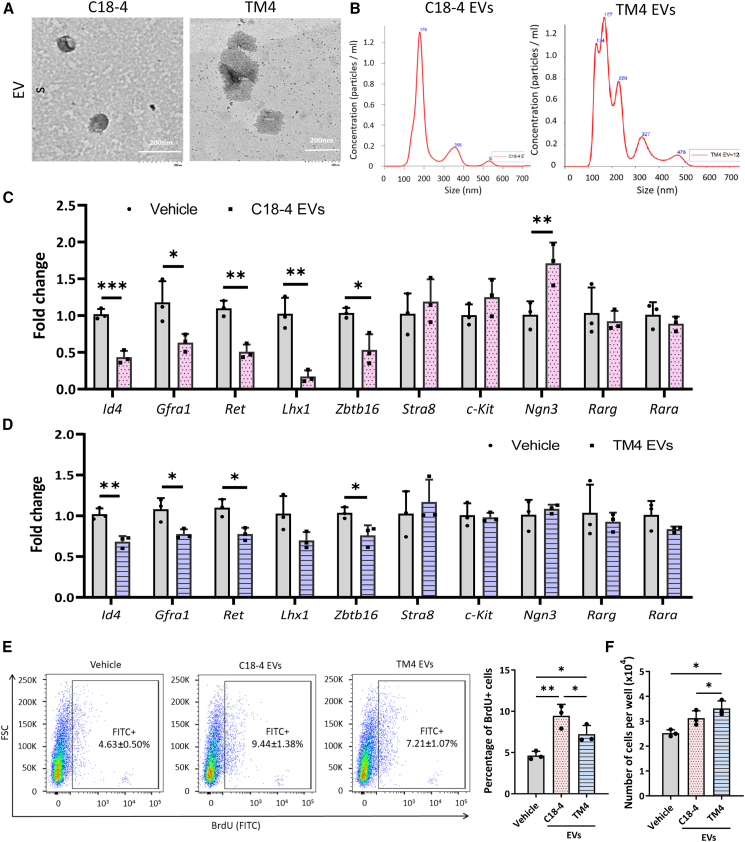
EVs from spermatogonia and Sertoli cells lines mimic the effects of PND7 testis EVs on primary spermatogonial culture (A) Representative transmission electron microscopy image of EVs isolated from C18-4 and TM4 cell lines by ultracentrifugation. Scale bars, 200 nm. (B) Size distribution of cell-line-derived EVs isolated as in (A) determined by nanoparticle tracking analysis. Real-time PCR results showing the expression of SSC marker *Id4*; A_s_ spermatogonia marker *Gfra1*; pan-undifferentiated spermatogonia markers *Zbtb16*, *Ret*, and *Lhx1*; spermatogonial differentiation markers *Stra8* and *c-Kit*; and progenitor markers *Ngn3*, *Rara*, and *Rarg* in primary spermatogonia after treatment with EVs isolated from C18-4 (C) and TM4 (D) cell lines (1 μg/mL). (E) Representative flow cytometry analysis of BrdU uptake in primary spermatogonia exposed to C18-4 and TM4 EVs (1 μg/ml), and (F) total cell number of primary spermatogonia exposed to C18-4 and TM4 EVs. *n* = 3 independent experiments (see also Figure S5).

In the PND7 testis, the undifferentiated and differentiating spermatogonia undergo the first wave of spermatogenesis, whereas Sertoli cells undergo proliferation. This event is crucial for the Sertoli-germ cell contact and facilitates the formation of the SSC niche during the first round of spermatogenesis. To decipher the donor cells that contribute to the effect of the PND7 testis EVs, we have isolated the EVs from immortalized cell line models: C18-4 undifferentiated spermatogonial cell line, the GC1-spg differentiating spermatogonia cell line, and TM4 Sertoli cell line cultures. Although these immortalized cell lines are incomplete representations of given cell types in SSC niche, this approach overcomes the technical difficulties of obtaining sufficient pure primary cells for EV collections. Since the primary spermatocyte first appears at PND14, GC2-spd spermatocyte cell line was used as a control. The EVs from the four cell lines showed cup-shaped morphology with a size of 100–250 nm and a peak of size distribution at around 150 nm (Figures 4A, 4B, and S5).

Next, we test the biological activities of the cell-line-derived EVs by adding cell-line-derived EVs to the primary spermatogonial culture. EVs isolated from C18-4 undifferentiated spermatogonial cell line (C18-4 EVs) and the TM4 Sertoli cell line (TM4 EVs) significantly inhibited the expression of undifferentiated spermatogonia markers, including *Id4*, *Gfra1*, *Zbtb16*, *Ret*, and *Lhx1*, while the markers for differentiating spermatogonia, *Stra8* and *c-Kit*, remained unchanged (Figures 4C and 4D). These results highly resemble those observed for the PND7 testis EV treatment of spermatogonial culture. Interestingly, the expression levels of *Ngn3* increased in the spermatogonia exposed to the C18-4 EVs but not the TM4 EVs. Moreover, the levels of *Rarg* and *Rara*, which both were upregulated by the PND7 testis EVs, were not altered by the C18-4 or TM4 EVs (Figures 4C and 4D). The EVs isolated from GC1-spg (GC1-spg EVs) and GC2-spd cell lines (GC2-spd EVs) did not pose any significant effect on the expression profiles of spermatogonia markers (Figure S5). In line with the expression of markers, BrdU assay demonstrated that C18-4 and TM4 EVs significantly increased the percentage of BrdU-incorporating cells and cell numbers in the primary spermatogonial culture (Figures 4E and 4F). These results suggest that the EVs originating from spermatogonia and Sertoli cell lines mimic the effects of the PND7 testis EVs on spermatogonial proliferation and differentiation.

### Involvement of let-7b/c miRNA cargoes in mediating the effect of the PND7 testis EVs

Our previous experiments showed that the PND7 testis EVs promoted spermatogonial proliferation and engaged spermatogonia at the transit-amplifying state. Since the PND7 testis EVs carried miRNA cargoes, some of which had been reported to regulate the maintenance of stem cells, we postulated that these miRNA cargoes were transferred to the spermatogonia to regulate their proliferation and fate decision.

**Figure 5:**
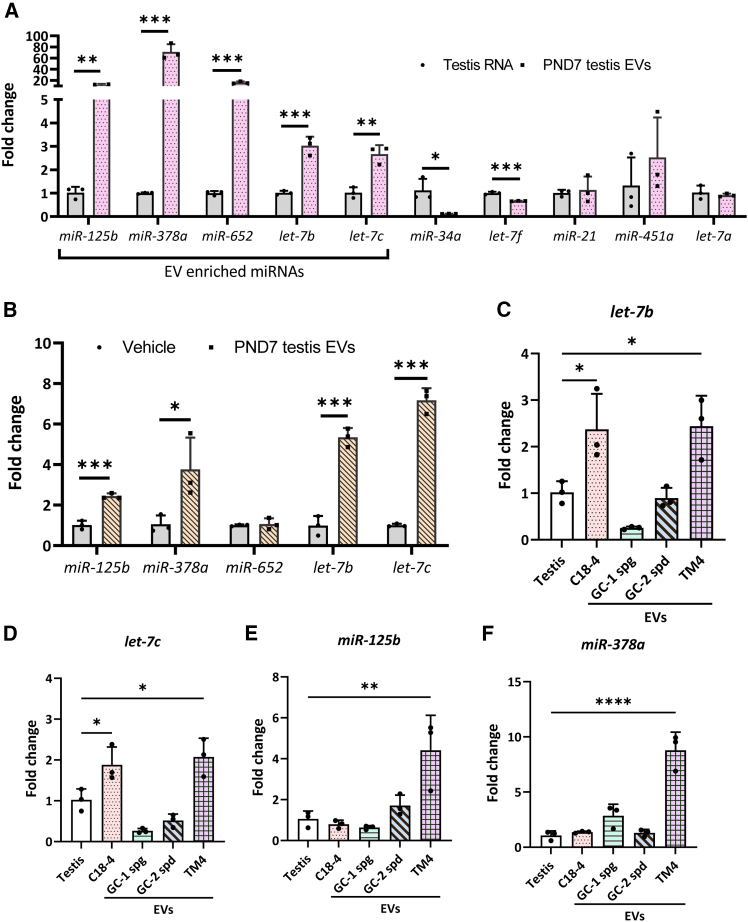
EVs from neonatal testis convey EV-enriched miRNAs to primary spermatogonia Real-time PCR results showing the expression of indicated miRNAs in (A) PND7 testis EVs and (C–F) EVs isolated from C18-4, TM4, GC1-spg, and GC2-spd cell lines. Total testis RNA (testis RNA or testis) was used as a control. (B) The expression of indicated miRNAs in primary spermatogonial treated with PND7 testis EVs (1 μg/mL) or same volume of PBS in the medium (vehicle). Data were normalized to snoU6 RNA levels.

To test this hypothesis, we validated the candidate miRNA expression of the PND7 testis EVs (Table S1). MiR-125b, miR-378a, miR-652, let-7b, and let-7c were significantly enriched in the PND7 testis EVs. In contrast, miR-34a and let-7f were substantially lower, while miR-21, miR-451a, and let-7a were comparable to total testis RNA (Figure 5A). To examine if the miRNA cargoes carried by the PND7 testis EVs were transferred to spermatogonia, we profiled the miRNAs in primary spermatogonia after the treatment with the PND7 testis EVs. Indeed, the expression levels of miR-125b, miR-378, let-7b, and let-7c were significantly increased in the EV-treated spermatogonia (Figure 5B), suggesting that these miRNAs were transferred to spermatogonia. In addition, let-7b and let-7c were highly enriched in the C18-4 EVs and TM4 EVs, representing a 2.4-fold and 2-fold enrichment compared to that of the total RNA extracted from mouse testes (Figures 5C and 5D). Consistent with the lack of response in the EVs isolated from the GC1-spg and GC2-spd lines, let-7b and let-7c were significantly lower in the GC1-spg EVs and GC2-spd EVs (Figures 5C and 5D).

To further examine the involvement of let-7b/c in mediating the effect of testis EVs, we attempted to establish loss-of-function models targeting let-7b and let-7c. Due to the highly conserved and extremely short seed sequence of the let-7 family, we used single guide RNAs (sgRNAs) targeting the Dicer processing sites of let-7b and let-7c precursors instead of conventional sgRNA on seed sequence or inhibitors/antagomirs. Using lentiviral CRISPR/Cas9, we mutated the precursors in C18-4 and TM4 cell lines to perturb let-7b and let-7c biogenesis. This reduced mature miRNA levels to 0.4- to 0.6-fold (Figure S6), an outcome mimicking the knockdown condition. Thus, we referred to these mutant cell lines as let-7b or let-7c knockdown (let-7b KD or let-7c KD).

**Figure 6:**
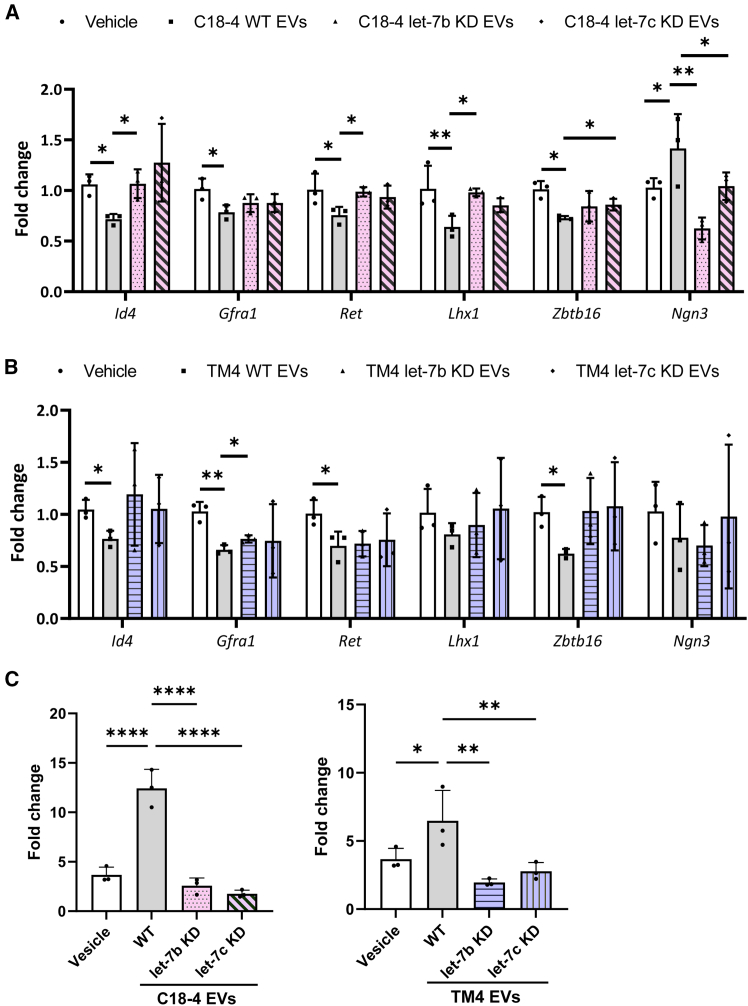
Knockdown of let-7b/7c in donor cells perturbs the effects of EVs on primary spermatogonial culture Real-time PCR results showing the expression of *Id4*, *Gfra1*, *Zbtb16*, *Ret*, *Lhx1*, and *Ngn3* in WT or KD C18-4 lines (A) or TM4 lines (B). (C) Percentages of BrdU uptake in primary spermatogonia exposed to WT or KD C18-4 lines or TM4 lines. *n* = 3 independent experiments (see also Figure S6).

We found that compared to the vehicle-treated group, the EVs from wild-type (WT) C18-4 and TM4 cell lines could significantly inhibit the expression of undifferentiated markers *Id4*, *Gfra1*, *Zbtb16*, and *Ret*, and C18-4 EVs also induce the expression of *Ngn3* in primary spermatogonia. However, the effects of EVs were partially inhibited in let-7b or let-7c KD C18-4 and TM4 cell lines (Figures 6A and 6B). These results suggest that let-7b and let-7c are the potential cargoes of the PND7 testis EVs being conveyed to spermatogonia and mediating the effect of EVs on spermatogonial proliferation and differentiation.

## Discussion

In the present study, we have shown that PND7 testis EVs obtained from a physiological source after the establishment of spermatogonial populations in the niche significantly promote spermatogonial proliferation and prime spermatogonia to a transit-amplifying state, strongly suggesting a role in the intercellular communication of SSCs and other spermatogonial subpopulation after the establishment of the niche. However, it should be noted that the testis EVs analyzed in this study represent a heterogeneous mixture derived from multiple cell types present in PND7 testis. Therefore, the observed effects on SSCs represent the combinatorial effects of EVs secreted by all cell types in the testis, regardless of the spatial effect of these cells within and outside the niche. While we traced the origin of biologically active EVs from spermatogonia and Sertoli cells using established cell lines, isolating EVs from primary testicular cells remains technically challenging. Therefore, the results should be interpreted with caution that the C18-4, GC1-spg, and TM4 cell lines, while useful proxies, may not fully replicate the native functions of spermatogonial subpopulations or Sertoli cells in the SSC niche.

The intracytoplasmic bridges interconnect mitotic sister spermatogonia. Previous studies have reported the competition among GFRα1-positive spermatogonia for mitogens that determine the homeostasis of SSC (Kitadate et al., 2019). The Thy1^+^ EVs isolated from Thy1^+^ spermatogonia inhibit spermatogonia proliferation and decrease the number of SSC clumps, whereas spermatogonia-derived Thy1^−^ EVs have no effect, and these spermatogonia-derived EVs do not alter the apoptosis of primary spermatogonia (Lin et al., 2020). Consistent with these findings, our results showed that the PND7 testis EVs posed negligible effects on the apoptosis of spermatogonia. However, in contrast to the Thy1^+^ EVs, testis EVs increased the proliferation of primary spermatogonia. It is worth noting that the testis EVs are produced by various cell types in the testis. While our study could not exclude the involvement of Thy1^+^ EVs, our results together with the previous study suggested a combinatorial effect of EVs from spermatogonia and other somatic cells in the SSC niche. More importantly, the present study suggested that both neighboring spermatogonia and Sertoli cells are the pivotal components in the SSC niche via EV-mediated intercellular communication.

In the SSC niche, different spermatogonial subpopulations are exposed to potential intercellular communication from the neighboring undifferentiated and differentiated spermatogonia, Sertoli cells, Leydig cells, and other somatic cells such as endothelial cells and macrophages. In this study, the EVs from C18-4 undifferentiated spermatogonia and TM4 Sertoli cell lines could partially recapitulate the effects of testis EVs on spermatogonial cultures, while those secreted from GC1-spg premeiotic differentiated spermatogonia are dispensable, implying that the EV-mediated intercellular communication is more restricted to the primitive spermatogonial colonies and their microenvironment. It should be noted that the SSC niche also involves other untested somatic cells, e.g., Leydig cells, macrophages, endothelial cells, and peritubular myoid cells. To date, the contribution of endothelial cells and peritubular myoid cells to testis EVs has not been reported. Interestingly, both Leydig cells and macrophages have been postulated to contribute to the testis EVs (Choy et al., 2022), which may contribute to the biological effects of testis EVs, for example, the upregulation of *Rara* and *Rarg* (Figure 2B), that are not observed from the C18-4 EVs and the TM4 EVs. Further studies are required to investigate the role of EVs from these other niche cells on SSCs.

An important finding of our study is the identification of let-7b and let-7c as the functional miRNA cargo candidates of testis EVs in regulating spermatogonial proliferation and transit amplification. Let-7b and let-7c KD significantly suppressed the effects of the C18-4 or TM4-derived EVs on spermatogonia, despite incomplete knockdown. We postulate that let-7b and let-7c hairpin precursors in the EVs produced by mutant lines could still be functional upon uptake. Nonetheless, our results showed that let-7b/7c were two miRNA cargoes that mediated the effect of testis EVs.

EVs are promising tools for genetic engineering and therapy due to their role in intercellular communication and cargo delivery. Our study shows that efficiently taken up by 80% of spermatogonia after the 24-h treatment, testis EVs offer a more effective vehicle for genetic engineering than the traditional transfection or transduction techniques. Testis EVs also induce SSC and progenitors proliferation, aiding the *in vitro* propagation of these spermatogonial populations. In addition, testis EVs may be used for the treatment of non-obstructive azoospermia, which imputes the defects of spermatogenesis by targeting spermatogonia.

Taken together, our study has revealed, for the first time, that neonatal mouse testis EVs promote spermatogonial proliferation and retain the differentiation potential of primary spermatogonial cultures, providing solid evidence for the participation of testis EVs in the intercellular communication within the SSC niche. EVs from Sertoli and undifferentiated spermatogonia cell lines partially recapitulate the effects of testis EVs via the miRNA cargoes let-7b/7c, suggesting that the EV-mediated intercellular communication involves the neighboring spermatogonia and their microenvironment. Our study has shed light on the EV-mediated regulation of the SSC niche and fate decision.

## Methods

### Animals

PND7 and 8-week-old male C57BL/6 mice were purchased from the laboratory animal service center (LASEC) of the Chinese University of Hong Kong. All procedures for the animal experiments were approved by the Animal Research Ethics Committee of the Chinese University of Hong Kong (20/081/ECS-5-C).

### Isolation and culture of primary spermatogonia

Primary spermatogonial culture was established from three PND6 male mice as previously described (Fok et al., 2017; Kanatsu-Shinohara et al., 2003; Nagano et al., 2003). Briefly, the decapsulated testes were dissociated with scissors and placed in solution I (1 mg/mL Collagenase Type IV, Sigma) for 10 min at 37°C. Tissues and cells were collected by brief centrifuge at 300×g for 15 s, followed by incubation in solution II (0.25% Trypsin, Gibco; 0.5 mL 5 mg/mL DNase, Thermo Fisher) for 10 min at 37°C, then stopped by the addition of 10% fetal bovine serum (FBS). The cell suspension was filtered through a 40-μm strainer, and cells were collected by centrifugation at 300×g for 5 min.

Spermatogonia were isolated by magnetic activated cell sorting (MACS) against Thy1; 1 × 10^7^ cells resuspended in 200 μL of DMEM medium supplemented with 2% FBS were incubated with 20 μL of biotinylated Thy1.2 CD90.2 primary antibody (BD IMag, 551518) on ice for 15 min on a slow-rocking platform. Cells were washed with PBS and resuspended with 500 μL of DMEM medium supplemented with 2% FBS before being loaded into the pre-calibrated MS column (Miltenyi Biotec, 130-042-201) to capture Thy1^+^ cells. Columns were washed twice with 500 μL of DMEM medium supplemented with 2% FBS, and the Thy1^+^ cells were eluted with 1 mL of DMEM medium supplemented with 2% FBS. Then, the Thy1^+^ cells were cultured on the STO feeder cells, as previously described (Kubota et al., 2004), in culture medium (StemPro 34 medium with supplement [Invitrogen]; 0.1 mg/mL Fe-saturated transferrin [Sigma]; 5 mg/mL bovine serum albumin [Sigma]; 6 mg/mL D-(L)-glucose [Sigma]; 30 nM sodium selenite [Sigma]; 5 × l0^−5^M p-mercaptoethanol [Sigma]; 60 mM putrescine [Sigma]; 25 μg/mL insulin [Sigma]; 30 μg/mL pyruvic acid [Sigma]; 1 μL/mL DL-lactic acid [Sigma]; minimal essential medium [MEM] vitamin solution [Invitrogen]; MEM nonessential amino acid solution [Invitrogen]; 10^−4^ M ascorbic acid [Sigma]; 10 μg/mL d-biotin [USB corp]; 30 ng/mL β-estradiol [Sigma]; 60 ng/mL progesterone [Sigma]; 2 mM L-glutamine [Life Technologies]; 1% fetal bovine serum [Gibco]; 10 ng/mL human basic fibroblast growth factor [hFGF] [Sigma]; 1% P/S and 20 ng/mL recombinant human glial-cell-line-derived neurotrophic factor (GDNF) [R&D Systems]) and maintained at 37°C. The culture medium was changed every 2–3 days and the stem cells proliferated to form tight clumps that were loosely attached to the STO feeders after 6–7 days in culture.

### Cell lines culture

The cell lines used in this study were the C18-4 mouse SSC line, GC1-spg mouse differentiating spermatogonia cell line, GC-2 spd mouse spermatocyte cell line, and TM4 mouse Sertoli cell line. C18-4 cells were cultured in DMEM medium supplemented with 10% FBS, 1% L-glutamine, 1 mM sodium pyruvate, and 1× nonessential amino acids at 35°C. GC1-spg cells and GC-2 spd were maintained in DMEM medium containing 10% FBS and 1% penicillin and streptomycin at 37°C. TM4 cells were cultured in DMEM/F12 medium containing 10% FBS and 1% penicillin streptomycin at 37°C.

### Isolation of EVs from the testis tissue, testicular cell lines, and adipose tissue

The PND7 stage was strategically selected as spermatogonial subpopulations were established and represent the dominant germ cells in the testis at this developmental stage, simplifying the EV source. EVs were isolated from 10 PND7 C57/BL6 male mice as described (Choy et al., 2022). Testes were decapsulated and release tubules using forceps and then digested by 1 mL Accumax (Thermo Fisher) per 20 mg tissue at room temperature for 1 h. Tissues were gently pipetted up and down 20 times to maximize the cell yield followed by filtering through a 40-μM strainer. The digestion was stopped by the addition of one volume of PBS, and the cells were pelleted by centrifugation at 300×g for 10 min. EVs were isolated from the supernatant using exoEasy Maxi Kit (Qiagen, 76064) according to the manufacturer’s instructions, then concentrated by ultracentrifugation (Hitachi CS-150GXII Micro UITRAcentrifuge) at 100,000×g for 90 min at 4°C. The EV pellet was resuspended in 20 μL PBS and stored at −80°C for further analysis.

EVs were isolated from cell lines grown to 95% confluency in ten 100-mm cell culture dishes (Genetimes, 20100) with 10 mL conditioned culture medium (CCM) supplementary with exosome-depleted FBS (exosomes were removed by ultracentrifugation at 150,000×g for 18 h at 4°C) in each dish and incubated for 48 h. The EV-containing CCM was collected, and the EVs were isolated by differential ultracentrifugation method. Briefly, CCM was sequentially centrifuged: 300×g 10 min (remove cells); 3,000×g 20 min (remove debris); and 10,000×g 30 min (remove large vesicles, Beckman Avanti J-E Centrifuge) at 4°C, followed by ultracentrifuge (Beckman Optima XPN-100 Ultra-High Speed Refrigerated Centrifuge) at 100,000×g 4°C for 90 min to pellet the small vesicles. The pellets were washed by PBS with ultracentrifuge (Hitachi CS150FNX Ultra-Microcentrifuge) at 100,000×g 4°C for 90 min. The EV pellet was resuspended in 20 μL PBS and stored at −80°C for further analysis.

Adipose-derived EVs were isolated from mouse visceral adipose tissue, as previously reported (Wei et al., 2020). Tissue samples were washed in PBS, minced into ∼2 mm^3^ fragments, and incubated in serum-free medium at 37°C for 24 h. The resulting conditioned medium was collected, and EVs were isolated using differential ultracentrifugation, as previously described.

### Transmission electron microscope

EV morphology was observed using TEM. Briefly, EVs were resuspended in 20 μL PBS and fixed with 2% paraformaldehyde until use; 10 μL EVs were added onto the formvar grid (200 mesh) for 30–60 min, and excess fluid was removed with filter paper. EVs were fixed with 1% glutaraldehyde for 10 min, followed by negative staining with 2% uranyl acetate for 2 min, and three images were captured at different fields using a Hitachi H-7700 transmission electron microscope (TEM).

### Nanoparticle tracking analysis

EV concentration was measured by NanoSight LM14C (Malvern). Briefly, EV samples were diluted with filtered PBS to ∼10^7^–10^9^ particles/mL, which allows the viewing of approximately 20–100 particles in each field. Three 30-s videos were captured at three different fields. The concentration was calculated by the NanoSight software using the Stokes-Einstein equation.

### Western blot

Protein from EVs, cells, and tissues was extracted by RIPA lysis buffer with protease and phosphatase inhibitor. A total of 40 μg of protein was denatured followed by loading on 10% SDS-PAGE gel for electrophoresis and transferring onto the polyvinylidene fluoride (PVDF) membranes (Sigma). The membrane was then blocked with 5% non-fat milk in Tris-buffered saline with Tween 20 (TBST) at room temperature for 1 h and immunoblotted with primary antibodies CD 63 (Santa Cruz; sc-365604, 1:1,000), CD 81 (Santa Cruz; sc-166029, 1:1,000), CD9 (Santa Cruz; sc-13118, 1:1,000), calnexin (Immunoway; YT0613, 1:1,000), golgin 97 (Santa Cruz; sc-59820, 1:1,000), and β-tubulin (Cell Signaling; 2146, 1:2,000) overnight at 4°C. After incubation with HRP-conjugated secondary antibody, immunoreactions were detected by Amersham ECL Advance Western Blotting Detection Kit (GE Healthcare, RPN2135) and Super X-film (Fuji Medical).

### Uptake of PKH67-labeled testis EVs by primary spermatogonia

Purified EVs were labeled using the PKH67 Green Fluorescent Cell Linker Mini Kit (Sigma-Aldrich) according to the manufacturer’s instructions. Briefly, 100 μL of EVs resuspended in PBS was mixed with 1 mL Diluent C and 6 μL PKH67 dye, followed by 5-min incubation. Excess dye was quenched by adding 2 mL of 0.5% BSA/PBS, and 20 mL PBS was added to prevent vesicle aggregation during ultracentrifugation. Labeled EVs were washed via ultracentrifugation and resuspended in 50 μL PBS. Primary spermatogonia were co-cultured with labeled EVs for 3, 6, and 24 h, and EV uptake was analyzed by flow cytometry.

### EV treatments

One microgram of EVs or an equal volume of PBS was added into 1 mL of fresh complete culture medium supplemented with 20 ng GDNF and 10 ng hFGF. After mixing, the mixture was filtered with 0.22 μm Millex-GP filters (Millipore Sigma, SLGP033RB). On day 1 post-passaging, the filtered culture medium was added to the primary spermatogonial culture, and the cells were incubated at 37°C in an incubator with 5% CO_2_ for 3 days.

### BrdU incorporation assay

The culture medium was removed and replaced with fresh medium containing 10 μM BrdU for 12 h at 37°C. After incubation, spermatogonial clumps were collected by flushing from the feeder cells as described (Fok et al., 2017). The clumps were collected and trypsinized into a single-cell suspension, followed by fixing with dropwisely adding 5 mL ice-cold 70% ethanol and incubated at −20°C for 2h and permeating with 2 M HCl/0.5% Triton X-100 at room temperature for 30 min. After washing twice with 1% BSA in PBS, cells were incubated with the anti-BrdU antibody (Abcam, ab6326, 1:100) and anti-DDX4/MVH antibody (Abcam, ab13840, 1:100) at room temperature for 1 h. Then, the samples were stained with Alexa Fluor 488 anti-rat immunoglobulin G IgG) (H + L) (Invitrogen, A21208, 1:1,000) and Alex Fluor 647 anti-rabbit IgG (H + L) (Invitrogen, A31573, 1:1,000) at room temperature for 1 h and finally resuspended in the solution of 1% BSA in PBS to 10^6^–10^7^ cells/mL and analyzed by flow cytometry (BD LSRFortessa Cell Analyzer). The results were analyzed by FlowJo v.10.

### Clump formation assay

Primary cultured spermatogonia were seeded at 1 × 10^4^ cells/cm^2^ in 48-well culture dishes (0.96 cm^2^, ∼10^4^ cells/well). The culture was treated with 1 μg/mL testis EVs for 6–7 days. Clumps were counted manually on the 6th day under an inverted fluorescence microscope. The clump is defined as a group of at least six cells with globular three-dimensional structures attached to the feeder cells (Yeh et al., 2007).

### Reverse transcription and quantitative real-time PCR

Prior to RNA extraction, EVs samples were treated with 0.05 μg/μL Proteinase K (Qiagen, 19131) at 37°C for 10 min, 5 mM PMSF (Sigma-Aldrich, PND7627) at room temperature for 10 min, and 0.5 μg/μL Rnase A (Thermo Scientific, EN0531) at 37°C for 20 min to remove proteins and RNAs outside the EV membranes.

For miRNA assays, miRNAs and RNAs <200 nucleotides were extracted using miRNeasy Mini Kit (Qiagen, 217004) according to the manufacturer’s instructions. A total amount of 20 ng small RNA was used for reverse transcription using TaqMan MicroRNA Reverse Transcription Kit (Applied Biosystems, 4366597). Then, 1 μl of cDNA was used for a real-time PCR using TaqMan Universal PCR Master Mix (Applied Biosystems, 4364340) on the ABI QuantStudio 7 Pro Real-time PCR System.

For gene expression analysis, cells or tissues were lysed with 700 μL QIAzol lysis reagent, and RNAs were separated by chloroform and precipitated by isopropanol to extract total RNAs. A total amount of 300 ng mRNA was used for reverse transcription reaction with High-Capacity cDNA Reverse Transcription Kit (Thermo Fisher, 4368814). One microliter of cDNA was used for a real-time PCR using SYBR Green Premix Ex Taq (Tli RNase H Plus) (Takara, RR420D) on the ABI QuantStudio 7 Pro Real-time PCR System.

The primers and probes used are listed in Tables S5 and S6. The data were calculated by the 2^(−ΔΔCT)^ method.

### Generation of let-7b and let-7c mutant cell lines

The let-7b and le-7c mutant cell lines were generated by the CRISPR/Cas9 system. The sequences of miRNA precursor were downloaded from miRBase v.16.0 (http://www.mirbase.org/), and sgRNAs were designed by the online tool CRISPR DESIGN (http://crispr.mit.edu/). It is not available to target the seed sequence because of the high sequence homology and short seed sequence of the let-7 family. Thus, we target the Dicer processing sites of precursor miRNAs to alter the biogenesis of let-7b and let-7c. Oligoes were purchased from Integrated DNA Technologies, and their sequences are shown in Table S6. The CRISPR/Cas9 and sgRNAs were delivered to target cells by lentiviral transduction.

### RA treatment

Retinoic acid (Sigma-Aldrich, R2625) stock solution (100 mM) was prepared by dissolving 50 mg RA powder in DMSO. A single-use aliquot of RA was prepared and stored at −80°C. Primary spermatogonia were exposed to 100 nM RA 3 days after testis EV treatment. Primary spermatogonia were collected for RT-qPCR studies at indicated time points (1–24 h).

### Small RNA sequencing

RNA was extracted from EVs after treatment with proteinase K, PMSF, and RNase A using miRNeasy Mini Kit as described above. Small-RNA libraries were prepared, and the PCR products were sequenced using BGISEQ-500 technology. Small RNA sequencing was performed by BGI (Shenzhen, China). After eliminating the low-quality reads, clean reads were mapped to reference genome and to other sRNA databases using Bowtie2 and cmsearch. Classification of sRNA follows the priority rule—MiRbase> pirnabank> snoRNA(human/plant)> Rfam> other sRNA—to ensure a unique map of each entry. Novel miRNAs and piRNAs were predicted using miRDeep2 and Piano, respectively.

### Mass spectrometer proteomic analysis

Testis EV proteins were extracted with RIPA buffer, and protein concentrations were detected by bicinchoninic acid (BCA) assay. Six micrograms of proteins in each sample were used to perform Bruker timsTOF Pro mass spectrometer proteomics analyses by Biosciences Central Research Facility of the Hong Kong University of Science and Technology. GO enrichment and KEGG pathway enrichment were analyzed.

### Single-cell RNA sequencing

Cell clumps were digested into single cells and resuspended in the 0.04% BSA in PBS. The cell concentration should be 1,000 cells/μL and with more than 85% viability, detected by Countess II Automated Cell Counter. Then, the single-cell library was prepared according to the 10× Genomics Single Cell protocol.

The input dataset was aligned with the mouse genome (mm10, GENCODE vM23/Ensembl 98) as appropriate and used the Cell Ranger v.7.0.1 Single-Cell Software Suite from 10× Genomics to estimate partitions containing cells and their unique molecular identifiers (UMIs). Cell filtering, data normalization, and unsupervised clustering were carried out using the Seurat R package. Ultimately, we identified 23,904 genes and detected 29,853 cells across four samples. We performed principal-component analysis (PCA) on the corrected expression matrix, focusing on highly variable genes (HVGs) identified by the “FindVariableFeatures” function. Afterward, we classify different cell types with the “FindClusters” function.

To comprehensively annotate the specific types of cell clusters, we examined the expression of the following marker genes and performed hierarchical clustering analysis using the R package pheatmap: *Zbtb16*, *Lhx1*, *Bcl6b*, *Etv5*, and *Ret* for SCC cells; *Rara*, *Upp1*, and *Rarg* for progenitor cells; and *Col1a1*, *Col4a1*, *Aifm2*, *Epcam*, *Pdgfra*, *Pdpn*, *Ly6c1*, *Ly6c2*, and *Cd24a* for fibroblasts. In addition, we investigated the lineage relationship among three myofibroblast subtypes using Monocle2.

To identify differentially expressed genes between vehicle samples and PND7 testis EV samples for each cell subtype, we used the “FindMarkers” function with default parameters. A gene was considered differentially expressed if it had an adjusted *p* value <0.05 and an absolute log2 (fold change) > 1. We used the R package clusterProfiler to conduct KEGG pathway enrichment analysis on the significantly differentially expressed genes.

### Statistical analysis

All experiments were repeated independently at least three times (*n* = 3 biological replicates). For RT-qPCR experiments, each biological replicate consisted of *n* = 3 technical replicate wells per condition. For small RNA sequencing analysis, due to the limited yield of the EV-treated primary spermatogonia and PND7 testis EVs, two sets of independent cell cultures per treatment group were used. Statistical analyses were performed using GraphPad Prism 9.0 software. Data are presented as mean ± SD by t test for two groups and one-way ANOVA for three groups of comparation; *n* = 3 independent experiments. Results were presented by mean ± SD, ^∗^*p* < 0.05, ^∗∗^*p* < 0.01, ^∗∗∗^*p* < 0.001, and ^∗∗∗∗^*p* < 0.0001, by Student’s t test for two groups and one-way ANOVA for three or more groups of samples, and two-way ANOVA was used for group analysis. Fisher’s least significant difference (LSD) test was used for comparison with control group, and Tukey’s honest significant difference (HSD) test for all pairwise comparison. A *p* value of <0.05 was considered significant.

## Resource availability

### Lead contact

Further information and requests for resources and reagents should be directed to and will be fulfilled by the lead contact, Ellis Kin Lam Fok (ellisfok@cuhk.edu.hk).

### Materials availability

All unique/stable reagents generated in this study are available from the lead contact with a completed materials transfer agreement.

### Data and code availability

• Proteomics data have been deposited at iProX partner repository with the dataset identifier PXD068300. Single-cell RNA-seq and small RNA-seq data have been deposited at GEO accession numbers GSE308837 and GSE308840.
• Any additional information required to reanalyze the data reported in this paper is available from the lead contact upon request.

## Acknowledgments

We are grateful to the members of the Xie laboratory for discussing the progress of the project and providing technical support. We would like to thank the core facilities of the School of Biomedical Sciences, Chinese University of Hong Kong for the help in experiments. This work was supported in part by grants from the Research Grant Council of Hong Kong (T13N-62S), the Direct Grant of CUHK, and the Lo Kwee Seong Start-Up Fund to E.K.L.F.

## Author contributions

T.Z. and E.K.L.F. conceived and designed the study. T.Z. performed the experiments and analyzed the data. K.H.K.C., S.Y.C., M.Z., X.L., H.C., and T.X. provided methodological guidance and analyzed the data. H.C., T.X., and E.K.L.F. provided funding support. E.K.L.F. and T.Z. wrote the manuscript with the help of all the authors. All authors read and approved the final manuscript.

## Declaration of interests

The authors declare no conflict of interest.

## Supplemental information

Data S1. Tables S1–S3

Document S2. Article plus supplemental information
