# Supplementary material for: Extracellular-vesicle-mediated transfer of let-7b/7c promotes the proliferation of transition-state spermatogonia in neonatal mouse testis
Source: Stem Cell Reports. 2025 Oct 23;20(11):102681. doi: 10.1016/j.stemcr.2025.102681 (PMC12790720; doi:10.1016/j.stemcr.2025.102681)
Supplement: Document S1. Figures S1–S6 and Tables S4–S6 [file mmc1.pdf]

Stem Cell Reports, Volume *20*

Supplemental Information

Extracellular-vesicle-mediated transfer of let-7b/7c promotes the proliferation of transition-state spermatogonia in neonatal mouse testis

Tingting Zheng, Kathleen Hoi Kei Choy, Sze Yan Chan, Min Zheng, Xiaotong Luo, Hao Chen, Ting Xie, and Ellis Kin Lam Fok

Supplementary information

Supplementary Figures

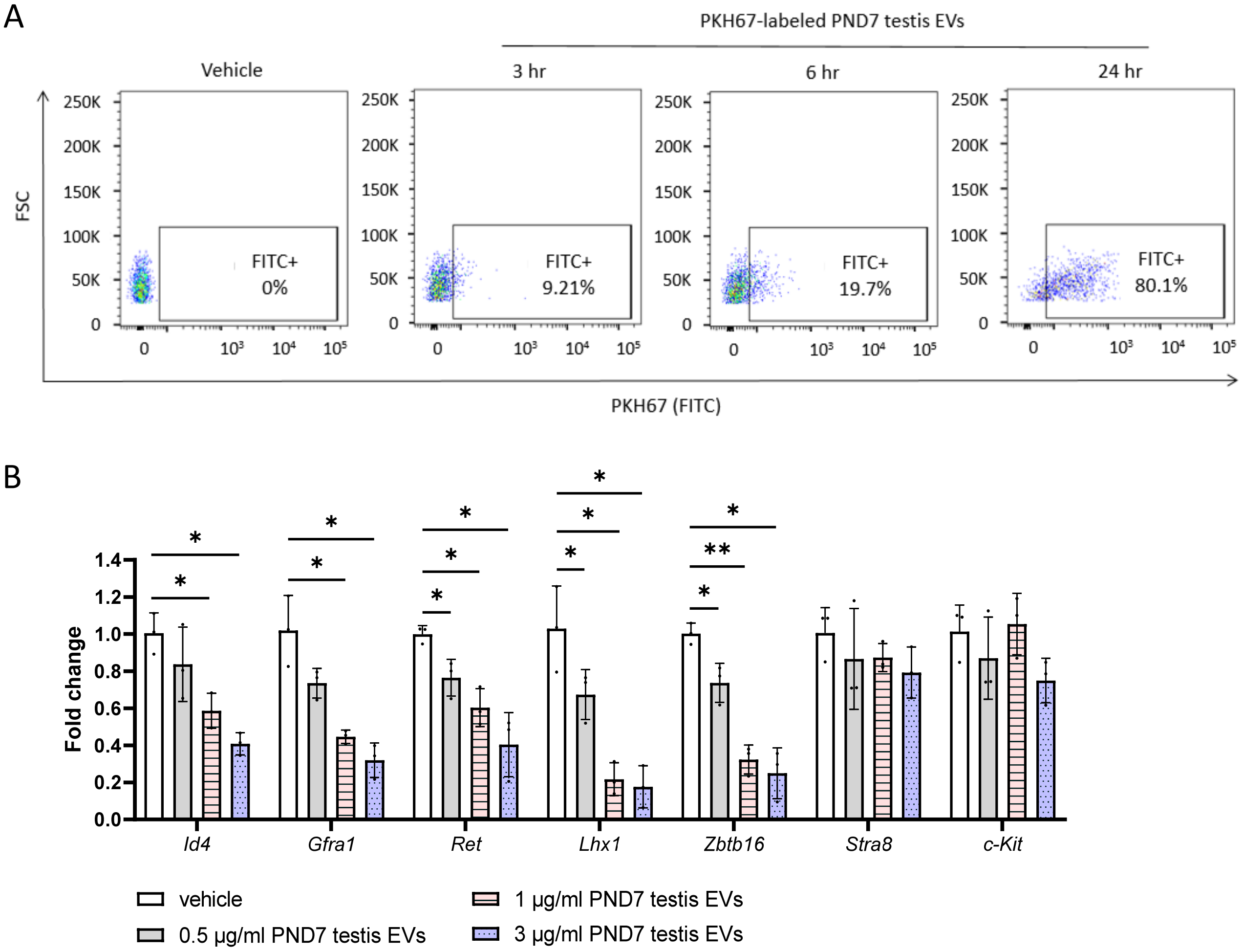

Figure S1. Prim ary spermatogonia efficiently take up PND7 testis EVs, and their expression of undifferentiated spermatogonial markers are reduced in a dose-dependent manner, related to Figure 2.

(A) Representative flow cytometry analysis of primary spermatogonia 3 h, 6 h and 24 h after co-culturing with PKH67-labeled testis EVs. Cultured spermatogonia take up PKH67-labeled testis EVs in a time-dependent manner with more than 80% of spermatogonia showed fluorescent signals after 24 h incubation.

(B) Realtime PCR results showing the expression levels of undifferentiated markers *Id4*, *Gfra1*, *Ret*, *Zbtb16* and *Lhx1* and differentiation markers *Stra8* and *c-Kit* in primary spermatogonia after exposure to indicated dose of PND7 testis EVs. n=3 independent experiments.

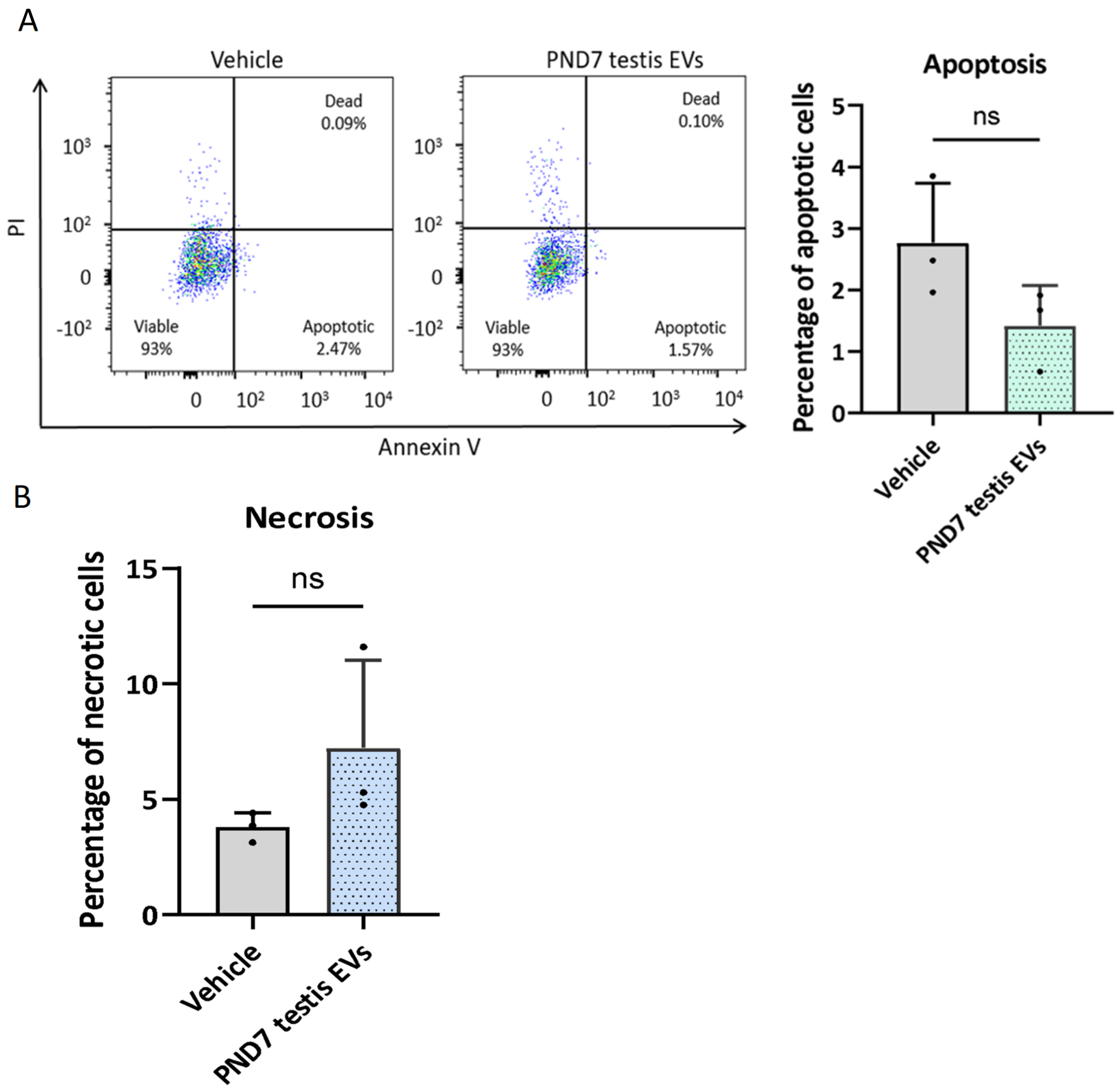

Figure S2. EVs from neonatal testis do not induce cell death in primary spermatogonial culture, related to Figure 1.

(A-B) Representative flow cytometry analysis of Annexin V-stained primary spermatogonia exposed to PND7 testicular EVs (1 µg/ml, n=3 independent experiments). Annexin V-/PI- is viable cells, Annexin V+/PI- represents apoptotic cells, Annexin V+/PI+ indicates dead cells, and Annexin V-/PI+ represents necrotic cells.

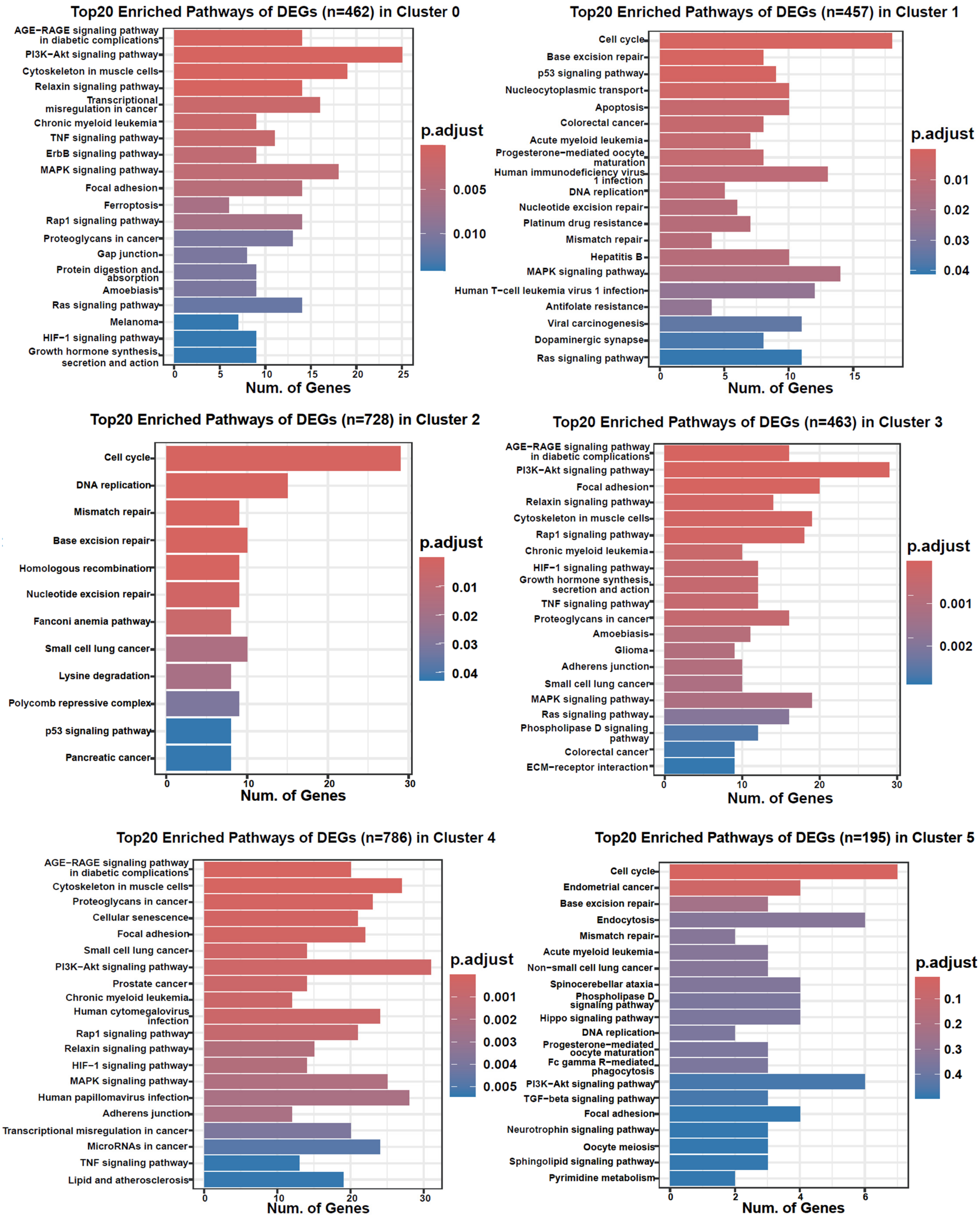
Top20 Enriched Pathways of DEGs (n=462) in Cluster 0
AGE−RAGE signaling pathway in diabetic complications
PI3K−Akt signaling pathway
Cytoskeleton in muscle cells
Relaxin signaling pathway
Transcriptional misregulation in cancer
Chronic myeloid leukemia
TNF signaling pathway
ErbB signaling pathway
MAPK signaling pathway
Focal adhesion
Ferroptosis
Rap1 signaling pathway
Proteoglycans in cancer
Gap junction
Protein digestion and absorption
Amoebiasis
Ras signaling pathway
Melanoma
HIF−1 signaling pathway
Growth hormone synthesis, secretion and action
0 5 10 15 20 25
Num. of Genes
p.adjust
0.005
0.010
Top20 Enriched Pathways of DEGs (n=457) in Cluster 1
Cell cycle
Base excision repair
p53 signaling pathway
Nucleocytoplasmic transport
Apoptosis
Colorectal cancer
Acute myeloid leukemia
Progesterone−mediated oocyte maturation
Human immunodeficiency virus 1 infection
DNA replication
Nucleotide excision repair
Platinum drug resistance
Mismatch repair
Hepatitis B
MAPK signaling pathway
Human T−cell leukemia virus 1 infection
Antifolate resistance
Viral carcinogenesis
Dopaminergic synapse
Ras signaling pathway
0 5 10 15
Num. of Genes
p.adjust
0.01
0.02
0.03
0.04
Top20 Enriched Pathways of DEGs (n=728) in Cluster 2
Cell cycle
DNA replication
Mismatch repair
Base excision repair
Homologous recombination
Nucleotide excision repair
Fanconi anemia pathway
Small cell lung cancer
Lysine degradation
Polycomb repressive complex
p53 signaling pathway
Pancreatic cancer
0 10 20 30
Num. of Genes
p.adjust
0.01
0.02
0.03
0.04
Top20 Enriched Pathways of DEGs (n=463) in Cluster 3
AGE−RAGE signaling pathway in diabetic complications
PI3K−Akt signaling pathway
Focal adhesion
Relaxin signaling pathway
Cytoskeleton in muscle cells
Rap1 signaling pathway
Chronic myeloid leukemia
HIF−1 signaling pathway
Growth hormone synthesis, secretion and action
TNF signaling pathway
Proteoglycans in cancer
Amoebiasis
Glioma
Adherens junction
Small cell lung cancer
MAPK signaling pathway
Ras signaling pathway
Phospholipase D signaling pathway
Colorectal cancer
ECM−receptor interaction
0 10 20 30
Num. of Genes
p.adjust
0.001
0.002
Top20 Enriched Pathways of DEGs (n=786) in Cluster 4
AGE−RAGE signaling pathway in diabetic complications
Cytoskeleton in muscle cells
Proteoglycans in cancer
Cellular senescence
Focal adhesion
Small cell lung cancer
PI3K−Akt signaling pathway
Prostate cancer
Chronic myeloid leukemia
Human cytomegalovirus infection
Rap1 signaling pathway
Relaxin signaling pathway
HIF−1 signaling pathway
MAPK signaling pathway
Human papillomavirus infection
Adherens junction
Transcriptional misregulation in cancer
MicroRNAs in cancer
TNF signaling pathway
Lipid and atherosclerosis
0 10 20 30
Num. of Genes
p.adjust
0.001
0.002
0.003
0.004
0.005
Top20 Enriched Pathways of DEGs (n=195) in Cluster 5
Cell cycle
Endometrial cancer
Base excision repair
Endocytosis
Mismatch repair
Acute myeloid leukemia
Non−small cell lung cancer
Spinocerebellar ataxia
Phospholipase D signaling pathway
Hippo signaling pathway
DNA replication
Progesterone−mediated oocyte maturation
Fc gamma R−mediated phagocytosis
PI3K−Akt signaling pathway
TGF−beta signaling pathway
Focal adhesion
Neurotrophin signaling pathway
Oocyte meiosis
Sphingolipid signaling pathway
Pyrimidine metabolism
0 2 4 6
Num. of Genes
p.adjust
0.1
0.2
0.3
0.4

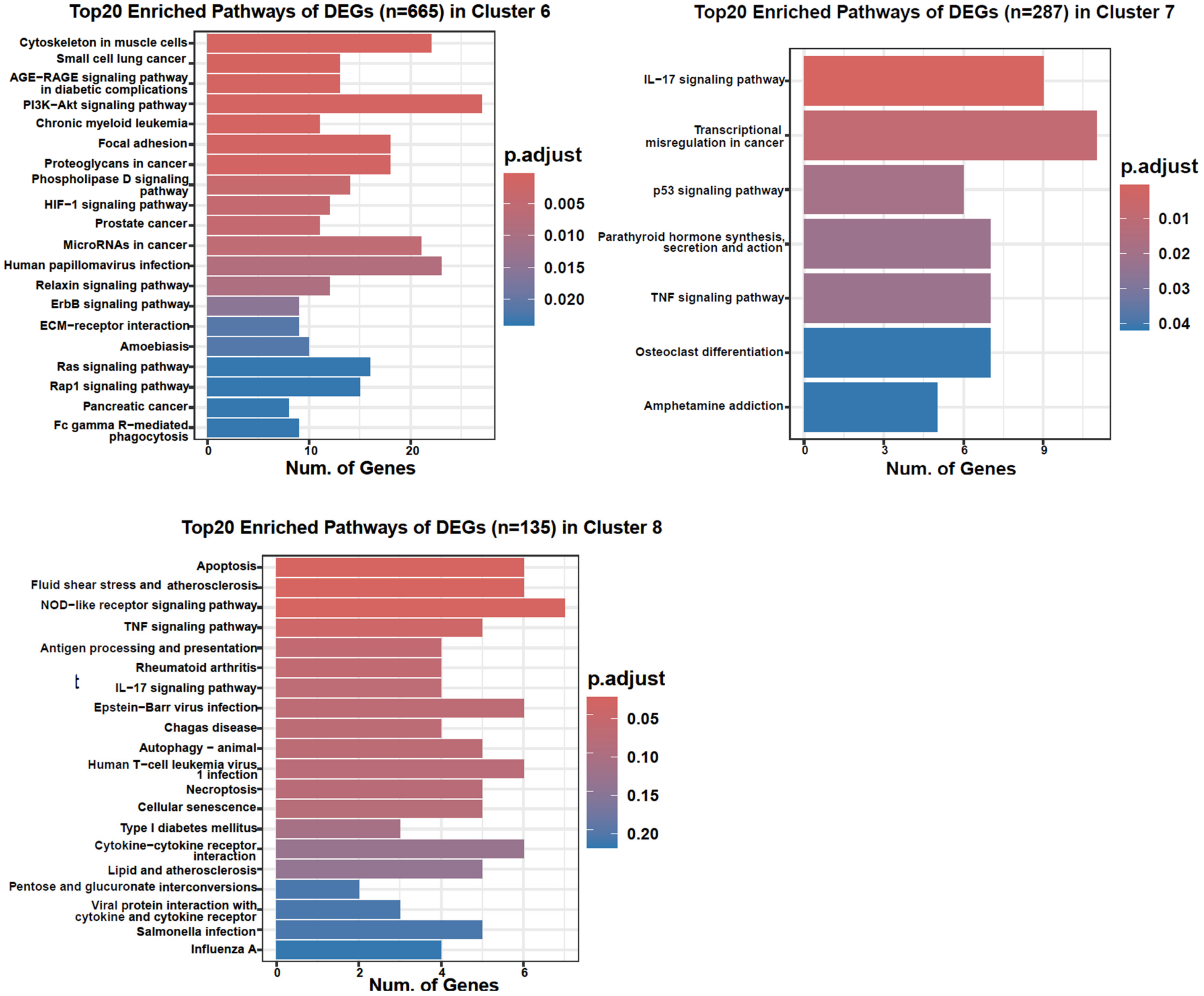

Figure S3. PND7 testicular EVs alter signaling pathways within the same spermatogonial cell cluster, related to Figure 3.

KEGG pathway enrichment analysis of the significantly differentially expressed genes between control and PND7 testicular EV-treated group in each spermatogonial subpopulation clustered by single-cell RNA sequencing, n=2 independent experiments.

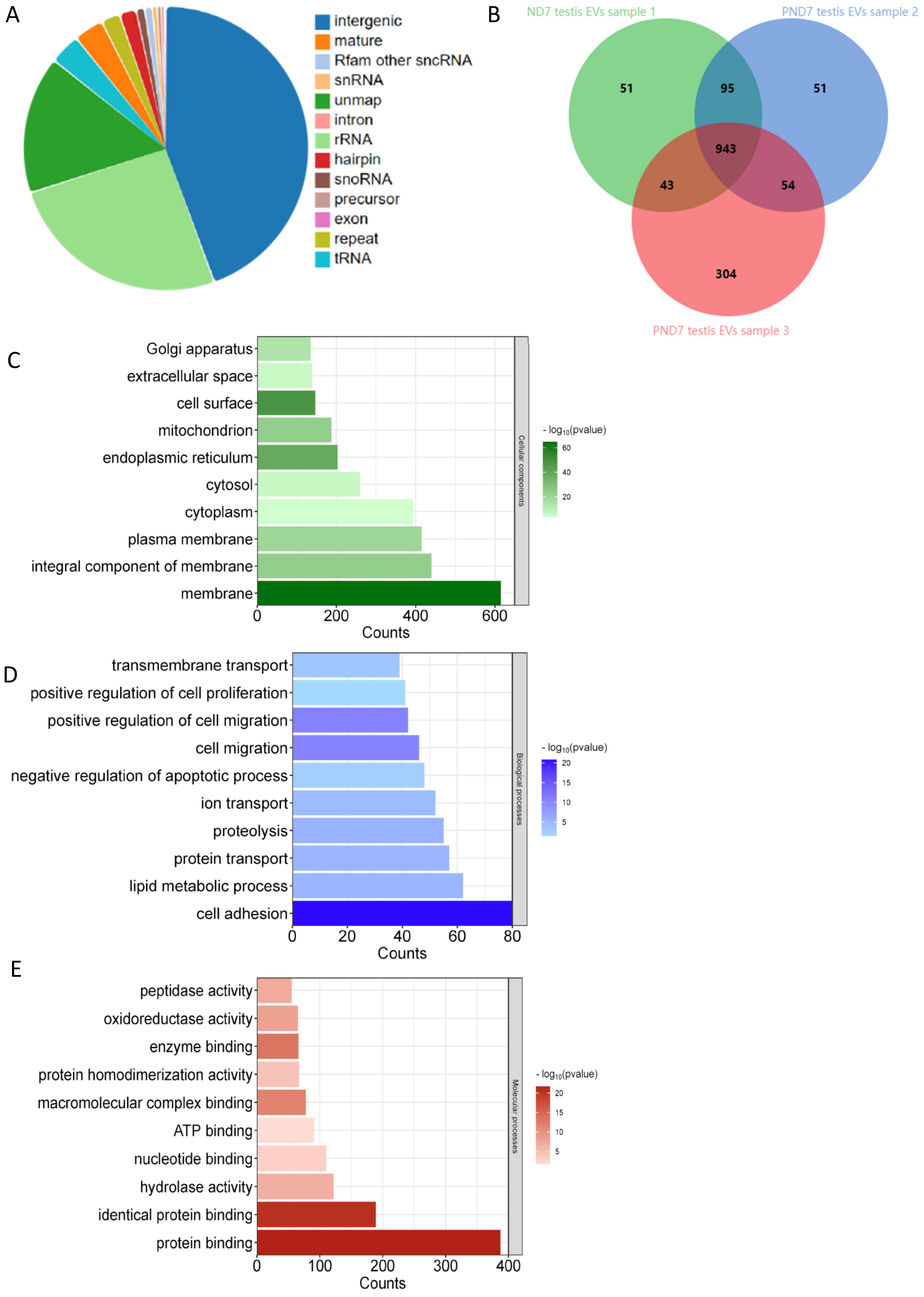
A
intergenic
mature
Rfam other sncRNA
snRNA
unmap
intron
rRNA
hairpin
snoRNA
precursor
exon
repeat
tRNA
B
ND7 testis EVs sample 1
PND7 testis EVs sample 2
PND7 testis EVs sample 3
51
95
51
943
43
54
304
C
Golgi apparatus
extracellular space
cell surface
mitochondrion
endoplasmic reticulum
cytosol
cytoplasm
plasma membrane
integral component of membrane
membrane
0
200
400
600
Counts
Cellular components
- log10(pvalue)
60
40
20
D
transmembrane transport
positive regulation of cell proliferation
positive regulation of cell migration
cell migration
negative regulation of apoptotic process
ion transport
proteolysis
protein transport
lipid metabolic process
cell adhesion
0
20
40
60
80
Counts
Biological processes
- log10(pvalue)
20
15
10
5
E
peptidase activity
oxidoreductase activity
enzyme binding
protein homodimerization activity
macromolecular complex binding
ATP binding
nucleotide binding
hydrolase activity
identical protein binding
protein binding
0
100
200
300
400
Counts
Molecular processes
- log10(pvalue)
20
15
10
5

Figure S4. The repertoire of small RNA and protein cargoes in the EVs isolated from neonatal testis.

(A) Pie chart showing the distribution of different species of small RNAs identified in PND7 testicular EVs by RNA sequencing (n=3 samples, each sample represents the pooled testicular EVs isolated from 10 mice).

(B) Venn analysis showed 943 identified proteins in PND7 testicular EVs (n=3 samples, each sample represents the pooled testicular EVs isolated from 3 mice).

(C-E) Gene Ontology analysis on (C) cellular component, (D) biological process and (E) molecular function of proteins identified in PND7 testicular EVs.

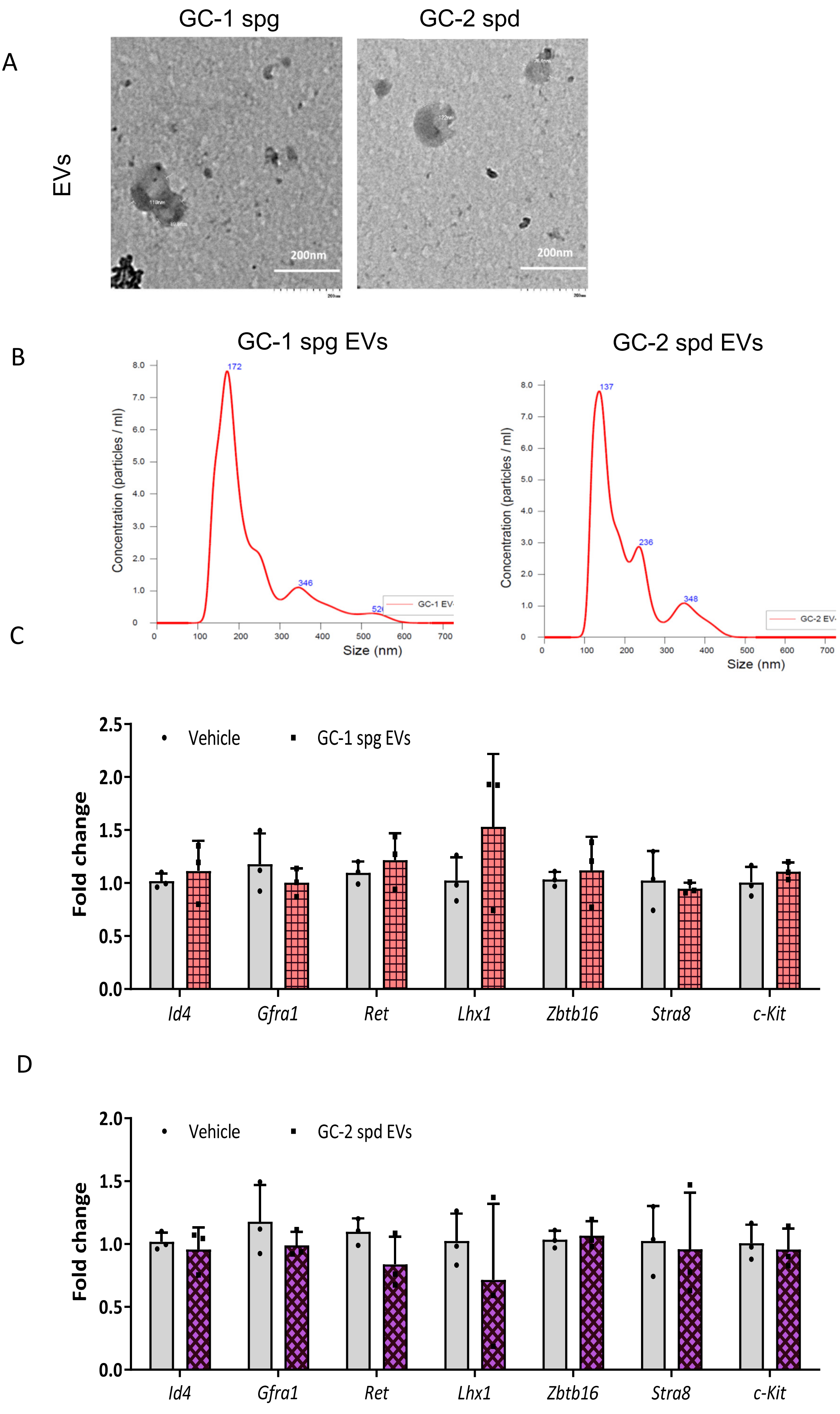
A
GC-1 spg
GC-2 spd
EVs
200nm
200nm
B
GC-1 spg EVs
GC-2 spd EVs
Concentration (particles / ml)
172
346
GC-1 EV
Size (nm)
137
236
348
GC-2 EV
Size (nm)
C
Vehicle
GC-1 spg EVs
Fold change
Id4
Gfra1
Ret
Lhx1
Zbtb16
Stra8
c-Kit
D
Vehicle
GC-2 spd EVs
Fold change
Id4
Gfra1
Ret
Lhx1
Zbtb16
Stra8
c-Kit

Figure S5. EVs isolated from differentiated germ cell lines pose negligible effect on the expression of spermatogonia markers in primary spermatogonial culture, related to Figure 4.

(A) Representative transmission electron microscopy image of EVs isolated from GC1-spg and GC2-spd cell lines by ultracentrifugation. Scale bar: 200 nm.

(B) Size distribution of cell line-derived EVs isolated as in (A) determined by nanoparticle tracking analysis.

(C-D) Real-time PCR results showing the expression of SSCs marker *Id4*, As spermatogonia marker *Gfra1* and pan-undifferentiated spermatogonia markers *Zbtb16*, *Ret* and *Lhx1*, and spermatogonial differentiation markers *Stra8* and *c-Kit* in primary spermatogonia after treatment with EVs isolated from GC1-spg (C) and GC2-spd (D) cell lines (1 μg/ml). Data is presented as mean ± S.D. *$P<0.05$, **$P<0.01$, ***$P<0.001$, by t-test; n=3 independent experiments.

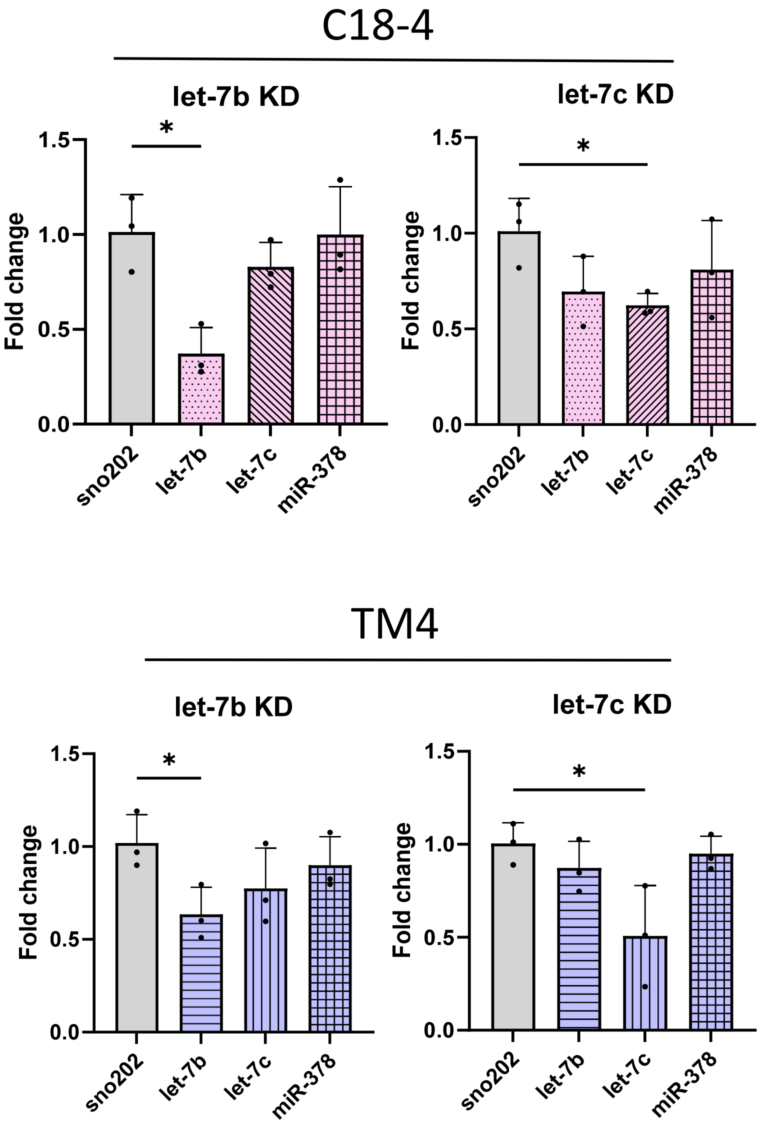

Figure S6. Knockdown of let-7b and 7c in C18-4 and TM4 cell lines, related to Figure 6.

Real-time PCR results showing the levels of let-7b, let-7c and miR378 in wild type (WT) and mutants (let-7b KD and let-7c KD) C18-4 or TM4 cell lines. Data is presented as mean ± S.D. *P<0.05, by t-test; n=3 independent experiments.

Supplemental tables

Table S1. List of top 50 most abundant miRNAs in PND7 testicular EVs and their functions on stem cells.

Table S2. List of proteins identified in PND7 testicular EVs.

Table S3. Protein levels of exosome protein markers in PND7 testicular EVs.

Table S4. Functions of genes encoding proteins identified in PND7 testicular EVs on spermatogenesis or male fertility.

Accession	Gene name	Description	Average		Functions
			#Unique	-10lgP	
P16406|AMPE_MOUSE	Enpep	Glutamyl aminopeptidase	31	389.96	male fertility
P43406|ITAV_MOUSE	Itgav	Integrin alpha-V	27	354.19	Expressed on the surface o f porcine SSCs
Q60675|LAMA2_MOUSE	Lama2	Laminin subunit alpha-2	24	309.88	Sertoli cell functions
Q61739|ITA6_MOUSE	Itga6	Integrin alpha-6	21.67	360.33	SSC marker
Q8BTM8|FLNA_MOUSE	Flna	Filamin-A	21.33	297.93	SSC proterty
P20029|BIP_MOUSE	Hspa5	Endoplasmic reticulum ch aperone BiP	19.67	339.41	male fertility
P02468|LAMC1_MOUSE	Lamc1	Laminin subunit gamma-1	19	321.01	stem cell maintenance
Q3U1J4|DDB1_MOUSE	Ddb1	DNA damage-binding protein 1	17.33	250	SSC maintenance<br>Sertoli cell proliferation
Q9WV91|FPRP_MOUSE	Ptgfrn	Prostaglandin F2 receptor negative regulator	17	302.75	male fertility
Q60597|ODO1_MOUSE	Ogdh	2-oxoglutarate dehydrogena se mitochondrial	15	260.69	hESC maintenance
P57780|ACTN4_MOUSE	Actn4	Alpha-actinin-4	13.67	311.71	CSC property
Q3UHK6|TEN4_MOUSE	Tenm4	Teneurin-4	13.67	252.34	CSC property
P27773|PDIA3_MOUSE	Pdia3	Protein disulfide-isomerase A3	13	273.57	male fertility
P19096|FAS_MOUSE	Fasn	Fatty acid synthase	12.67	259.96	NSPC activity
Q61738|ITA7_MOUSE	Itga7	Integrin alpha-7	12	265.42	stem cell property
Q61503|5NTD_MOUSE	Nt5e	5'-nucleotidase	11.67	274.42	spermatogenesis
P09055|ITB1_MOUSE	Itgb1	Integrin beta-1	11.67	256.26	SSC marker
P62259|1433E_MOUSE	Ywhae	14-3-3 protein epsilon	11.33	257.45	male fertility
P07356|ANXA2_MOUSE	Anxa2	Annexin A2	11.33	243.76	male fertility
P23249|MOV10_MOUSE	Mov10	Putative helicase MOV-10	11	263	male fertility
Q62469|ITA2_MOUSE	Itga2	Integrin alpha-2	10.67	255.04	stem cell property
Q8BKG3|PTK7_MOUSE	Ptk7	Inactive tyrosine-protein kinase 7	10.67	236.13	stem cell property
P47738|ALDH2_MOUSE	Aldh2	Aldehyde dehydrogenase mitochondrial	10.33	278.15	stem cell property
P42703|LIFR_MOUSE	Lifr	Leukemia inhibitory facto r receptor	10.33	233.89	male fertility
Q64521|GPDM_MOUSE	Gpd2	Glycerol-3-phosphate dehydrogenase mitochondria	10	231.68	male fertility

Note:
1. Enriched genes are reported to orchestrate SSC properties (red words) or stem cell properties (blue words).
2. CSC, cancer stem cell; hESC, human embryonic stem cell; NSPC, neural stem and progenitor cell; SSC, spermatogonial stem cell.

Table S5. List of TaqMan probes used for real time PCR analysis of candidate miRNAs, related to Figure 5.

Primer name	Mature sequence	Assay ID
hsa-miR-125b	5'-UCCCUGAGACCCUAACUUGUGA-3'	ThermoFisher, 000449
hsa-miR-21	5'-UAGCUUAUCAGACUGAUGUUGA-3'	ThermoFisher, 000397
hsa-miR-34a	UGGCAGUGUCUUAGCUGGUUGU-3'	ThermoFisher, 000426
hsa-miR-378	5'-ACUGGACUUGGAGUCAGAAGG-3'	ThermoFisher, 002243
mmu-miR-451	5'-AAACCGUUACCAUUACUGAGUU-3'	ThermoFisher, 001141
hsa-miR-652	5'-AAUGGCGCCACUAGGGUUGUG-3'	ThermoFisher, 002352
hsa-let-7a	5'-UGAGGUAGUAGGUUGUAUAGUU-3'	ThermoFisher, 000377
hsa-let-7b	5'-UGAGGUAGUAGGUUGUGUGGUU-3'	ThermoFisher, 000378
hsa-let-7c	5'-UGAGGUAGUAGGUUGUAUGGUU-3'	ThermoFisher, 000379
hsa-let-7f	5'-UGAGGUAGUAGAUUGUAUAGUU-3'	ThermoFisher, 000382
snoRNA202	5'-GCTGTACTGACTTGATGAAAGTACTTTTGAACCCTTTTCCATCTGATG-3'	ThermoFisher, 001232
U6 snRNA	5'- GTGCTCGCTTCGGCAGCACATATACTAAAATTGGAACGATACAGAGAAGATTAGCATGGCCCCTGCGCAAGGATGACACGCAAATTCGTGAAGCGTTCCATATTTT-3'	ThermoFisher, 001973

Table S6. List of primers and oligos used in this study, related to Figure 2and 4.

Primer name	Sequence	Tm
mId4 F	5’-TGATATGCGCACTCTAACCGT-3’	66°C
mId4 R	5’-CGATCCCTGAACATGTCCATCAG-3’	67°C
mGfra1 F	5’-TTCTTGCAGGTGTCATCCAG-3’	65 °C
mGfra1 R	5’-GATATATTCCGGGCAGTCCC-3’	65 °C
mPlzf F	5’-CGTGCGCAGCTATATTTGCA-3’	66°C
mPlzf R	5’-TGGCTCTTGAGTGTGCTCTCA-3’	68°C
mStra8 F	5’-CATCATCACTGGGTTGGTTG-3’	64°C
mStra8 R	5’-CTGCGTGTTCCACAAGTGTC-3’	67°C
m-c-kit F	5’-GGCCTCACGAGTTCTATTTACG-3’	65°C
m-c-kit R	5’-GGGGAGAGATTTCCCATCACAC-3’	67°C
m-c-Ret F	5’-CCGTAGGGCATGGACATAGA-3’	66°C
m-c-Ret R	5’-GCTGAAGCTGATTTTGCTCC-3’	64°C
mLhx F	5’-AACCAGATCGCTTGGAGAGA-3’	66°C
mLhx R	5’-TGTAAATGCAACCTGACCGA-3’	64°C
mNgn3-rt F	5’-TGGCGCCTCATCCCTTGGATG-3	73°C
mNgn3-rt R	5’-CAGTCACCCACTTCTGCTTCG-3	68°C
mLin28-rt F	5’-AAGATCCAAAGGAGACAGGTGC-3’	67°C
mLin28-rt R	5’-GGAAGTAGGCAGGCTTTCCC-3’	68°C
mRara F	5’-TGTAAGGGCTTCTTCCGACGA-3’	68°C
mRara R	5’-GCTTGGGTGCCTCTTTCTTC-3’	66°C
mRarg F	5’-TGCCTGGTTTTACAGGGCTC-3’	60°C
mRarg R	5’-TCCGAGAATGTCATAGTGTCCT-3’	56°C
mDdx4 F	5’-GCTTCATCAGATATTGGCGAGT-3’	65°C
mDdx4 R	5’-GCTTGGAAAACCCTCTGCTT-3’	66°C
GAPDH F	5’-GACCACAGTCCATGCCATCACTGC-3’	73°C
GAPDH R	5’-GCTGTTGAAGTCGCAGGAGACAAC-3’	70°C
LeGO-PCR-F1	5’-AATATGCATGCGAGAAAAGCCTTG-3’	59°C
LeGO-PCR-F2	5’-GTTTTAGAGCTAACCGGTTAGCAAGT-3’	59°C
LeGO-PCR-R2	5’-TACCACCACACTGGGATCC-3’	57°C
glet7b-48 oligo1	5’-cgagaaaagccttgtttgGGTTGTATAGTTATCTTCGGgttttagagctaa-3’	/
glet7b-48 oligo2	5’-ccggttagctctaaaacCCGAAGATAACTATACAACCcaaacaaggcttttctc-3’	/
glet7c-49 oligo1	5’-cgagaaaagccttgtttgAGGTTGTACAGTTAACTCCCgttttagagctaa-3’	/
glet7c-49 oligo2	5’-ccggttagctctaaaacGGGAGTTAACTGTACAACCTcaaacaaggcttttctc-3’	/

Supplemental experimental procedures

Adipose-derived EVs isolation

Adipose-derived EVs were isolated from mouse visceral adipose tissue, as previously reported (Wei et al., 2020). Tissue samples were washed in PBS, minced into ~2 mm^3 fragments, and incubated in serum-free medium at 37°C for 24 h. The resulting conditioned medium was collected, and EVs were isolated using differential ultracentrifugation, as previously described. Briefly, CCM was centrifuged at 300g 10 min to remove live cells and 3000g 20 min to remove cell debris, followed by 10,000g 30 min 4°C (Beckman Avanti J-E Centrifuge) to remove large vesicles. Lastly, the supernatant was ultracentrifuged (Beckman Optima XPN-100 Ultra-High Speed Refrigerated Centrifuge) at 100,000g 4°C for 90 min to pellet the small vesicles. The pellets were washed by PBS with ultracentrifuge (Hitachi CS150FNX Ultra-Microcentrifuge) at 100,000g 4°C for 90 min. The EV pellet was resuspended in 20 µl PBS and stored at -80°C for further analysis.

Transmission electron microscope (TEM)

The morphology of EVs was observed using a transmission electron microscope. Briefly, EVs were resuspended in 20 µl PBS and fixed with 2% paraformaldehyde until use. 10 µl EVs were added onto the formvar grid (200 mesh) for 30–60 min, and excess fluid was removed with filter paper. EVs were fixed with 1% glutaraldehyde for 10 min, followed by negative staining with 2% uranyl acetate for 2 min, and three images were captured at different fields using a Hitachi H-7700 transmission electron microscope.

Nanoparticle tracking analysis (NTA)

The concentration of EVs was measured by the Nanosight LM14C (Malvern) instrument. Briefly, EV samples were diluted with filtered PBS and the particle concentration was adjusted to the range of ~107-109 particles/ml, which allows the viewing of approximately 20-100 particles in each field. A thirty-second video was recorded of each sample and captured at 3 different fields. The concentration of testis EVs was analyzed and calculated by the NanoSight software using the Stokes-Einstein equation.

Uptake of PKH67-labeled testis EVs

Purified EVs were labeled using the PKH67 Green Fluorescent Cell Linker Mini Kit (Sigma-Aldrich) according to the manufacturer's instructions. Briefly, 100 µl of EVs resuspended in PBS were mixed with 1 ml Diluent C and 6 µl PKH67 dye, followed by 5-min incubation. Excess dye was quenched by adding 2 mL of 0.5% BSA/PBS, and 20 ml PBS was added to prevent vesicle aggregation during ultracentrifugation. Labeled EVs were washed via ultracentrifugation and resuspended in 50 µl PBS. Primary spermatogonia were co-cultured with labeled EVs for 3, 6, and 24 h, and EV uptake was analyzed by flow cytometry.

Proliferation assay

The culture medium was removed and replaced with fresh medium containing 10 µM BrdU for 12 hours at 37ºC. After incubation, spermatogonial clumps were collected by flushing from the feeder cells as described (Fok et al., 2017). The clumps were pelleted by centrifugation at 300g for 5 min. Then, the clumps were trypsinized into a single-cell suspension (Gibco). The cells

were washed twice with PBS and then fixed by dropwisely adding 5 ml ice-cold 70% ethanol and incubated at -20°C for 2h. After fixation, the cells were permeabilized with 2 M HCl/0.5% Triton X-100 at room temperature for 30 minutes. After washing twice with PBS supplemented with 1% BSA, cells were incubated with the anti-BrdU antibody (Abcam, ab6326, 1:100) and anti-DDX4 antibody (Abcam, ab13840, 1:100) in the solution of 1% BSA in PBS (add 1 g of BSA to 100 ml of PBS) at room temperature for 1 hour. After three times washes with the solution of 1% BSA in PBS, the samples were stained with Alexa Fluor 488 anti-rat IgG (H+L) (Invitrogen, A21208,1:1000) and Alex Fluor 647 anti-rabbit IgG (H+L) (Invitrogen, A31573, 1:1000) and incubated at room temperature for 1 hr. Lastly, the cells were washed with the solution of 1% BSA in PBS three times and resuspended in the solution of 1% BSA in PBS to 106-107 cells/ml and analyzed flow cytometry (BD LSRFortessa™ Cell Analyzer). The results were analyzed by FlowJo_V10.

Single-cell RNA sequencing

Cell clumps were digested into single cells by 0.05% trypsin. Cells were washed thrice with 0.04% BSA in PBS and resuspended in the 0.04% BSA in PBS. Cell concentration and viability were determined using a Countess® II Automated Cell Counter. The cell concentration should be 1000 cells/μl and more than 85% viability. Then, the single-cell library was prepared according to the 10x Genomics® Single Cell Protocol.

The input dataset was aligned with the mouse genome (mm10, GENCODE vM23/Ensembl 98) as appropriate and used the Cell Ranger v.7.0.1 Single-Cell Software Suite from 10x Genomics to estimate partitions containing cells and their unique molecular identifiers (UMIs). The Seurat R package was used for downstream analyses. Additional quality control measures were applied based on the following criteria: 1) the number of expressed genes must be greater than 400 and less than 4,000; 2) cells should have mitochondrial RNA content below 10%. The DoubletFinder R package was employed to remove potential doublets. The filtered gene expression matrix for each sample was normalized and scaled using the “NormalizeData” and “ScaleData” functions in Seurat. Ultimately, we identified 23904 genes and detected 29853 cells across four samples. We performed principal component analysis (PCA) on the corrected expression matrix, focusing on highly variable genes (HVGs) identified by the “FindVariableFeatures” function. Afterward, we conducted PCA using the “RunPCA” function and constructed a K-nearest-neighbor graph with the “FindNeighbors” function. The most representative principal components were then used to classify different cell types with the “FindClusters” function.

To comprehensively annotate the specific types of cell clusters, we examined the expression of the following marker genes and performed hierarchical clustering analysis using the R package pheatmap: Zbtb16, Lhx1, Bcl6b, Etv5, and Ret for SCC cells; Rara, Upp1, and Rarg for progenitor cells; and Col1a1, Col4a1, Aifm2, Epcam, Pdgfra, Pdpn, Ly6c1, Ly6c2, and Cd24a for fibroblasts. In addition, we investigated the lineage relationship among three myofibroblast subtypes using Monocle2. Furthermore, we assessed the cell cycle using the CellCycleScoring function in the Seurat R package, which utilizes the cell cycle genes provided by Seurat. Based on the scores of these cell cycle genes, we classified each cell's phase as follows: if both the S

and G2M phase scores are less than 0, the cell is categorized as being in the G1 phase. If at least one score is greater than 0, the cell is assigned to the phase corresponding to the higher score.

To identify differentially expressed genes between Vehicle samples and PND7 testis EV samples for each cell subtype, we used the "FindMarkers" function (for two-condition comparisons) from the Seurat package with default parameters. A gene was considered differentially expressed if it had an adjusted P-value < 0.05 and an absolute log2 (fold change) > 1. We used the R package clusterProfiler to conduct KEGG pathway enrichment analysis on the significantly differentially expressed genes.

Small RNA sequencing

RNA was extracted from EVs after treatment with proteinase K, PMSF and RNase A using miRNeasy Mini Kit as described above. Small-RNA libraries were prepared, and the PCR products were sequenced using BGISEQ-500 technology. Small RNA sequencing was performed by BGI (Shenzhen, China). After eliminating the low-quality reads, clean reads were mapped to reference genome and to other sRNA databases using Bowtie2 and cmsearch. Classification of sRNA follows the priority rule: MiRbase> pirnabank> snoRNA(human/plant)> Rfam> other sRNA to ensure a unique map of each entry. Novel miRNAs and piRNAs were predicted using miRDeep2 and Piano, respectively.

Mass spectrometer proteomic analysis

Testis EV proteins were extracted with RIPA buffer, and protein concentrations were detected by BCA assay. 6 μg of proteins in each sample were used to perform Bruker timsTOF Pro Mass Spectrometer proteomics analyses by Biosciences Central Research Facility of the Hong Kong University of Science and Technology. Gene ontology (GO) enrichment and KEGG pathway enrichment were analyzed.

Generation of let-7b and let-7c mutant cell lines

The let-7b and le-7c mutant cell lines were generated by the CRISPR/Cas9 system. The sequences of miRNA precursor were downloaded from miRBase v16.0 (http://www.mirbase.org/) and sgRNAs were designed by the online tool CRISPR DESIGN (http://crispr.mit.edu/). Designs were selected by specificity score. Due to the sequence homology and short sequence of the let-7 family, designs that target the seed sequence were not available. Thus, designs that target the Dicer processing sites of precursor miRNAs, which alter the biogenesis of let-7b and let-7c, were used. Oligoes were purchased from Integrated DNA Technologies and their sequences are shown in Table S2. The CRISPR/Cas9 and sgRNAs were delivered to target cells by lentiviral transduction.

Supplemental references

Wei, M., Gao, X., Liu, L., Li, Z., Wan, Z., Dong, Y., Chen, X., Niu, Y., Zhang, J., and Yang, G. (2020). Visceral Adipose Tissue Derived Exosomes Exacerbate Colitis Severity via Pro-inflammatory MiRNAs in High Fat Diet Fed Mice. ACS Nano 14, 5099–5110. https://doi.org/10.1021/acsnano.0c01860.

Fok, K.L., Bose, R., Sheng, K., Chang, C.-W., Katz-Egorov, M., Culty, M., Su, S., Yang, M., Ruan, Y.C., Chan, H.C., et al. (2017). Huwe1 regulates the establishment and maintenance of spermatogonia by suppressing DNA damage response. Endocrinology 158, 4000–4016. https://doi.org/10.1210/en.2017-00396.
