# Supplementary material for: Extracellular-vesicle-mediated transfer of let-7b/7c promotes the proliferation of transition-state spermatogonia in neonatal mouse testis
Source: Stem Cell Reports. 2025 Oct 23;20(11):102681. doi: 10.1016/j.stemcr.2025.102681 (PMC12790720; doi:10.1016/j.stemcr.2025.102681)
Supplement: Document S2. Article plus supplemental information [file mmc3.pdf]

Stem Cell Reports
Article
OPEN ACCESS

Extracellular-vesicle-mediated transfer of let-7b/7c promotes the proliferation of transition-state spermatogonia in neonatal mouse testis

Tingting Zheng,[1] Kathleen Hoi Kei Choy,[1] Sze Yan Chan,[1] Min Zheng,[2] Xiaotong Luo,[3,*] Hao Chen,[4,*] Ting Xie,[2,*] and Ellis Kin Lam Fok[1,5,6,*]

[1]School of Biomedical Sciences, Faculty of Medicine, The Chinese University of Hong Kong, Hong Kong SAR, China
[2]Center for Tissue Regeneration and Engineering, JC STEM Laboratory for Regenerative Biology, Division of Life Science, School of Science, The Hong Kong University of Science and Technology, Hong Kong SAR, China
[3]Guangdong Institute of Gastroenterology, Biomedical Innovation Center, The Sixth Affiliated Hospital, Sun Yat-sen University, Guangzhou, China
[4]Guangzhou Medical University, Guangzhou, China
[5]Sichuan University-The Chinese University of Hong Kong Joint Laboratory for Reproductive Medicine, West China Second University Hospital, Chengdu, China
[6]Lead contact
*Correspondence: luoxt36@mail.sysu.edu.cn (X.L.), chenhao@gzhmu.edu.cn (H.C.), tgx@ust.hk (T.X.), ellisfok@cuhk.edu.hk (E.K.L.F.)
https://doi.org/10.1016/j.stemcr.2025.102681

SUMMARY

The self-renewal and differentiation of spermatogonial stem cells (SSCs) play essential roles in spermatogenesis. Extracellular vesicle (EV) is a universal strategy for intercellular communications in stem cell niches. However, the involvement of EVs in regulating SSCs remains largely unknown. This study revealed that testis EVs from postnatal day 7 (PND7) neonatal mouse testis guided spermatogonia into a transit-amplifying state with increased proliferation while retaining their differentiation potential. We profiled the repertoires of proteins and small RNAs by proteomic and small RNA transcriptomic analyses, respectively. We further showed that the EVs secreted by undifferentiated spermatogonia and the Sertoli cell lines, but not from more differentiated germ cell lines, conveyed let-7b/7c microRNA (miRNA) cargoes to spermatogonia, which mediated the effect of EVs on spermatogonial transit amplification. Together, this study has deciphered crucial let-7b/7c cargoes of EV-mediated communication within the spermatogonial niche, providing a new insight into the regulation of SSCs and spermatogenesis.

INTRODUCTION

Spermatogonial stem cells (SSCs) are the most primitive spermatogonia that support spermatogenesis through self-renewal and differentiation. Emerging evidence demonstrates that the delicate balance between self-renewal and differentiation of SSCs is strongly associated with the surrounding microenvironment, also termed a "niche."

The SSC niche is supported by the germ cells and Sertoli cells within seminiferous tubules, which provide structural support, form the blood-testis barrier (BTB) (Oatley and Brinster, 2012), and secrete factors for the self-renewal (Ishii et al., 2012; Kubota et al., 2004; Yang et al., 2013) and differentiation of SSCs (Vernet et al., 2006). Somatic cells like peritubular myoid cells, Leydig cells, interstitial macrophages, and vasculature in the interstitial space also contribute to SSC maintenance (Oatley et al., 2009; Wang et al., 2015).

Extracellular vesicles (EVs) are known to act as messengers in intercellular communication via delivering cargoes, including DNAs, RNAs, proteins, and lipids from donor cells to the recipient cells (Welsh et al., 2024). Several studies have reported that EVs originating from specific cellular components of the SSC niche contribute to SSC regulation. For instance, Thy1^{+} EVs secreted by spermatogonia have been identified in the testicular microenvironment and to suppress the proliferation of SSCs (Lin et al., 2020). The EVs derived from primary Sertoli cells protect SSCs from oxidative stress and promote the proliferation and differentiation of SSCs via microRNA (miRNA) cargoes miR-486-5p and miR-30a-5p (Salek et al., 2021; Wang et al., 2023; Li et al., 2021). Nevertheless, the effects of EVs in regulating SSCs and the cargoes of EVs mediating these effects under a physiological condition remain largely unexplored. We hypothesize that EVs mediate intercellular communication within the SSC niche and that their cargos regulate SSC fate decisions.

This study comprehensively characterized the small RNA and protein cargoes of testis EVs at postnatal day 7, revealing their direct regulation of SSC proliferation and fate decision. Furthermore, we identified specific EV cargoes, let-7b/c miRNAs, as candidate regulators of SSC fate decisions that coordinate niche crosstalk between SSCs and supporting cells.

RESULTS

Postnatal day 7 testis EVs promote spermatogonial proliferation

We first asked if EVs isolated from the testis at postnatal day 7 (PND7), where most spermatogonial subpopulations are established, pose any effect on the SSCs. We isolated EVs from the whole decapsulated PND7 mouse testis

Published by Elsevier Inc. on behalf of International Society for Stem Cell Research.
This is an open access article under the CC BY license (http://creativecommons.org/licenses/by/4.0/).

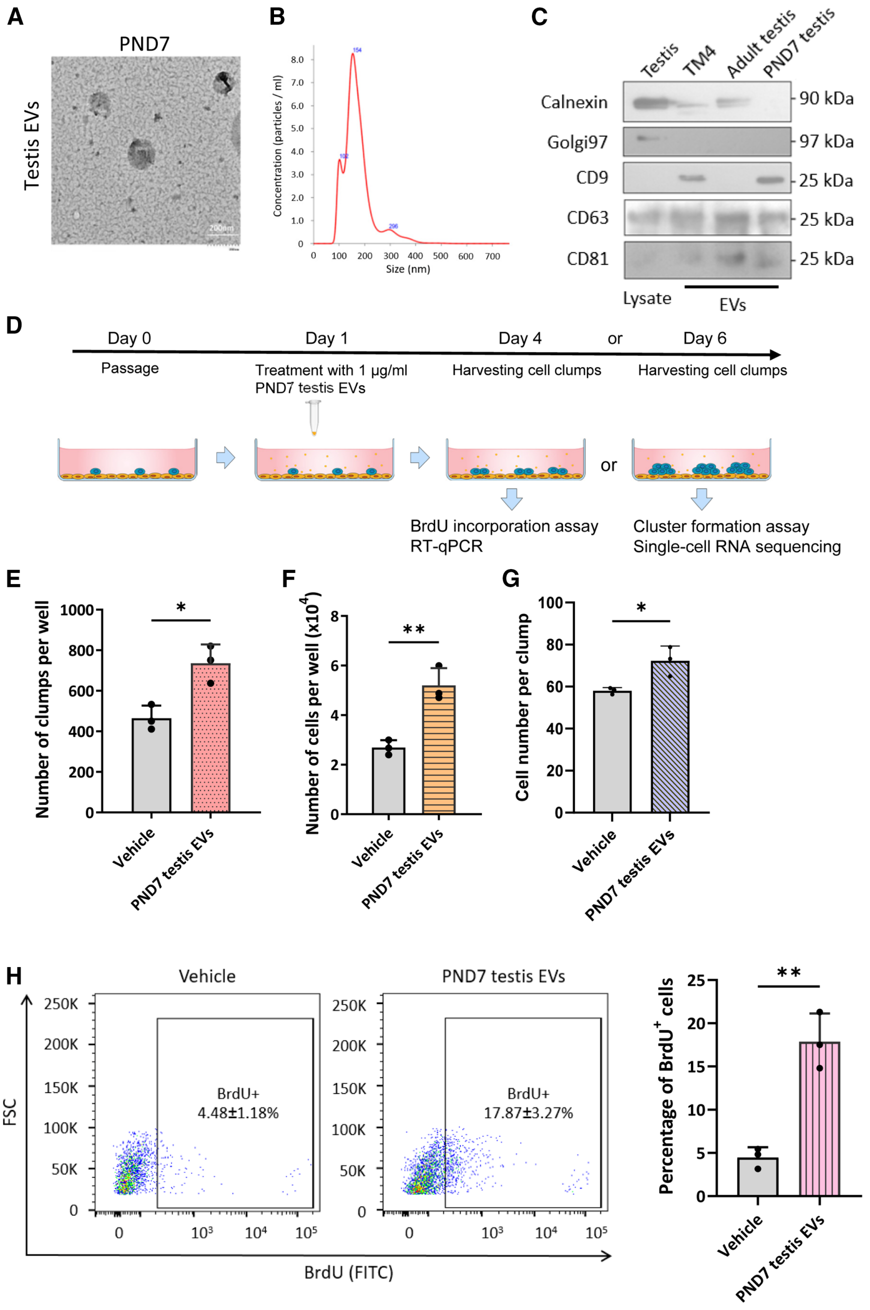

Figure 1. EVs in neonatal testis promote spermatogonial proliferation

(A) Representative transmission electron microscopy image of PND7 mouse testis EVs by affinity columns. Scale bars, 200 nm.

(B) Size distribution of PND7 testis EVs isolated as in (A) determined by nano-particle tracking analysis.

(C) Representative western blot analysis of EV markers CD9, CD63, and CD81 and endoplasmic reticulum markers calnexin and Golgi marker golgin 97 in the PND7 testis EVs and TM4-derived EVs. Protein lysate from mouse testes was used as control.

(D) Schematic diagram showing the treatment protocol of PND7 testis EVs on primary spermatogonial culture. Counting of the numbers of SSC clumps (E) and total cell number of primary spermatogonia (F) after exposure to PND7 testis EVs (1 μg/mL) for 5 days.

(G) The ratio of SSC clumps per 1,000 spermatogonial cells.

(H) Representative flow cytometry analysis of BrdU uptake in primary spermatogonia exposed to PND7 testis EVs for 6 days (1 μg/mL) (see also Figure S2). *n* = 3 independent experiments.

at a stage where spermatogonia are the primary germ cells to minimize heterogeneity of EV source, using our recently developed protocol (Choy et al., 2022). This facilitates the characterization of EVs within the SSC niche. PND7 testis EVs exhibited hallmark cup-shaped morphologies (Figure 1A) of sizes 50–300 nm, with a major peak at around 150 nm (Figure 1B). These EVs expressed EV-specific markers CD9, CD81, and CD63, while lacking organelle markers calnexin and golgin 97 (Figure 1C). Small EVs represent the major population in the neonatal mouse testis, consistent with adult testis EVs (Choy et al., 2022; Yun et al., 2019).

To probe for the potential functions of PND7 testis EVs on SSCs, we added EVs to primary spermatogonial culture (Kanatsu-Shinohara et al., 2003), a heterogeneous culture consisting of different spermatogonial subpopulations including SSCs and committed progenitors (Figure 1D). Flow cytometry confirmed time-dependent uptake of PKH67-labeled EVs, with 9%, 19%, and 80% of primary spermatogonia positive for PKH67 signals after testis EV

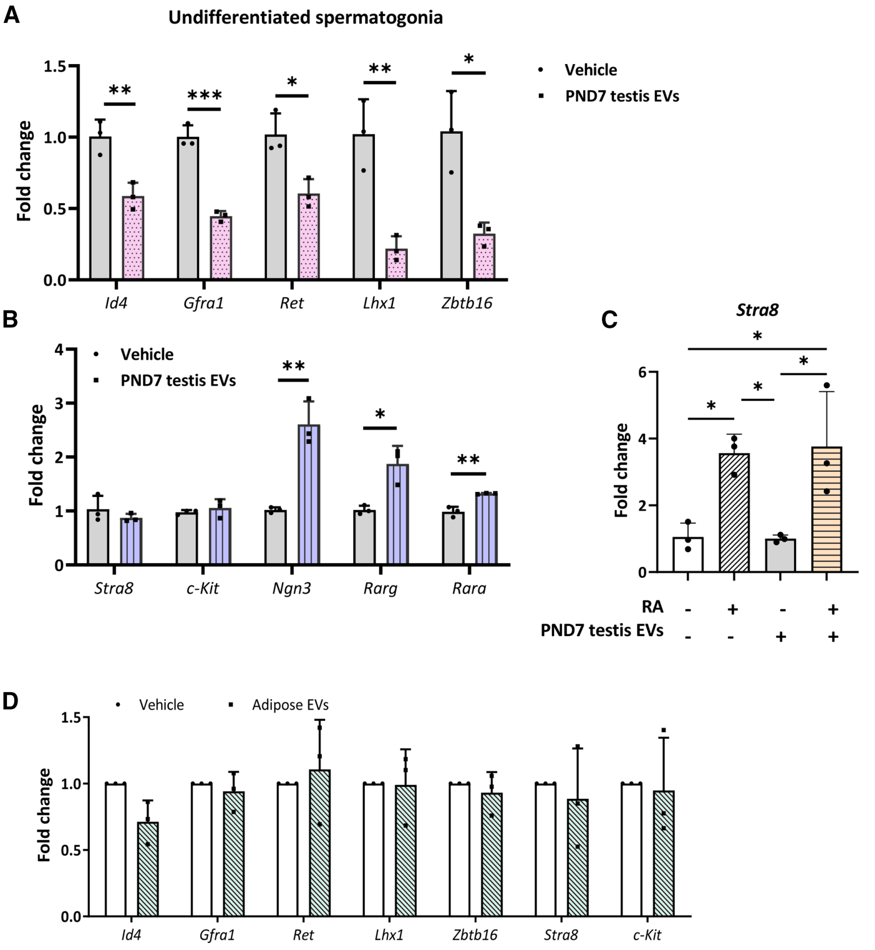

Figure 2. EVs from neonatal testis guide spermatogonia into a transit-amplifying state

Real-time PCR results show that compared to the vehicle-treated group, treatment of PND7 testis EVs (1 μg/mL) (A) significantly decreased the expression of undifferentiated markers *Id4*, *Gfra1*, *Ret*, *Lhx1*, and *Zbtb16* in primary spermatogonia, (B) but had no effect on the expression of the differentiation markers *Stra8* and *c-Kit* (see also Figure S1).

(B) The expression of *Ngn3*, *Rarg*, and *Rara* increases in primary spermatogonial culture after exposure to PND7 testis EVs.

(C) PND7-testis-EV-treated spermatogonia showed a comparable induction of *Stra8* after RA treatment as compared to those without testis EV treatment.

(D) Real-time PCR results showing the expression levels of undifferentiated and differentiating spermatogonia markers in primary spermatogonia after exposure to EVs isolated from adipose tissues. *n* = 3 independent experiments.

treatment for 3 h, 6 h, and 24 h, respectively, suggesting an efficient uptake after 24-h exposure (Figure S1). Exposure of primary spermatogonia to testis EVs for 3 days dose-dependently reduced the expression of undifferentiated spermatogonial markers, with significant effects at 1 μg/mL of testis EVs, a concentration used in subsequent experiments (Figure S1). A 5-day treatment did not alter the morphology but increased the number of SSC clumps (Figure 1E). Since the primary spermatogonial culture is heterogeneous with SSCs representing only 10% of the cells, we have further counted the cell number. Intriguingly, PND7 testis EV treatment significantly increased the total cell number (1.93-fold) and clump number (1.58-fold) of the heterogeneous spermatogonial culture (Figure 1F), suggesting that the PND7 testis EVs enhanced the spermatogonial proliferation or survival and more cell number present per clumps (Figure 1G). Consistent with the increase in proliferation, PND7 testis EV increased DNA replication of spermatogonia as revealed by an increased number of cells incorporating BrdU (Figure 1H), without inducing apoptosis or necrosis (Figure S2). Taken together, these results suggest that PND7 testis EVs promote spermatogonial proliferation or survival.

Testis EVs guide spermatogonia to the transit-amplifying state

Next, we assessed the effect of testis EVs on the expression of self-renewal and differentiation markers of SSCs. We found that PND7 testis EVs inhibited the expression levels of SSC marker *Id4*; A_s spermatogonia marker *Gfra1*; and pan-undifferentiated spermatogonia markers *Zbtb16*, ret receptor tyrosine kinase (*Ret*), and LIM homeobox 1 (*Lhx1*) in the cultured spermatogonia (Figure 2A). Intriguingly, while differentiation markers *Stra8* and *c-Kit* were comparable, we observed a significant increase in the expression of *Ngn3*, retinoic acid (RA) receptors alpha (*Rara*) and gamma (*Rarg*), and markers of undifferentiated spermatogonia primed for differentiation (Figure 2B) (Gely-Pernot et al., 2015; Yoshida et al., 2004). Unexpectedly, despite the elevated *Rara* and *Rarg*, the PND7 testis EV-treated spermatogonia demonstrated a normal Stra8 response to RA (Figure 2C). Besides, adipose tissue

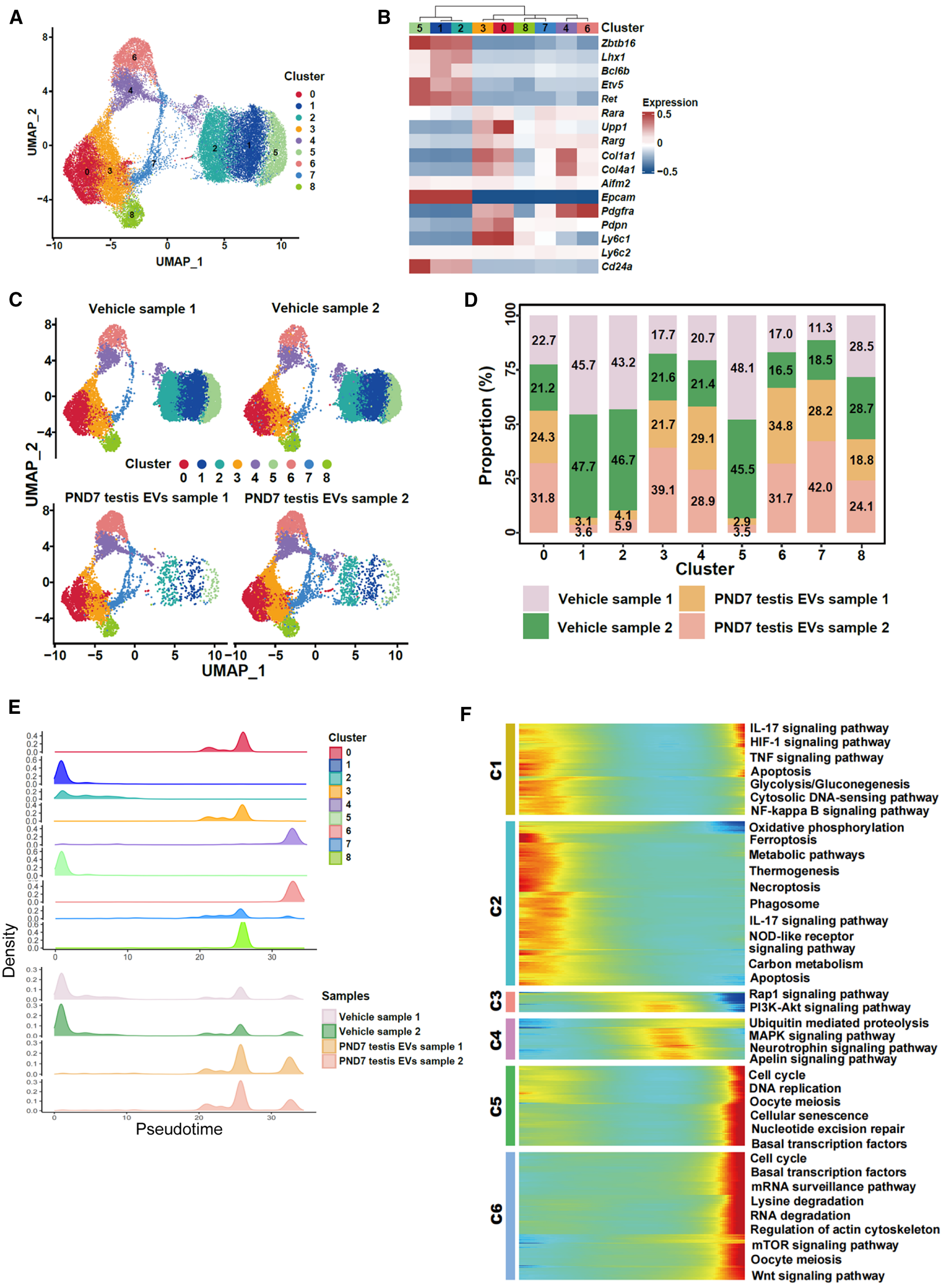

(legend on next page)

EVs did not alter spermatogonial marker expression (Figure 2D), indicating that the observed effects of the PND7 testis EVs on primary spermatogonial culture are specific.

In corroboration with this, single-cell RNA sequencing analysis revealed that PND7 testis EV treatment significantly increased the progenitor population. Unsupervised clustering generated nine populations in primary spermatogonial culture (Figure 3A). Based on the known markers of various spermatogonial subpopulations (Figure 3B), nine clusters were classified into SSCs (clusters 1, 2, and 5) and progenitor spermatogonia (clusters 0, 3, 4, 6, and and 7). The UMAP analyses revealed that the PND7-testis-EV-treated spermatogonial cultures exhibited a marked shift from stem cells to progenitors compared to the control group (Figure 3C). The proportion of SSCs decreased from >40% in vehicle group (cluster 1: 46.7% ± 1.4%; cluster 2: 45.0% ± 2.5%; and cluster 5: 46.8% ± 1.8%) to <5% in the PND7-testis-EV-treated group (cluster 1: 3.4% ± 0.4%; cluster 2: 5.0% ± 1.3%; and cluster 5: 3.2% ± 0.4%), whereas a 1.4- and 2-fold increase in clusters 3 and 4 and 6 and 7, respectively (Figure 3D). Pseudotime analysis revealed six gene clusters that aligned well with spermatogonial development *in vivo*, including glial-cell-line-derived neurotrophic factor (GDNF) signaling (PI3K/Akt signaling) in SSC, the activation of cell cycle, and enhanced protein turnover (ubiquitin-mediated proteolysis) in progenitors (Figures 3E and 3F). Similarly, we observed PND7 testis EVs shift SSCs to progenitors (Figure 3E). This was supported by altered gene expression in key pathways: differential gene expression analysis within each cell cluster revealed significant alterations in key signaling pathways involved in SSC self-renewal (PI3K/Akt, mitogen-activated protein kinase [MAPK], and Ras signaling pathways) and progenitor differentiation (cell adhesion and extracellular matrix [ECM]-receptor interaction and Rap1 and HIF1 signaling pathways) (Figure S3), supporting the role of EVs in regulating the gene expression of recipient cells, ensued by the shift in cell status.

These results suggest that the PND7 testis EV treatment guides the spermatogonia into a transit-amplifying state.

Repertoires of small RNAs and proteins in the PND7 testis EVs

Next, we investigated the cargoes of the PND7 testis EVs to identify potential candidates in mediating the effect on SSCs. Small RNA sequencing identified 2,131 small RNAs, representing the dominant RNA species in testis EVs (Choy et al., 2022), in PND7 testis EVs. Thirty-five percent of the clean reads were annotated as rRNA, tRNA, small nucleolar RNA (snoRNA), small nuclear RNA (snRNA), Rfam small non-coding RNA (sncRNA), and microRNAs (miRNAs), including precursor, mature and hairpin, whereas the remainder mapping to other genomic loci (Figure S4A). Notably, miRNAs represented 6.06% of the reads and were the predominant small RNA subtypes. Most of the 50 most abundant miRNAs in testis EVs have been demonstrated to mediate the maintenance and differentiation of stem cells (Table S1). For example, miR125b, miR-21, miR-378, and miR-652 serve as signals for maintaining the self-renewal of snail-induced stem cells, mesenchymal stem cells, and colon cancer stem cells, respectively (Deng et al., 2013; Yang et al., 2020; Yu et al., 2015); miR-34, miR-451, and let-7 families promote the differentiation of neural stem cells and cancer stem cells (Aranha et al., 2011; Bitarte et al., 2011; Büssing et al., 2008; Peng et al., 2017). We have subsequently selected 10 highly expressed miRNAs as the candidate for subsequent investigations.

To identify protein cargoes, our proteomic analysis identified 943 proteins in the PND7 testis EVs (Figure S4B; Table S2). These proteins represent the core proteome of the EVs in the neonatal mouse testis. Among these proteins, 88 were reported to be exosome protein markers (http://www.exocarta.org/) (Table S3), validating the successful EV isolation. Then, we mapped the proteins identified in the PND7 testis EVs to genes and conducted gene ontology (GO) analysis. The GO cellular component analysis showed that in line with the affinity column purification of membranous vesicles, proteins found in the PND7 testis EVs were enriched in the membrane and integral components of the membrane (Figure S4C). GO biological process analysis revealed proteins involved in cell adhesion, cell migration, and positive regulation of cell proliferation that were critical processes for the establishment of

Figure 3. Single-cell analysis of primary spermatogonial culture upon neonatal testis EVs treatment

(A) UMAP plots of a merged dataset containing vehicle and PND7-testis-EV-treated spermatogonia cultures, revealing nine clusters.

(B) Heatmap displaying the expression of marker genes for stem cells, progenitors, and fibroblasts, collapsed from each cluster, with mRNA levels represented on a *Z* score scale.

(C) UMAP plots of nine cell clusters within each sample.

(D) Proportions of vehicle and PND7-testis-EV-treated spermatogonia across the nine clusters.

(E) Pseudotime analysis of spermatogonia, illustrated using density maps to show the distribution of cells from different populations and samples in pseudotime.

(F) Differentially expressed genes along pseudotime were hierarchically clustered into six profiles, with representative gene functions and pathways highlighted for each profile. $n = 2$ independent samples (see also Figure S3).

spermatogonia and Sertoli cells before the first wave of spermatogenesis (Figure S4D) (Manku and Culty, 2015). GO molecular function analysis identified protein binding as the major function of proteins in the PND7 testis EVs. Notably, genes encoding the proteins identified in the PND7 testis EVs were known to regulate the SSCs or other tissue stem cells (Figure S4E; Table S4). Together, our results have identified the small RNA and protein signatures of the PND7 testis EVs that may regulate the establishment and function of the SSC niche.

EVs from spermatogonia and Sertoli cell lines mimic the effects of the PND7 testis EVs

In the PND7 testis, the undifferentiated and differentiating spermatogonia undergo the first wave of spermatogenesis, whereas Sertoli cells undergo proliferation. This event is crucial for the Sertoli-germ cell contact and facilitates the formation of the SSC niche during the first round of spermatogenesis. To decipher the donor cells that contribute to the effect of the PND7 testis EVs, we have isolated the EVs from immortalized cell line models: C18-4 undifferentiated spermatogonial cell line, the GC1-spg differentiating spermatogonia cell line, and TM4 Sertoli cell line cultures. Although these immortalized cell lines are incomplete representations of given cell types in SSC niche, this approach overcomes the technical difficulties of obtaining sufficient pure primary cells for EV collections. Since the primary spermatocyte first appears at PND14, GC2-spd spermatocyte cell line was used as a control. The EVs from the four cell lines showed cup-shaped morphology with a size of 100–250 nm and a peak of size distribution at around 150 nm (Figures 4A, 4B, and S5).

Next, we test the biological activities of the cell-line-derived EVs by adding cell-line-derived EVs to the primary spermatogonial culture. EVs isolated from C18-4 undifferentiated spermatogonial cell line (C18-4 EVs) and the TM4 Sertoli cell line (TM4 EVs) significantly inhibited the expression of undifferentiated spermatogonia markers, including *Id4*, *Gfra1*, *Zbtb16*, *Ret*, and *Lhx1*, while the markers for differentiating spermatogonia, *Stra8* and *c-Kit*, remained unchanged (Figures 4C and 4D). These results highly resemble those observed for the PND7 testis EV treatment of spermatogonial culture. Interestingly, the expression levels of *Ngn3* increased in the spermatogonia exposed to the C18-4 EVs but not the TM4 EVs. Moreover, the levels of *Rarg* and *Rara*, which both were upregulated by the PND7 testis EVs, were not altered by the C18-4 or TM4 EVs (Figures 4C and 4D). The EVs isolated from GC1-spg (GC1-spg EVs) and GC2-spd cell lines (GC2-spd EVs) did not pose any significant effect on the expression profiles of spermatogonia markers (Figure S5). In line with the expression of markers, BrdU assay demonstrated that C18-4 and TM4 EVs significantly increased the percentage of BrdU-incorporating cells and cell numbers in the primary spermatogonial culture (Figures 4E and 4F). These results suggest that the EVs originating from spermatogonia and Sertoli cell lines mimic the effects of the PND7 testis EVs on spermatogonial proliferation and differentiation.

Involvement of let-7b/c miRNA cargoes in mediating the effect of the PND7 testis EVs

Our previous experiments showed that the PND7 testis EVs promoted spermatogonial proliferation and engaged spermatogonia at the transit-amplifying state. Since the PND7 testis EVs carried miRNA cargoes, some of which had been reported to regulate the maintenance of stem cells, we postulated that these miRNA cargoes were transferred to the spermatogonia to regulate their proliferation and fate decision.

To test this hypothesis, we validated the candidate miRNA expression of the PND7 testis EVs (Table S1). MiR-125b, miR-378a, miR-652, let-7b, and let-7c were significantly enriched in the PND7 testis EVs. In contrast, miR-34a and let-7f were substantially lower, while miR-21, miR-451a, and let-7a were comparable to total testis RNA (Figure 5A). To examine if the miRNA cargoes carried by the PND7 testis EVs were transferred to spermatogonia, we profiled the miRNAs in primary spermatogonia after the treatment with the PND7 testis EVs. Indeed, the expression levels of miR-125b, miR-378, let-7b, and let-7c were significantly increased in the EV-treated spermatogonia (Figure 5B), suggesting that these miRNAs were transferred to spermatogonia. In addition, let-7b and let-7c were highly enriched in the C18-4 EVs and TM4 EVs, representing a 2.4-fold and 2-fold enrichment compared to that of the total RNA extracted from mouse testes (Figures 5C and 5D). Consistent with the lack of response in the EVs isolated from the GC1-spg and GC2-spd lines, let-7b and let-7c were significantly lower in the GC1-spg EVs and GC2-spd EVs (Figures 5C and 5D).

To further examine the involvement of let-7b/c in mediating the effect of testis EVs, we attempted to establish loss-of-function models targeting let-7b and let-7c. Due to the highly conserved and extremely short seed sequence of the let-7 family, we used single guide RNAs (sgRNAs) targeting the Dicer processing sites of let-7b and let-7c precursors instead of conventional sgRNA on seed sequence or inhibitors/antagomirs. Using lentiviral CRISPR/Cas9, we mutated the precursors in C18-4 and TM4 cell lines to perturb let-7b and let-7c biogenesis. This reduced mature miRNA levels to 0.4- to 0.6-fold (Figure S6), an outcome mimicking the knockdown condition. Thus, we referred to these mutant cell lines as let-7b or let-7c knockdown (let-7b KD or let-7c KD).

We found that compared to the vehicle-treated group, the EVs from wild-type (WT) C18-4 and TM4 cell lines

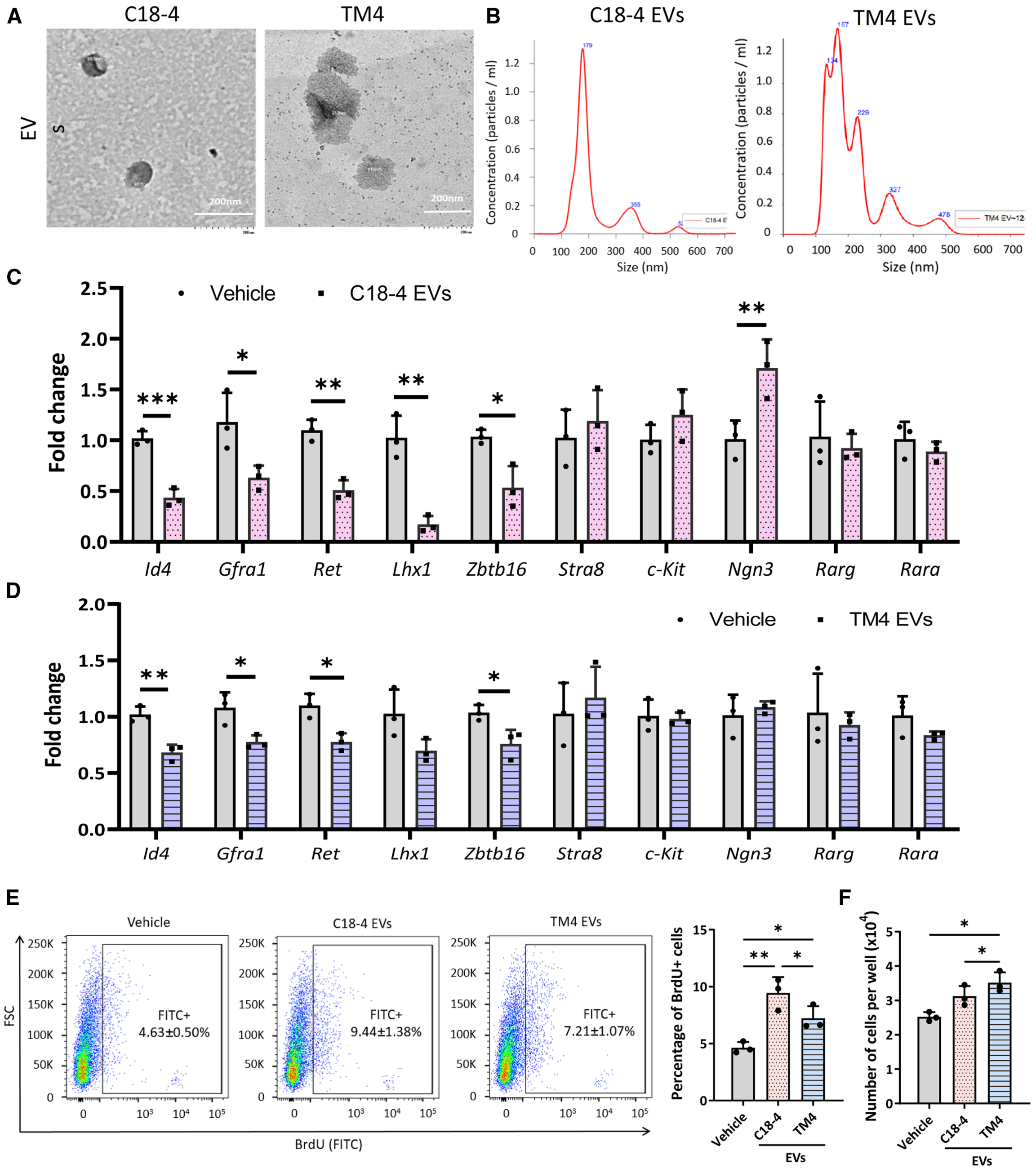

Figure 4. EVs from spermatogonia and Sertoli cells lines mimic the effects of PND7 testis EVs on primary spermatogonial culture

(A) Representative transmission electron microscopy image of EVs isolated from C18-4 and TM4 cell lines by ultracentrifugation. Scale bars, 200 nm.

(B) Size distribution of cell-line-derived EVs isolated as in (A) determined by nanoparticle tracking analysis. Real-time PCR results showing the expression of SSC marker *Id4*; A_s spermatogonia marker *Gfra1*; pan-undifferentiated spermatogonia markers *Zbtb16*, *Ret*, and *Lhx1*; spermatogonial differentiation markers *Stra8* and *c-Kit*; and progenitor markers *Ngn3*, *Rara*, and *Rarg* in primary spermatogonia after treatment with EVs isolated from C18-4 (C) and TM4 (D) cell lines (1 μg/mL). (E) Representative flow cytometry analysis of BrdU uptake in primary spermatogonia exposed to C18-4 and TM4 EVs (1 μg/ml), and (F) total cell number of primary spermatogonia exposed to C18-4 and TM4 EVs. *n* = 3 independent experiments (see also Figure S5).

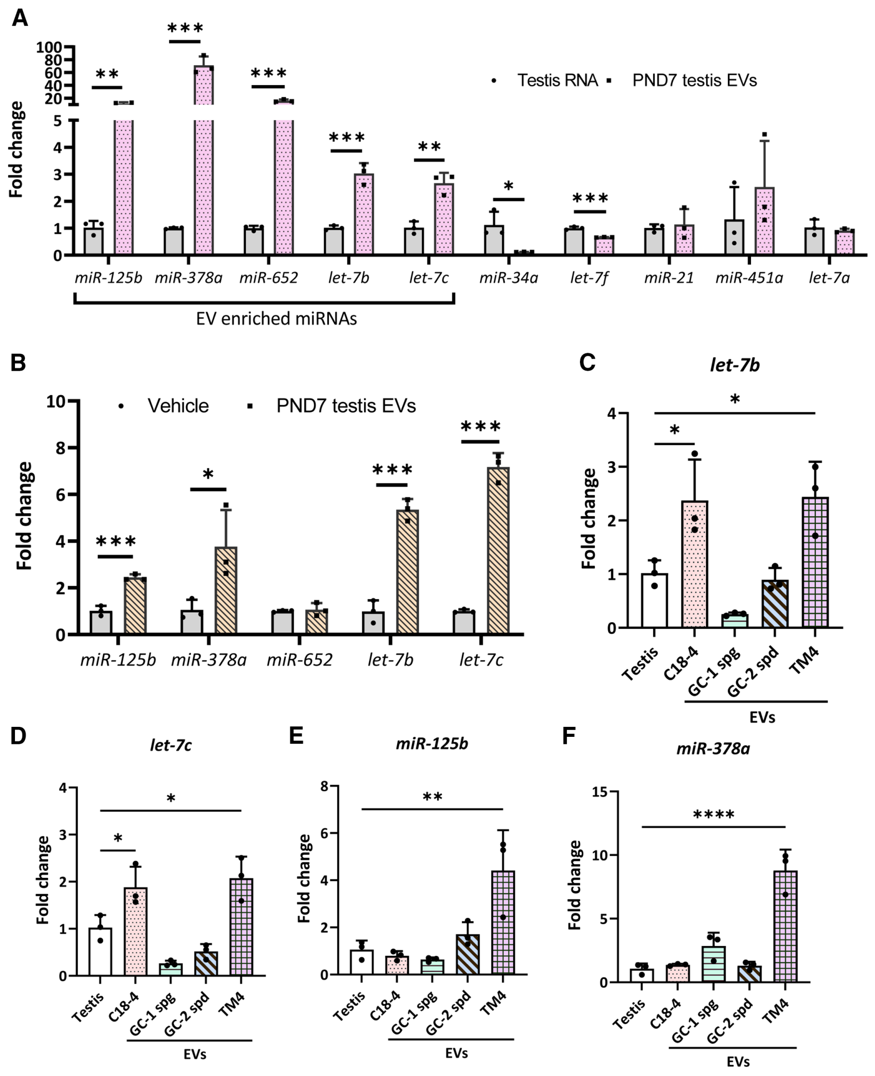

Figure 5. EVs from neonatal testis convey EV-enriched miRNAs to primary spermatogonia

Real-time PCR results showing the expression of indicated miRNAs in (A) PND7 testis EVs and (C–F) EVs isolated from C18-4, TM4, GC1-spg, and GC2-spd cell lines. Total testis RNA (testis RNA or testis) was used as a control.

(B) The expression of indicated miRNAs in primary spermatogonial treated with PND7 testis EVs (1 μg/mL) or same volume of PBS in the medium (vehicle). Data were normalized to snoU6 RNA levels.

could significantly inhibit the expression of undifferentiated markers *Id4*, *Gfra1*, *Zbtb16*, and *Ret*, and C18-4 EVs also induce the expression of *Ngn3* in primary spermatogonia. However, the effects of EVs were partially inhibited in let-7b or let-7c KD C18-4 and TM4 cell lines (Figures 6A and 6B). These results suggest that let-7b and let-7c are the potential cargoes of the PND7 testis EVs being conveyed to spermatogonia and mediating the effect of EVs on spermatogonial proliferation and differentiation.

DISCUSSION

In the present study, we have shown that PND7 testis EVs obtained from a physiological source after the establishment of spermatogonial populations in the niche significantly promote spermatogonial proliferation and prime spermatogonia to a transit-amplifying state, strongly suggesting a role in the intercellular communication of SSCs and other spermatogonial subpopulation after the establishment of the niche. However, it should be noted that the testis EVs analyzed in this study represent a heterogeneous mixture derived from multiple cell types present in PND7 testis. Therefore, the observed effects on SSCs represent the combinatorial effects of EVs secreted by all cell types in the testis, regardless of the spatial effect of these cells within and outside the niche. While we traced the origin of biologically active EVs from spermatogonia and Sertoli cells using established cell lines, isolating EVs from primary testicular cells remains technically challenging. Therefore, the results should be interpreted with caution that the C18-4, GC1-spg, and TM4 cell lines, while useful proxies, may not fully replicate the native functions of spermatogonial subpopulations or Sertoli cells in the SSC niche.

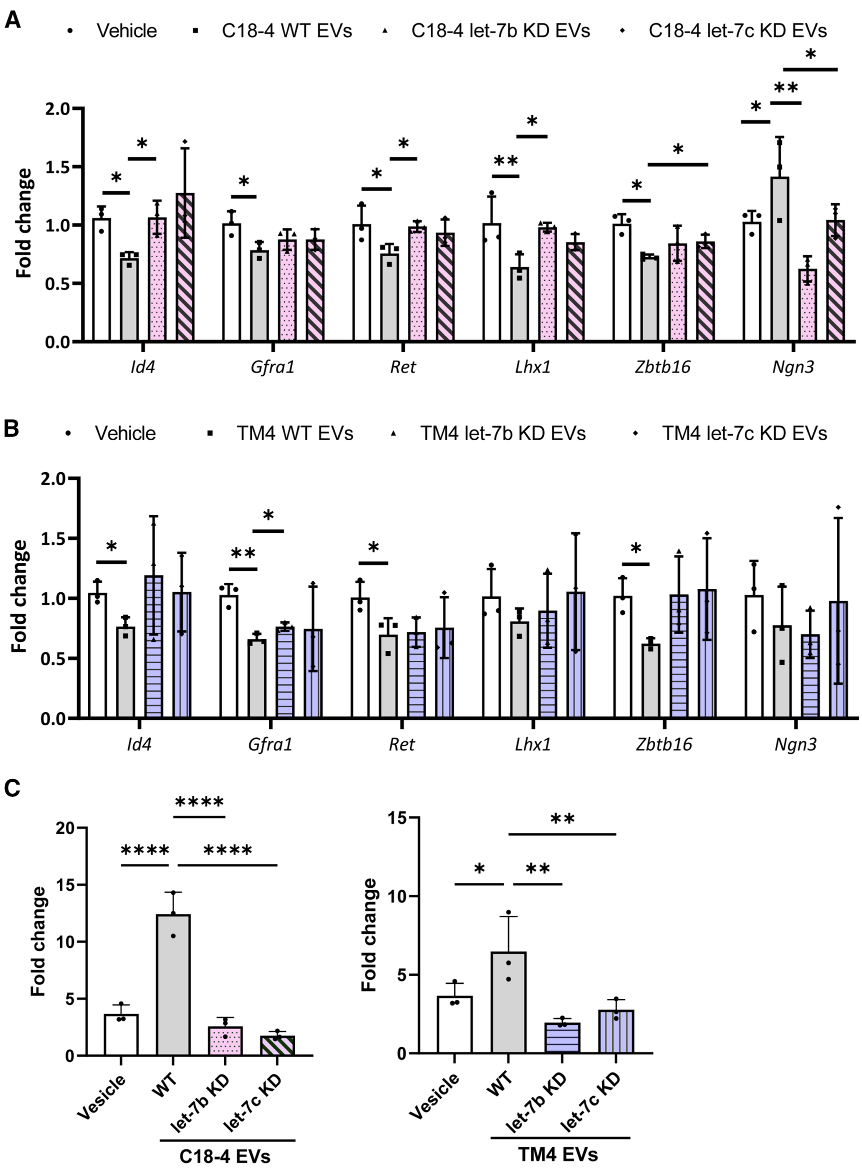

Figure 6. Knockdown of let-7b/7c in donor cells perturbs the effects of EVs on primary spermatogonial culture

Real-time PCR results showing the expression of *Id4*, *Gfra1*, *Zbtb16*, *Ret*, *Lhx1*, and *Ngn3* in WT or KD C18-4 lines (A) or TM4 lines (B).

(C) Percentages of BrdU uptake in primary spermatogonia exposed to WT or KD C18-4 lines or TM4 lines. *n* = 3 independent experiments (see also Figure S6).

The intracytoplasmic bridges interconnect mitotic sister spermatogonia. Previous studies have reported the competition among GFRα1-positive spermatogonia for mitogens that determine the homeostasis of SSC (Kitadate et al., 2019). The Thy1$^+$ EVs isolated from Thy1$^+$ spermatogonia inhibit spermatogonia proliferation and decrease the number of SSC clumps, whereas spermatogonia-derived Thy1$^-$ EVs have no effect, and these spermatogonia-derived EVs do not alter the apoptosis of primary spermatogonia (Lin et al., 2020). Consistent with these findings, our results showed that the PND7 testis EVs posed negligible effects on the apoptosis of spermatogonia. However, in contrast to the Thy1$^+$ EVs, testis EVs increased the proliferation of primary spermatogonia. It is worth noting that the testis EVs are produced by various cell types in the testis. While our study could not exclude the involvement of Thy1$^+$ EVs, our results together with the previous study suggested a combinatorial effect of EVs from spermatogonia and other somatic cells in the SSC niche. More importantly, the present study suggested that both neighboring spermatogonia and Sertoli cells are the pivotal components in the SSC niche via EV-mediated intercellular communication.

In the SSC niche, different spermatogonial subpopulations are exposed to potential intercellular communication from the neighboring undifferentiated and differentiated spermatogonia, Sertoli cells, Leydig cells, and other somatic cells such as endothelial cells and macrophages. In this study, the

EVs from C18-4 undifferentiated spermatogonia and TM4 Sertoli cell lines could partially recapitulate the effects of testis EVs on spermatogonial cultures, while those secreted from GC1-spg premeiotic differentiated spermatogonia are dispensable, implying that the EV-mediated intercellular communication is more restricted to the primitive spermatogonial colonies and their microenvironment. It should be noted that the SSC niche also involves other untested somatic cells, e.g., Leydig cells, macrophages, endothelial cells, and peritubular myoid cells. To date, the contribution of endothelial cells and peritubular myoid cells to testis EVs has not been reported. Interestingly, both Leydig cells and macrophages have been postulated to contribute to the testis EVs (Choy et al., 2022), which may contribute to the biological effects of testis EVs, for example, the upregulation of *Rara* and *Rarg* (Figure 2B), that are not observed from the C18-4 EVs and the TM4 EVs. Further studies are required to investigate the role of EVs from these other niche cells on SSCs.

An important finding of our study is the identification of let-7b and let-7c as the functional miRNA cargo candidates of testis EVs in regulating spermatogonial proliferation and transit amplification. Let-7b and let-7c KD significantly suppressed the effects of the C18-4 or TM4-derived EVs on spermatogonia, despite incomplete knockdown. We postulate that let-7b and let-7c hairpin precursors in the EVs produced by mutant lines could still be functional upon uptake. Nonetheless, our results showed that let-7b/7c were two miRNA cargoes that mediated the effect of testis EVs.

EVs are promising tools for genetic engineering and therapy due to their role in intercellular communication and cargo delivery. Our study shows that efficiently taken up by 80% of spermatogonia after the 24-h treatment, testis EVs offer a more effective vehicle for genetic engineering than the traditional transfection or transduction techniques. Testis EVs also induce SSC and progenitors proliferation, aiding the *in vitro* propagation of these spermatogonial populations. In addition, testis EVs may be used for the treatment of non-obstructive azoospermia, which imputes the defects of spermatogenesis by targeting spermatogonia.

Taken together, our study has revealed, for the first time, that neonatal mouse testis EVs promote spermatogonial proliferation and retain the differentiation potential of primary spermatogonial cultures, providing solid evidence for the participation of testis EVs in the intercellular communication within the SSC niche. EVs from Sertoli and undifferentiated spermatogonia cell lines partially recapitulate the effects of testis EVs via the miRNA cargoes let-7b/7c, suggesting that the EV-mediated intercellular communication involves the neighboring spermatogonia and their microenvironment. Our study has shed light on the EV-mediated regulation of the SSC niche and fate decision.

METHODS

Animals

PND7 and 8-week-old male C57BL/6 mice were purchased from the laboratory animal service center (LASEC) of the Chinese University of Hong Kong. All procedures for the animal experiments were approved by the Animal Research Ethics Committee of the Chinese University of Hong Kong (20/081/ECS-5-C).

Isolation and culture of primary spermatogonia

Primary spermatogonial culture was established from three PND6 male mice as previously described (Fok et al., 2017; Kanatsu-Shinohara et al., 2003; Nagano et al., 2003). Briefly, the decapsulated testes were dissociated with scissors and placed in solution I (1 mg/mL Collagenase Type IV, Sigma) for 10 min at 37°C. Tissues and cells were collected by brief centrifuge at 300×g for 15 s, followed by incubation in solution II (0.25% Trypsin, Gibco; 0.5 mL 5 mg/mL DNase, Thermo Fisher) for 10 min at 37°C, then stopped by the addition of 10% fetal bovine serum (FBS). The cell suspension was filtered through a 40-μm strainer, and cells were collected by centrifugation at 300×g for 5 min.

Spermatogonia were isolated by magnetic activated cell sorting (MACS) against Thy1; 1×10^7 cells resuspended in 200 μL of DMEM medium supplemented with 2% FBS were incubated with 20 μL of biotinylated Thy1.2 CD90.2 primary antibody (BD IMag, 551518) on ice for 15 min on a slow-rocking platform. Cells were washed with PBS and resuspended with 500 μL of DMEM medium supplemented with 2% FBS before being loaded into the pre-calibrated MS column (Miltenyi Biotec, 130-042-201) to capture $Thy1^+$ cells. Columns were washed twice with 500 μL of DMEM medium supplemented with 2% FBS, and the $Thy1^+$ cells were eluted with 1 mL of DMEM medium supplemented with 2% FBS. Then, the $Thy1^+$ cells were cultured on the STO feeder cells, as previously described (Kubota et al., 2004), in culture medium (StemPro 34 medium with supplement [Invitrogen]; 0.1 mg/mL Fe-saturated transferrin [Sigma]; 5 mg/mL bovine serum albumin [Sigma]; 6 mg/mL D-(L)-glucose [Sigma]; 30 nM sodium selenite [Sigma]; 5×10^{-5}M p-mercaptoethanol [Sigma]; 60 mM putrescine [Sigma]; 25 μg/mL insulin [Sigma]; 30 μg/mL pyruvic acid [Sigma]; 1 μL/mL DL-lactic acid [Sigma]; minimal essential medium [MEM] vitamin solution [Invitrogen]; MEM nonessential amino acid solution [Invitrogen]; 10^{-4} M ascorbic acid [Sigma]; 10 μg/mL d-biotin [USB corp]; 30 ng/mL β-estradiol [Sigma]; 60 ng/mL progesterone [Sigma]; 2 mM L-glutamine [Life Technologies]; 1% fetal bovine serum [Gibco]; 10 ng/mL

human basic fibroblast growth factor [hFGF] [Sigma]; 1% P/S and 20 ng/mL recombinant human glial-cell-line-derived neurotrophic factor (GDNF) [R&D Systems]) and maintained at 37°C. The culture medium was changed every 2–3 days and the stem cells proliferated to form tight clumps that were loosely attached to the STO feeders after 6–7 days in culture.

Cell lines culture

The cell lines used in this study were the C18-4 mouse SSC line, GC1-spg mouse differentiating spermatogonia cell line, GC-2 spd mouse spermatocyte cell line, and TM4 mouse Sertoli cell line. C18-4 cells were cultured in DMEM medium supplemented with 10% FBS, 1% L-glutamine, 1 mM sodium pyruvate, and 1× nonessential amino acids at 35°C. GC1-spg cells and GC-2 spd were maintained in DMEM medium containing 10% FBS and 1% penicillin and streptomycin at 37°C. TM4 cells were cultured in DMEM/F12 medium containing 10% FBS and 1% penicillin streptomycin at 37°C.

Isolation of EVs from the testis tissue, testicular cell lines, and adipose tissue

The PND7 stage was strategically selected as spermatogonial subpopulations were established and represent the dominant germ cells in the testis at this developmental stage, simplifying the EV source. EVs were isolated from 10 PND7 C57/BL6 male mice as described (Choy et al., 2022). Testes were decapsulated and release tubules using forceps and then digested by 1 mL Accumax (Thermo Fisher) per 20 mg tissue at room temperature for 1 h. Tissues were gently pipetted up and down 20 times to maximize the cell yield followed by filtering through a 40-μM strainer. The digestion was stopped by the addition of one volume of PBS, and the cells were pelleted by centrifugation at 300×g for 10 min. EVs were isolated from the supernatant using exoEasy Maxi Kit (Qiagen, 76064) according to the manufacturer's instructions, then concentrated by ultracentrifugation (Hitachi CS-150GXII Micro UlTRAcentrifuge) at 100,000×g for 90 min at 4°C. The EV pellet was resuspended in 20 μL PBS and stored at −80°C for further analysis.

EVs were isolated from cell lines grown to 95% confluency in ten 100-mm cell culture dishes (Genetimes, 20100) with 10 mL conditioned culture medium (CCM) supplementary with exosome-depleted FBS (exosomes were removed by ultracentrifugation at 150,000×g for 18 h at 4°C) in each dish and incubated for 48 h. The EV-containing CCM was collected, and the EVs were isolated by differential ultracentrifugation method. Briefly, CCM was sequentially centrifuged: 300×g 10 min (remove cells); 3,000×g 20 min (remove debris); and 10,000×g 30 min (remove large vesicles, Beckman Avanti J-E Centrifuge) at 4°C, followed by ultracentrifuge (Beckman Optima XPN-100 Ultra-High Speed Refrigerated Centrifuge) at 100,000×g 4°C for 90 min to pellet the small vesicles. The pellets were washed by PBS with ultracentrifuge (Hitachi CS150FNX Ultra-Microcentrifuge) at 100,000×g 4°C for 90 min. The EV pellet was resuspended in 20 μL PBS and stored at −80°C for further analysis.

Adipose-derived EVs were isolated from mouse visceral adipose tissue, as previously reported (Wei et al., 2020). Tissue samples were washed in PBS, minced into ~2 mm^3 fragments, and incubated in serum-free medium at 37°C for 24 h. The resulting conditioned medium was collected, and EVs were isolated using differential ultracentrifugation, as previously described.

Transmission electron microscope

EV morphology was observed using TEM. Briefly, EVs were resuspended in 20 μL PBS and fixed with 2% paraformaldehyde until use; 10 μL EVs were added onto the formvar grid (200 mesh) for 30–60 min, and excess fluid was removed with filter paper. EVs were fixed with 1% glutaraldehyde for 10 min, followed by negative staining with 2% uranyl acetate for 2 min, and three images were captured at different fields using a Hitachi H-7700 transmission electron microscope (TEM).

Nanoparticle tracking analysis

EV concentration was measured by NanoSight LM14C (Malvern). Briefly, EV samples were diluted with filtered PBS to ~10^7–10^9 particles/mL, which allows the viewing of approximately 20–100 particles in each field. Three 30-s videos were captured at three different fields. The concentration was calculated by the NanoSight software using the Stokes-Einstein equation.

Western blot

Protein from EVs, cells, and tissues was extracted by RIPA lysis buffer with protease and phosphatase inhibitor. A total of 40 μg of protein was denatured followed by loading on 10% SDS-PAGE gel for electrophoresis and transferring onto the polyvinylidene fluoride (PVDF) membranes (Sigma). The membrane was then blocked with 5% non-fat milk in Tris-buffered saline with Tween 20 (TBST) at room temperature for 1 h and immunoblotted with primary antibodies CD 63 (Santa Cruz; sc-365604, 1:1,000), CD 81 (Santa Cruz; sc-166029, 1:1,000), CD9 (Santa Cruz; sc-13118, 1:1,000), calnexin (Immunoway; YT0613, 1:1,000), golgin 97 (Santa Cruz; sc-59820, 1:1,000), and β-tubulin (Cell Signaling; 2146, 1:2,000) overnight at 4°C. After incubation with HRP-conjugated secondary antibody, immunoreactions were detected by Amersham ECL Advance Western Blotting Detection Kit (GE Healthcare, RPN2135) and Super X-film (Fuji Medical).

Uptake of PKH67-labeled testis EVs by primary spermatogonia

Purified EVs were labeled using the PKH67 Green Fluorescent Cell Linker Mini Kit (Sigma-Aldrich) according to the manufacturer's instructions. Briefly, 100 μL of EVs resuspended in PBS was mixed with 1 mL Diluent C and 6 μL PKH67 dye, followed by 5-min incubation. Excess dye was quenched by adding 2 mL of 0.5% BSA/PBS, and 20 mL PBS was added to prevent vesicle aggregation during ultracentrifugation. Labeled EVs were washed via ultracentrifugation and resuspended in 50 μL PBS. Primary spermatogonia were co-cultured with labeled EVs for 3, 6, and 24 h, and EV uptake was analyzed by flow cytometry.

EV treatments

One microgram of EVs or an equal volume of PBS was added into 1 mL of fresh complete culture medium supplemented with 20 ng GDNF and 10 ng hFGF. After mixing, the mixture was filtered with 0.22 μm Millex-GP filters (Millipore Sigma, SLGP033RB). On day 1 post-passaging, the filtered culture medium was added to the primary spermatogonial culture, and the cells were incubated at 37°C in an incubator with 5% CO_2 for 3 days.

BrdU incorporation assay

The culture medium was removed and replaced with fresh medium containing 10 μM BrdU for 12 h at 37°C. After incubation, spermatogonial clumps were collected by flushing from the feeder cells as described (Fok et al., 2017). The clumps were collected and trypsinized into a single-cell suspension, followed by fixing with dropwisely adding 5 mL ice-cold 70% ethanol and incubated at −20°C for 2h and permeating with 2 M HCl/0.5% Triton X-100 at room temperature for 30 min. After washing twice with 1% BSA in PBS, cells were incubated with the anti-BrdU antibody (Abcam, ab6326, 1:100) and anti-DDX4/MVH antibody (Abcam, ab13840, 1:100) at room temperature for 1 h. Then, the samples were stained with Alexa Fluor 488 anti-rat immunoglobulin G IgG) (H + L) (Invitrogen, A21208, 1:1,000) and Alex Fluor 647 anti-rabbit IgG (H + L) (Invitrogen, A31573, 1:1,000) at room temperature for 1 h and finally resuspended in the solution of 1% BSA in PBS to 10^6–10^7 cells/mL and analyzed by flow cytometry (BD LSRFortessa Cell Analyzer). The results were analyzed by FlowJo v.10.

Clump formation assay

Primary cultured spermatogonia were seeded at 1×10^4 cells/cm^2 in 48-well culture dishes (0.96 cm^2, $\sim 10^4$ cells/well). The culture was treated with 1 μg/mL testis EVs for 6–7 days. Clumps were counted manually on the 6th day under an inverted fluorescence microscope. The clump is defined as a group of at least six cells with globular three-dimensional structures attached to the feeder cells (Yeh et al., 2007).

Reverse transcription and quantitative real-time PCR

Prior to RNA extraction, EVs samples were treated with 0.05 μg/μL Proteinase K (Qiagen, 19131) at 37°C for 10 min, 5 mM PMSF (Sigma-Aldrich, PND7627) at room temperature for 10 min, and 0.5 μg/μL Rnase A (Thermo Scientific, EN0531) at 37°C for 20 min to remove proteins and RNAs outside the EV membranes.

For miRNA assays, miRNAs and RNAs <200 nucleotides were extracted using miRNeasy Mini Kit (Qiagen, 217004) according to the manufacturer's instructions. A total amount of 20 ng small RNA was used for reverse transcription using TaqMan MicroRNA Reverse Transcription Kit (Applied Biosystems, 4366597). Then, 1 μl of cDNA was used for a real-time PCR using TaqMan Universal PCR Master Mix (Applied Biosystems, 4364340) on the ABI QuantStudio 7 Pro Real-time PCR System.

For gene expression analysis, cells or tissues were lysed with 700 μL QIAzol lysis reagent, and RNAs were separated by chloroform and precipitated by isopropanol to extract total RNAs. A total amount of 300 ng mRNA was used for reverse transcription reaction with High-Capacity cDNA Reverse Transcription Kit (Thermo Fisher, 4368814). One microliter of cDNA was used for a real-time PCR using SYBR Green Premix Ex Taq (Tli RNase H Plus) (Takara, RR420D) on the ABI QuantStudio 7 Pro Real-time PCR System.

The primers and probes used are listed in Tables S5 and S6. The data were calculated by the $2^{(-\Delta\Delta CT)}$ method.

Generation of let-7b and let-7c mutant cell lines

The let-7b and le-7c mutant cell lines were generated by the CRISPR/Cas9 system. The sequences of miRNA precursor were downloaded from miRBase v.16.0 (http://www.mirbase.org/), and sgRNAs were designed by the online tool CRISPR DESIGN (http://crispr.mit.edu/). It is not available to target the seed sequence because of the high sequence homology and short seed sequence of the let-7 family. Thus, we target the Dicer processing sites of precursor miRNAs to alter the biogenesis of let-7b and let-7c. Oligoes were purchased from Integrated DNA Technologies, and their sequences are shown in Table S6. The CRISPR/Cas9 and sgRNAs were delivered to target cells by lentiviral transduction.

RA treatment

Retinoic acid (Sigma-Aldrich, R2625) stock solution (100 mM) was prepared by dissolving 50 mg RA powder in DMSO. A single-use aliquot of RA was prepared and stored at −80°C. Primary spermatogonia were exposed to 100 nM RA 3 days after testis EV treatment. Primary

spermatogonia were collected for RT-qPCR studies at indicated time points (1–24 h).

Small RNA sequencing

RNA was extracted from EVs after treatment with proteinase K, PMSF, and RNase A using miRNeasy Mini Kit as described above. Small-RNA libraries were prepared, and the PCR products were sequenced using BGISEQ-500 technology. Small RNA sequencing was performed by BGI (Shenzhen, China). After eliminating the low-quality reads, clean reads were mapped to reference genome and to other sRNA databases using Bowtie2 and cmsearch. Classification of sRNA follows the priority rule—MiRbase> pirnabank> snoRNA(human/plant)> Rfam> other sRNA—to ensure a unique map of each entry. Novel miRNAs and piRNAs were predicted using miRDeep2 and Piano, respectively.

Mass spectrometer proteomic analysis

Testis EV proteins were extracted with RIPA buffer, and protein concentrations were detected by bicinchoninic acid (BCA) assay. Six micrograms of proteins in each sample were used to perform Bruker timsTOF Pro mass spectrometer proteomics analyses by Biosciences Central Research Facility of the Hong Kong University of Science and Technology. GO enrichment and KEGG pathway enrichment were analyzed.

Single-cell RNA sequencing

Cell clumps were digested into single cells and resuspended in the 0.04% BSA in PBS. The cell concentration should be 1,000 cells/μL and with more than 85% viability, detected by Countess II Automated Cell Counter. Then, the single-cell library was prepared according to the 10× Genomics Single Cell protocol.

The input dataset was aligned with the mouse genome (mm10, GENCODE vM23/Ensembl 98) as appropriate and used the Cell Ranger v.7.0.1 Single-Cell Software Suite from 10× Genomics to estimate partitions containing cells and their unique molecular identifiers (UMIs). Cell filtering, data normalization, and unsupervised clustering were carried out using the Seurat R package. Ultimately, we identified 23,904 genes and detected 29,853 cells across four samples. We performed principal-component analysis (PCA) on the corrected expression matrix, focusing on highly variable genes (HVGs) identified by the “FindVariableFeatures” function. Afterward, we classify different cell types with the “FindClusters” function.

To comprehensively annotate the specific types of cell clusters, we examined the expression of the following marker genes and performed hierarchical clustering analysis using the R package pheatmap: *Zbtb16*, *Lhx1*, *Bcl6b*, *Etv5*, and *Ret* for SCC cells; *Rara*, *Upp1*, and *Rarg* for progenitor cells; and *Col1a1*, *Col4a1*, *Aifm2*, *Epcam*, *Pdgfra*, *Pdpn*, *Ly6c1*, *Ly6c2*, and *Cd24a* for fibroblasts. In addition, we investigated the lineage relationship among three myofibroblast subtypes using Monocle2.

To identify differentially expressed genes between vehicle samples and PND7 testis EV samples for each cell subtype, we used the “FindMarkers” function with default parameters. A gene was considered differentially expressed if it had an adjusted p value <0.05 and an absolute log2 (fold change) > 1. We used the R package clusterProfiler to conduct KEGG pathway enrichment analysis on the significantly differentially expressed genes.

Statistical analysis

All experiments were repeated independently at least three times ($n = 3$ biological replicates). For RT-qPCR experiments, each biological replicate consisted of $n = 3$ technical replicate wells per condition. For small RNA sequencing analysis, due to the limited yield of the EV-treated primary spermatogonia and PND7 testis EVs, two sets of independent cell cultures per treatment group were used. Statistical analyses were performed using GraphPad Prism 9.0 software. Data are presented as mean ± SD by t test for two groups and one-way ANOVA for three groups of comparation; $n = 3$ independent experiments. Results were presented by mean ± SD, *$p < 0.05$, **$p < 0.01$, ***$p < 0.001$, and ****$p < 0.0001$, by Student’s t test for two groups and one-way ANOVA for three or more groups of samples, and two-way ANOVA was used for group analysis. Fisher’s least significant difference (LSD) test was used for comparison with control group, and Tukey’s honest significant difference (HSD) test for all pairwise comparison. A p value of <0.05 was considered significant.

RESOURCE AVAILABILITY

Lead contact

Further information and requests for resources and reagents should be directed to and will be fulfilled by the lead contact, Ellis Kin Lam Fok (ellisfok@cuhk.edu.hk).

Materials availability

All unique/stable reagents generated in this study are available from the lead contact with a completed materials transfer agreement.

Data and code availability

- Proteomics data have been deposited at iProX partner repository with the dataset identifier PXD068300. Single-cell RNA-seq and small RNA-seq data have been deposited at GEO accession numbers GSE308837 and GSE308840.
- Any additional information required to reanalyze the data reported in this paper is available from the lead contact upon request.

ACKNOWLEDGMENTS

We are grateful to the members of the Xie laboratory for discussing the progress of the project and providing technical support. We would like to thank the core facilities of the School of Biomedical Sciences, Chinese University of Hong Kong for the help in experiments. This work was supported in part by grants from the Research Grant Council of Hong Kong (T13N-62S), the Direct Grant of CUHK, and the Lo Kwee Seong Start-Up Fund to E.K.L.F.

AUTHOR CONTRIBUTIONS

T.Z. and E.K.L.F. conceived and designed the study. T.Z. performed the experiments and analyzed the data. K.H.K.C., S.Y.C., M.Z., X.L., H.C., and T.X. provided methodological guidance and analyzed the data. H.C., T.X., and E.K.L.F. provided funding support. E.K.L.F. and T.Z. wrote the manuscript with the help of all the authors. All authors read and approved the final manuscript.

DECLARATION OF INTERESTS

The authors declare no conflict of interest.

SUPPLEMENTAL INFORMATION

Supplemental information can be found online at https://doi.org/10.1016/j.stemcr.2025.102681.

Received: May 15, 2025
Revised: September 23, 2025
Accepted: September 23, 2025
Published: October 23, 2025

REFERENCES

Aranha, M.M., Santos, D.M., Solá, S., Steer, C.J., and Rodrigues, C.M.P. (2011). miR-34a regulates mouse neural stem cell differentiation. PLoS One *6*, e21396. https://doi.org/10.1371/journal.pone.0021396.

Bitarte, N., Bandres, E., Boni, V., Zarate, R., Rodriguez, J., Gonzalez-Huarriz, M., Lopez, I., Javier Sola, J., Alonso, M.M., Fortes, P., and Garcia-Foncillas, J. (2011). MicroRNA-451 is involved in the self-renewal, tumorigenicity, and chemoresistance of colorectal cancer stem cells. Stem Cell. *29*, 1661–1671. https://doi.org/10.1002/stem.741.

Büssing, I., Slack, F.J., and Grosshans, H. (2008). let-7 microRNAs in development, stem cells and cancer. Trends Mol. Med. *14*, 400–409. https://doi.org/10.1016/j.molmed.2008.07.001.

Choy, K.H.K., Chan, S.Y., Lam, W., Jin, J., Zheng, T., Law, T.Y.S., Yu, S.S., Wang, W., Li, L., Xie, G., et al. (2022). The repertoire of testicular extracellular vesicle cargoes and their involvement in inter-compartmental communication associated with spermatogenesis. BMC Biol. *20*, 78. https://doi.org/10.1186/s12915-022-01268-5.

Deng, Z., Du, W.W., Fang, L., Shan, S.W., Qian, J., Lin, J., Qian, W., Ma, J., Rutnam, Z.J., and Yang, B.B. (2013). The intermediate filament vimentin mediates microRNA miR-378 function in cellular self-renewal by regulating the expression of the Sox2 transcription factor. J. Biol. Chem. *288*, 319–331. https://doi.org/10.1074/jbc.M112.418830.

Fok, K.L., Bose, R., Sheng, K., Chang, C.-W., Katz-Egorov, M., Culty, M., Su, S., Yang, M., Ruan, Y.C., Chan, H.C., et al. (2017). Huwe1 regulates the establishment and maintenance of spermatogonia by suppressing DNA damage response. Endocrinology *158*, 4000–4016. https://doi.org/10.1210/en.2017-00396.

Gely-Pernot, A., Raverdeau, M., Teletin, M., Vernet, N., Féret, B., Klopfenstein, M., Dennefeld, C., Davidson, I., Benoit, G., Mark, M., and Ghyselinck, N.B. (2015). Retinoic Acid Receptors Control Spermatogonia Cell-Fate and Induce Expression of the SALL4A Transcription Factor. PLoS Genet. *11*, e1005501. https://doi.org/10.1371/journal.pgen.1005501.

Ishii, K., Kanatsu-Shinohara, M., Toyokuni, S., and Shinohara, T. (2012). FGF2 mediates mouse spermatogonial stem cell self-renewal via upregulation of Etv5 and Bcl6b through MAP2K1 activation. Development *139*, 1734–1743. https://doi.org/10.1242/dev.076539.

Kanatsu-Shinohara, M., Ogonuki, N., Inoue, K., Miki, H., Ogura, A., Toyokuni, S., and Shinohara, T. (2003). Long-term proliferation in culture and germline transmission of mouse male germline stem cells. Biol. Reprod. *69*, 612–616. https://doi.org/10.1095/biolreprod.103.017012.

Kitadate, Y., Jörg, D.J., Tokue, M., Maruyama, A., Ichikawa, R., Tsuchiya, S., Segi-Nishida, E., Nakagawa, T., Uchida, A., Kimura-Yoshida, C., et al. (2019). Competition for mitogens regulates spermatogenic stem cell homeostasis in an open niche. Cell Stem Cell *24*, 79–92.e6. https://doi.org/10.1016/j.stem.2018.11.013.

Kubota, H., Avarbock, M.R., and Brinster, R.L. (2004). Growth factors essential for self-renewal and expansion of mouse spermatogonial stem cells. Proc. Natl. Acad. Sci. USA *101*, 16489–16494. https://doi.org/10.1073/pnas.0407063101.

Lin, Y., Fang, Q., He, Y., Gong, X., Wang, Y., Liang, A., Wang, G., Gong, S., Wu, J., and Sun, F. (2020). Mouse thy1-positive spermatogonia suppress the proliferation of spermatogonial stem cells by Extracellular vesicles *in vitro*. Preprint at bioRxiv. https://doi.org/10.1101/2020.06.15.153668.

Li, Q., Li, H., Liang, J., Mei, J., Cao, Z., Zhang, L., Luo, J., Tang, Y., Huang, R., Xia, H., et al. (2021). Sertoli cell-derived exosomal MicroRNA-486-5p regulates differentiation of spermatogonial stem cell through PTEN in mice. J. Cell Mol. Med. *25*, 3950–3962. https://doi.org/10.1111/jcmm.16347.

Manku, G., and Culty, M. (2015). Mammalian gonocyte and spermatogonia differentiation: recent advances and remaining challenges. Reproduction *149*, R139–R157. https://doi.org/10.1530/REP-14-0431.

Nagano, M., Ryu, B.-Y., Brinster, C.J., Avarbock, M.R., and Brinster, R.L. (2003). Maintenance of mouse male germ line stem cells *in vitro*. Biol. Reprod. *68*, 2207–2214. https://doi.org/10.1095/biolreprod.102.014050.

Oatley, J.M., and Brinster, R.L. (2012). The germline stem cell niche unit in mammalian testes. Physiol. Rev. *92*, 577–595. https://doi.org/10.1152/physrev.00025.2011.

Oatley, J.M., Oatley, M.J., Avarbock, M.R., Tobias, J.W., and Brinster, R.L. (2009). Colony stimulating factor 1 is an extrinsic stimulator of mouse spermatogonial stem cell self-renewal. Development *136*, 1191–1199. https://doi.org/10.1242/dev.032243.

Peng, F., Li, T.-T., Wang, K.-L., Xiao, G.-Q., Wang, J.-H., Zhao, H.-D., Kang, Z.-J., Fan, W.-J., Zhu, L.-L., Li, M., et al. (2017). H19/let-7/LIN28 reciprocal negative regulatory circuit promotes breast cancer stem cell maintenance. Cell Death Dis. *8*, e2569. https://doi.org/10.1038/cddis.2016.438.

Salek, F., Baharara, J., Shahrokhabadi, K.N., and Amini, E. (2021). The guardians of germ cells; Sertoli-derived exosomes against electromagnetic field-induced oxidative stress in mouse spermatogonial stem cells. Theriogenology *173*, 112–122. https://doi.org/10.1016/j.theriogenology.2021.08.001.

Vernet, N., Dennefeld, C., Rochette-Egly, C., Oulad-Abdelghani, M., Chambon, P., Ghyselinck, N.B., and Mark, M. (2006). Retinoic acid metabolism and signaling pathways in the adult and developing mouse testis. Endocrinology *147*, 96–110. https://doi.org/10.1210/en.2005-0953.

Wang, B., Zhai, C., Li, Y., Ma, B., Li, Z., and Wang, J. (2023). Sertoli Cells-Derived Exosomal miR-30a-5p Regulates Ubiquitin E3 Ligase Zeb2 to Affect the Spermatogonial Stem Cells Proliferation and Differentiation. Reprod. Toxicol. *117*, 108340. https://doi.org/10.1016/j.reprotox.2023.108340.

Wang, S., Wang, X., Wu, Y., and Han, C. (2015). IGF-1R signaling is essential for the proliferation of cultured mouse spermatogonial stem cells by promoting the G2/M progression of the cell cycle. Stem Cells Dev. *24*, 471–483. https://doi.org/10.1089/scd.2014.0376.

Wei, M., Gao, X., Liu, L., Li, Z., Wan, Z., Dong, Y., Chen, X., Niu, Y., Zhang, J., and Yang, G. (2020). Visceral Adipose Tissue Derived Exosomes Exacerbate Colitis Severity via Pro-inflammatory MiRNAs in High Fat Diet Fed Mice. ACS Nano *14*, 5099–5110. https://doi.org/10.1021/acsnano.0c01860.

Welsh, J.A., Goberdhan, D.C.I., O'Driscoll, L., Buzas, E.I., Blenkiron, C., Bussolati, B., Cai, H., Di Vizio, D., Driedonks, T.A.P., Erdbrügger, U., et al. (2024). Minimal information for studies of extracellular vesicles (MISEV2023): From basic to advanced approaches. J. Extracell. Vesicles *13*, e12404. https://doi.org/10.1002/jev2.12404.

Yang, Q.-E., Kim, D., Kaucher, A., Oatley, M.J., and Oatley, J.M. (2013). CXCL12-CXCR4 signaling is required for the maintenance of mouse spermatogonial stem cells. J. Cell Sci. *126*, 1009–1020. https://doi.org/10.1242/jcs.119826.

Yang, R., Huang, H., Cui, S., Zhou, Y., Zhang, T., and Zhou, Y. (2020). IFN-γ promoted exosomes from mesenchymal stem cells to attenuate colitis via miR-125a and miR-125b. Cell Death Dis. *11*, 603. https://doi.org/10.1038/s41419-020-02788-0.

Yeh, J.R., Zhang, X., and Nagano, M.C. (2007). Establishment of a short-term *in vitro* assay for mouse spermatogonial stem cells. Biol. Reprod. *77*, 897–904. https://doi.org/10.1095/biolreprod.107.063057.

Yoshida, S., Takakura, A., Ohbo, K., Abe, K., Wakabayashi, J., Yamamoto, M., Suda, T., and Nabeshima, Y.-I. (2004). Neurogenin3 delineates the earliest stages of spermatogenesis in the mouse testis. Dev. Biol. *269*, 447–458. https://doi.org/10.1016/j.ydbio.2004.01.036.

Yu, Y., Nangia-Makker, P., Farhana, L., G Rajendra, S., Levi, E., and Majumdar, A.P.N. (2015). miR-21 and miR-145 cooperation in regulation of colon cancer stem cells. Mol. Cancer *14*, 98. https://doi.org/10.1186/s12943-015-0372-7.

Yun, D.-M., Gao, S., Lin, Y., Wu, X.-L., Liang, A.-J., and Sun, F. (2019). Extracellular vesicles in mouse testes elevate the level of serum testosterone. Reprod. Dev. Med. *3*, 199–204. https://doi.org/10.4103/2096-2924.274549.

Stem Cell Reports, Volume *20*

Supplemental Information

Extracellular-vesicle-mediated transfer of let-7b/7c promotes the proliferation of transition-state spermatogonia in neonatal mouse testis

Tingting Zheng, Kathleen Hoi Kei Choy, Sze Yan Chan, Min Zheng, Xiaotong Luo, Hao Chen, Ting Xie, and Ellis Kin Lam Fok

Supplementary information

Supplementary Figures

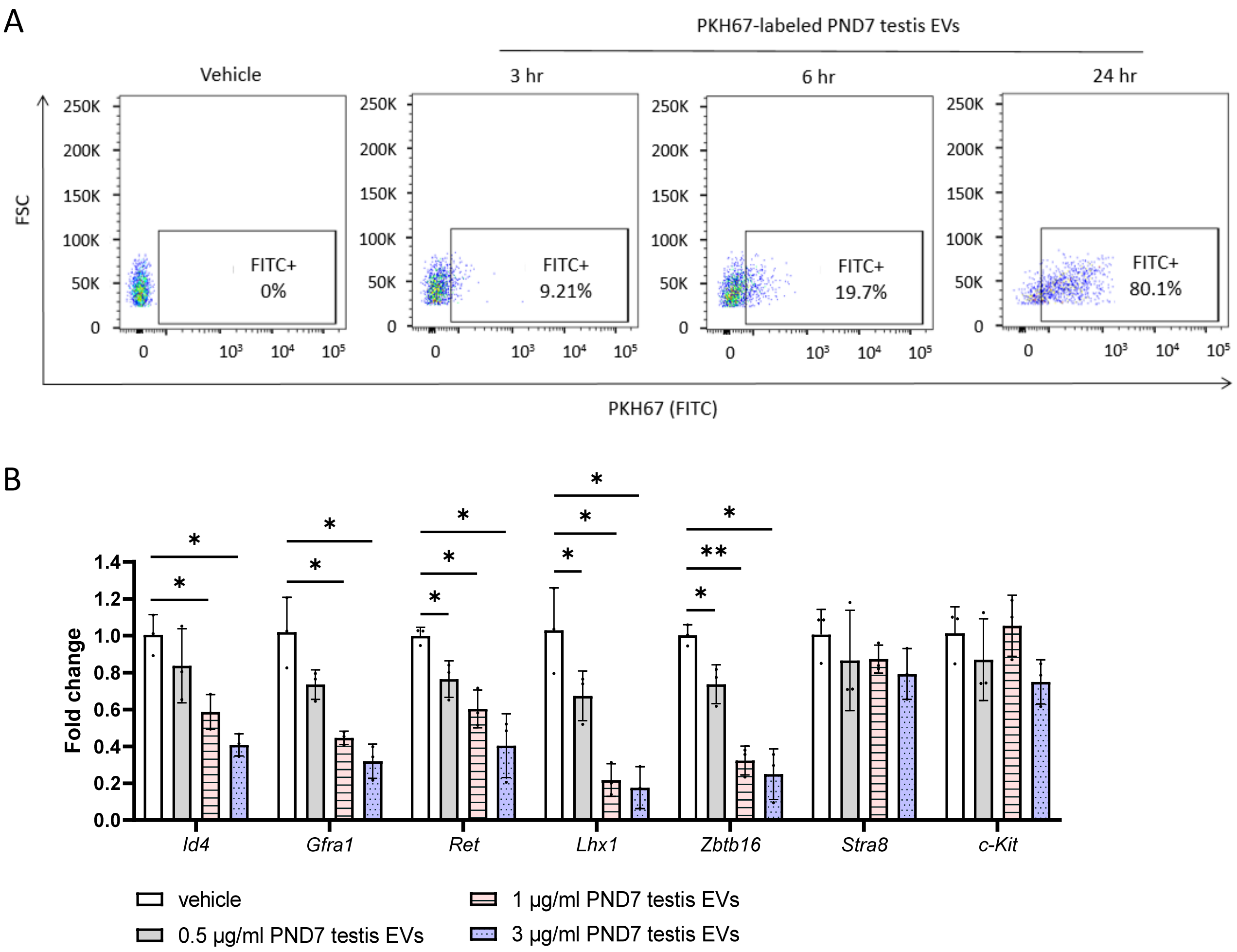

Figure S1. Prim ary spermatogonia efficiently take up PND7 testis EVs, and their expression of undifferentiated spermatogonial markers are reduced in a dose-dependent manner, related to Figure 2.

(A) Representative flow cytometry analysis of primary spermatogonia 3 h, 6 h and 24 h after co-culturing with PKH67-labeled testis EVs. Cultured spermatogonia take up PKH67-labeled testis EVs in a time-dependent manner with more than 80% of spermatogonia showed fluorescent signals after 24 h incubation.

(B) Realtime PCR results showing the expression levels of undifferentiated markers *Id4*, *Gfra1*, *Ret*, *Zbtb16* and *Lhx1* and differentiation markers *Stra8* and *c-Kit* in primary spermatogonia after exposure to indicated dose of PND7 testis EVs. n=3 independent experiments.

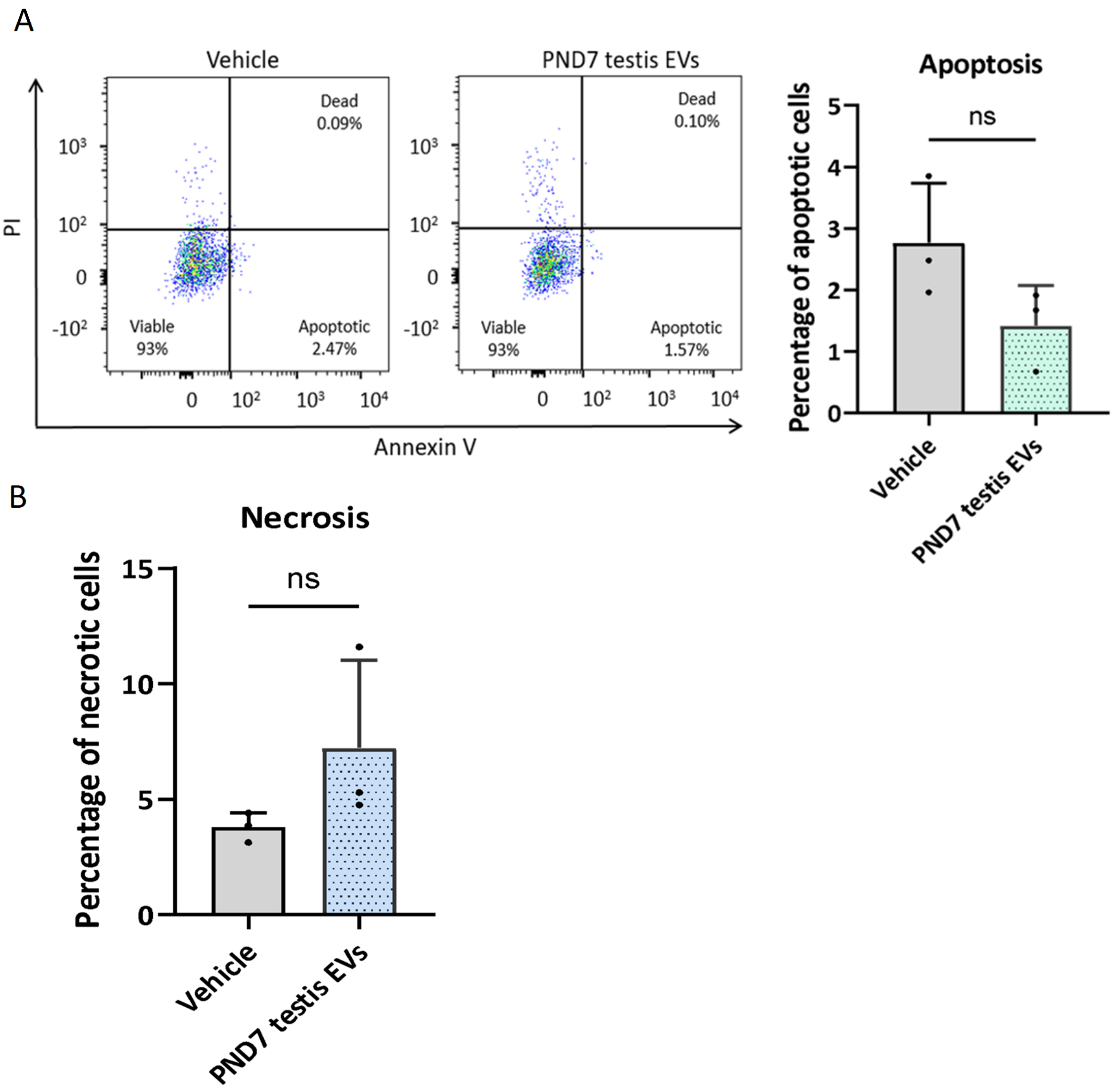

Figure S2. EVs from neonatal testis do not induce cell death in primary spermatogonial culture, related to Figure 1.

(A-B) Representative flow cytometry analysis of Annexin V-stained primary spermatogonia exposed to PND7 testicular EVs (1 µg/ml, n=3 independent experiments). Annexin V-/PI- is viable cells, Annexin V+/PI- represents apoptotic cells, Annexin V+/PI+ indicates dead cells, and Annexin V-/PI+ represents necrotic cells.

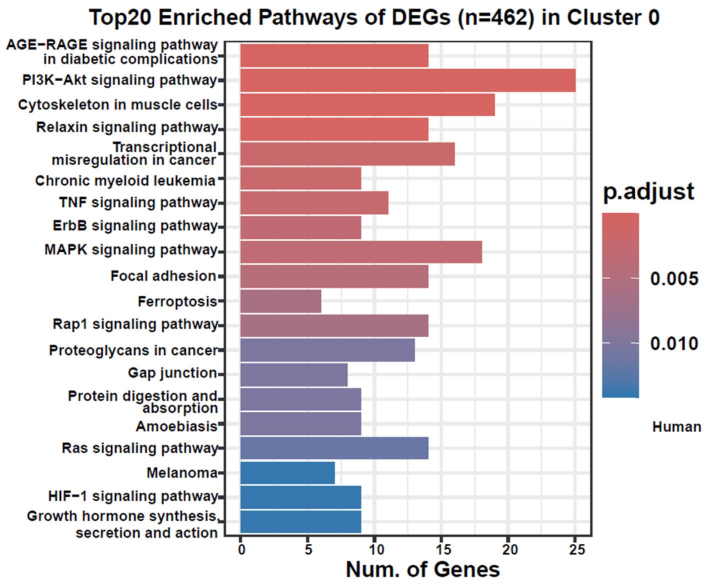
Top20 Enriched Pathways of DEGs (n=462) in Cluster 0
AGE−RAGE signaling pathway in diabetic complications
PI3K−Akt signaling pathway
Cytoskeleton in muscle cells
Relaxin signaling pathway
Transcriptional misregulation in cancer
Chronic myeloid leukemia
TNF signaling pathway
ErbB signaling pathway
MAPK signaling pathway
Focal adhesion
Ferroptosis
Rap1 signaling pathway
Proteoglycans in cancer
Gap junction
Protein digestion and absorption
Amoebiasis
Ras signaling pathway
Melanoma
HIF−1 signaling pathway
Growth hormone synthesis, secretion and action
0
5
10
15
20
25
Num. of Genes
p.adjust
0.005
0.010

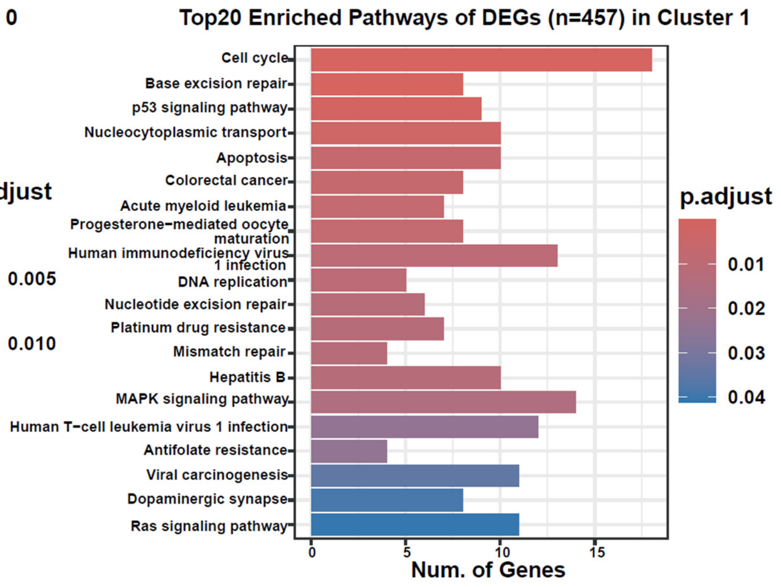
Top20 Enriched Pathways of DEGs (n=457) in Cluster 1
Cell cycle
Base excision repair
p53 signaling pathway
Nucleocytoplasmic transport
Apoptosis
Colorectal cancer
Acute myeloid leukemia
Progesterone−mediated oocyte maturation
Human immunodeficiency virus 1 infection
DNA replication
Nucleotide excision repair
Platinum drug resistance
Mismatch repair
Hepatitis B
MAPK signaling pathway
Human T−cell leukemia virus 1 infection
Antifolate resistance
Viral carcinogenesis
Dopaminergic synapse
Ras signaling pathway
0
5
10
15
Num. of Genes
p.adjust
0.01
0.02
0.03
0.04

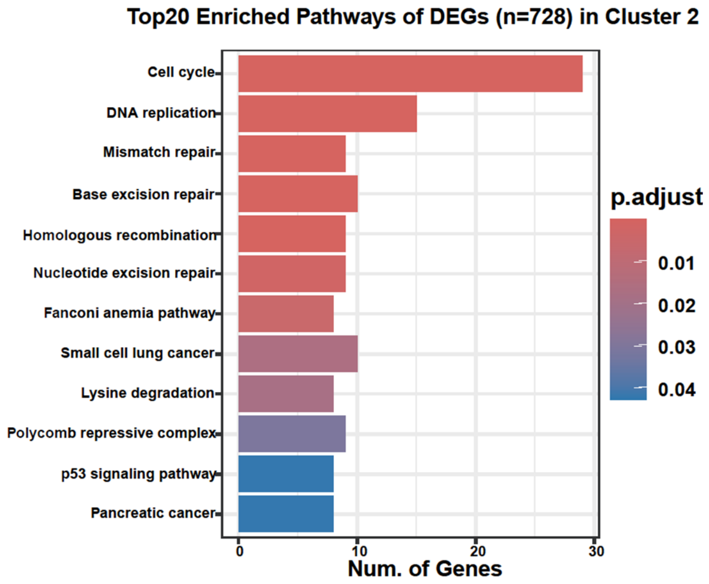
Top20 Enriched Pathways of DEGs (n=728) in Cluster 2
Cell cycle
DNA replication
Mismatch repair
Base excision repair
Homologous recombination
Nucleotide excision repair
Fanconi anemia pathway
Small cell lung cancer
Lysine degradation
Polycomb repressive complex
p53 signaling pathway
Pancreatic cancer
0
10
20
30
Num. of Genes
p.adjust
0.01
0.02
0.03
0.04

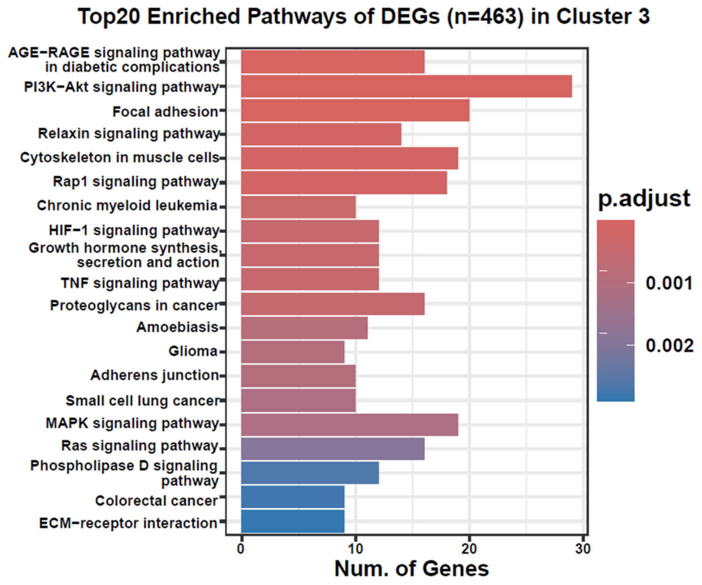
Top20 Enriched Pathways of DEGs (n=463) in Cluster 3
AGE−RAGE signaling pathway in diabetic complications
PI3K−Akt signaling pathway
Focal adhesion
Relaxin signaling pathway
Cytoskeleton in muscle cells
Rap1 signaling pathway
Chronic myeloid leukemia
HIF−1 signaling pathway
Growth hormone synthesis, secretion and action
TNF signaling pathway
Proteoglycans in cancer
Amoebiasis
Glioma
Adherens junction
Small cell lung cancer
MAPK signaling pathway
Ras signaling pathway
Phospholipase D signaling pathway
Colorectal cancer
ECM−receptor interaction
0
10
20
30
Num. of Genes
p.adjust
0.001
0.002

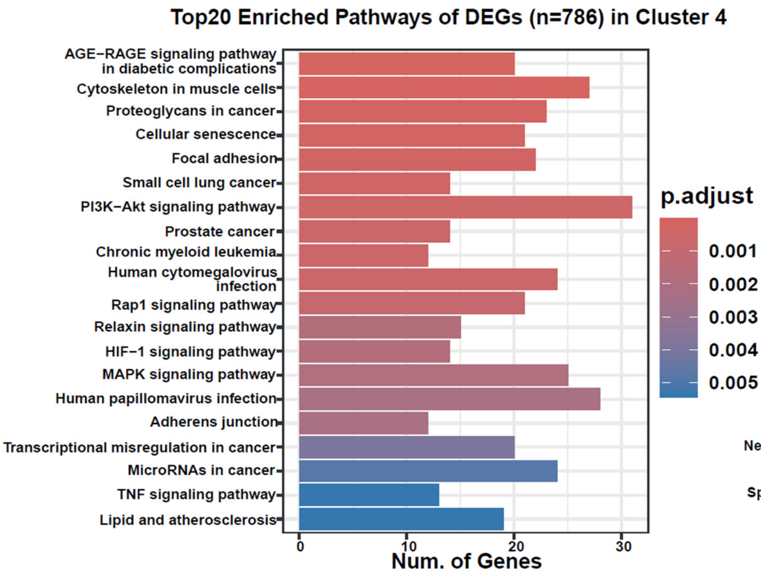
Top20 Enriched Pathways of DEGs (n=786) in Cluster 4
AGE−RAGE signaling pathway in diabetic complications
Cytoskeleton in muscle cells
Proteoglycans in cancer
Cellular senescence
Focal adhesion
Small cell lung cancer
PI3K−Akt signaling pathway
Prostate cancer
Chronic myeloid leukemia
Human cytomegalovirus infection
Rap1 signaling pathway
Relaxin signaling pathway
HIF−1 signaling pathway
MAPK signaling pathway
Human papillomavirus infection
Adherens junction
Transcriptional misregulation in cancer
MicroRNAs in cancer
TNF signaling pathway
Lipid and atherosclerosis
0
10
20
30
Num. of Genes
p.adjust
0.001
0.002
0.003
0.004
0.005

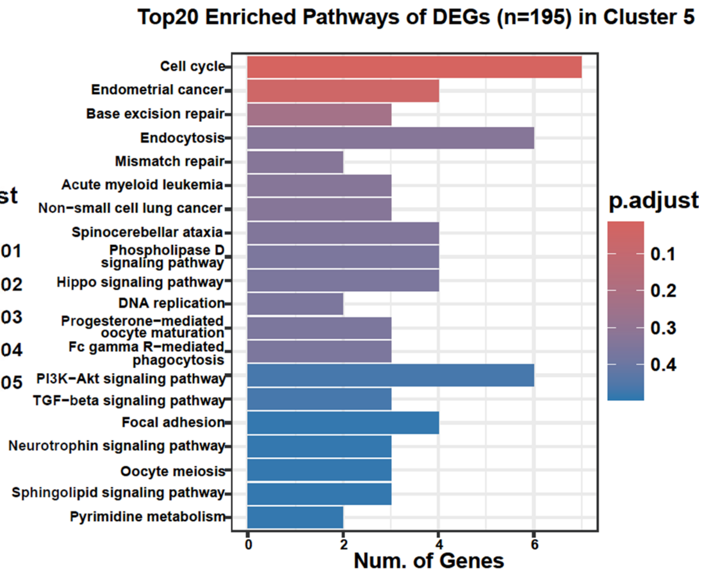
Top20 Enriched Pathways of DEGs (n=195) in Cluster 5
Cell cycle
Endometrial cancer
Base excision repair
Endocytosis
Mismatch repair
Acute myeloid leukemia
Non−small cell lung cancer
Spinocerebellar ataxia
Phospholipase D signaling pathway
Hippo signaling pathway
DNA replication
Progesterone−mediated oocyte maturation
Fc gamma R−mediated phagocytosis
PI3K−Akt signaling pathway
TGF−beta signaling pathway
Focal adhesion
Neurotrophin signaling pathway
Oocyte meiosis
Sphingolipid signaling pathway
Pyrimidine metabolism
0
2
4
6
Num. of Genes
p.adjust
0.1
0.2
0.3
0.4

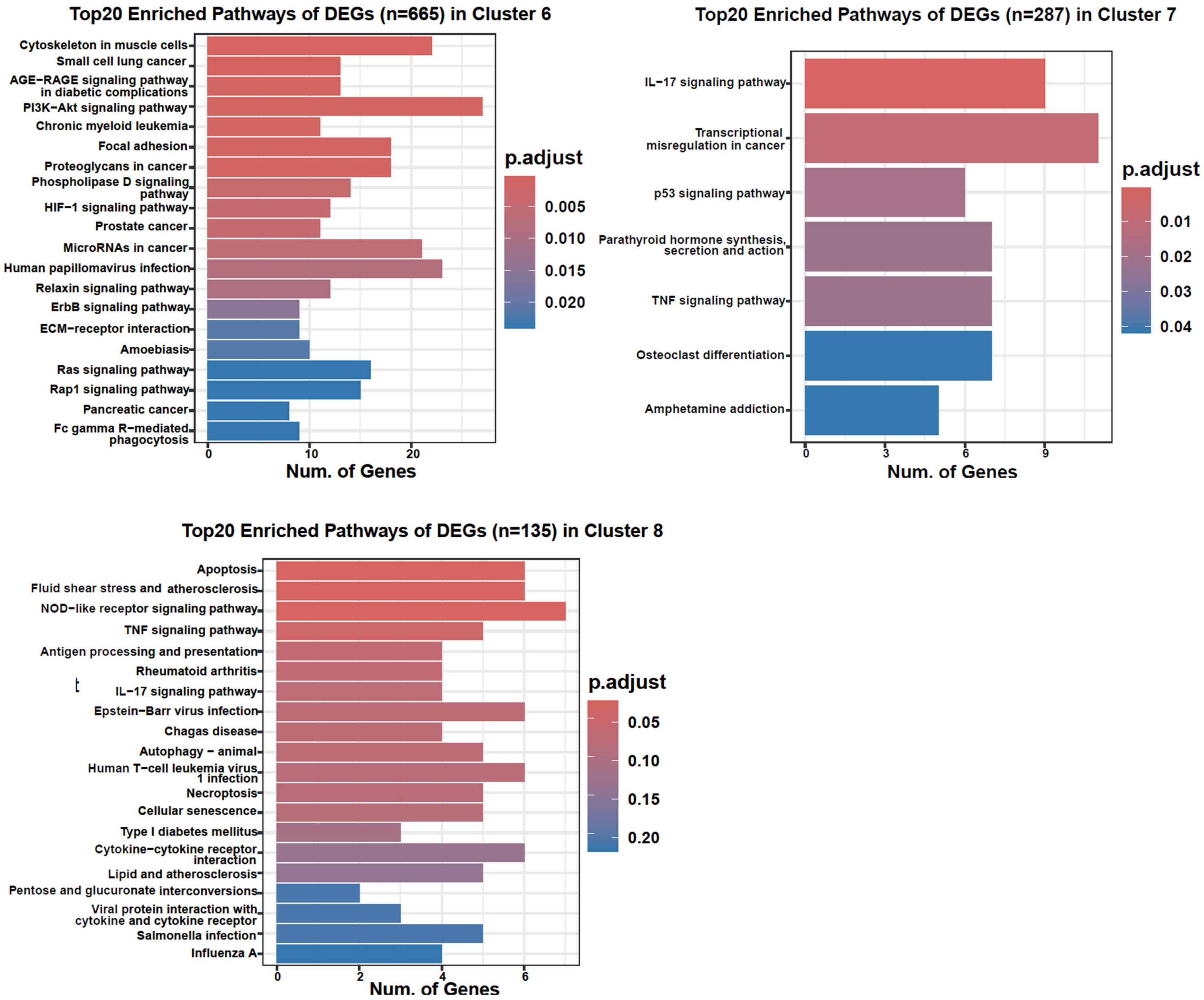

Figure S3. PND7 testicular EVs alter signaling pathways within the same spermatogonial cell cluster, related to Figure 3.

KEGG pathway enrichment analysis of the significantly differentially expressed genes between control and PND7 testicular EV-treated group in each spermatogonial subpopulation clustered by single-cell RNA sequencing, n=2 independent experiments.

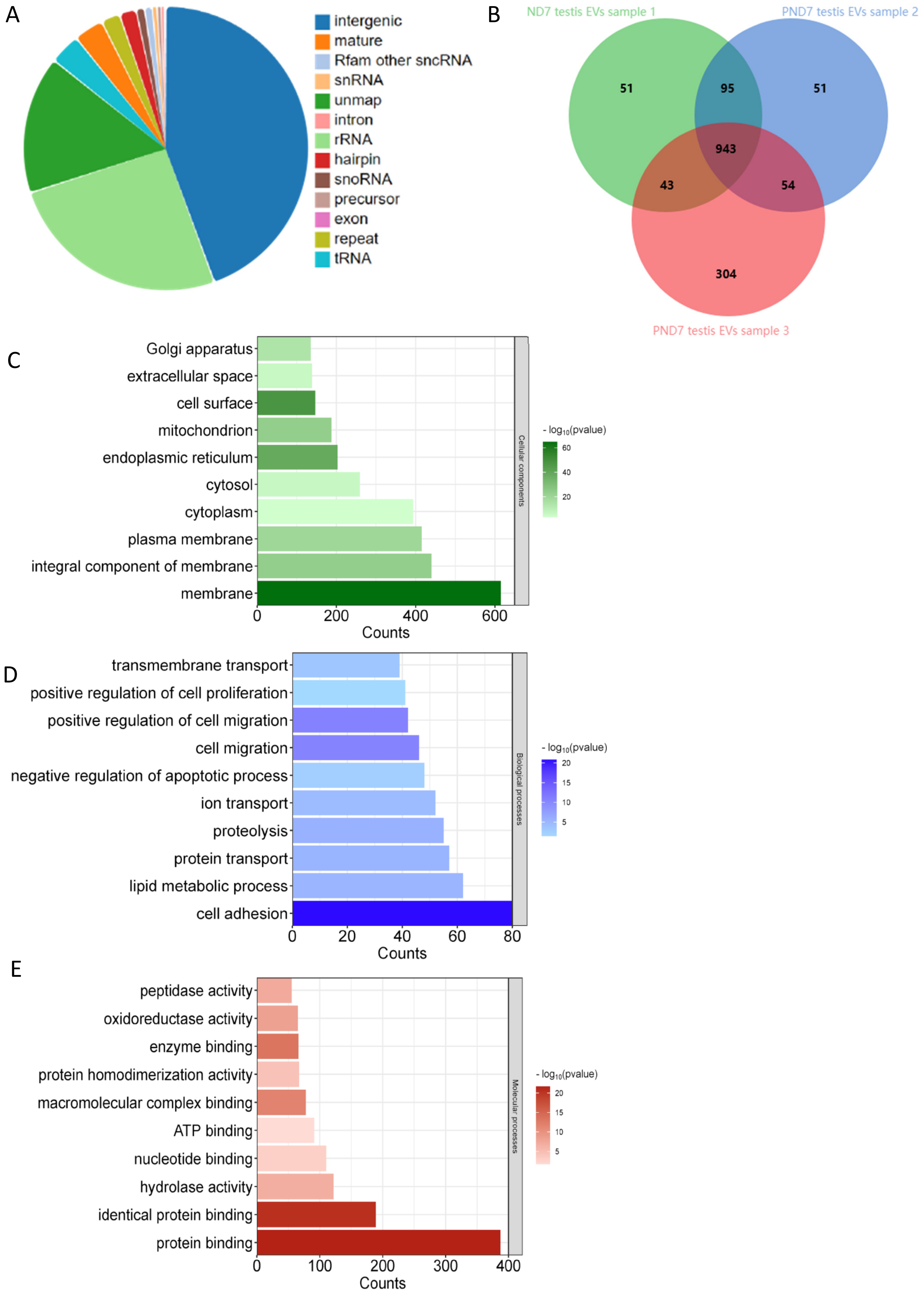
A
intergenic
mature
Rfam other sncRNA
snRNA
unmap
intron
rRNA
hairpin
snoRNA
precursor
exon
repeat
tRNA
B
ND7 testis EVs sample 1
PND7 testis EVs sample 2
PND7 testis EVs sample 3
51
95
51
943
43
54
304
C
Golgi apparatus
extracellular space
cell surface
mitochondrion
endoplasmic reticulum
cytosol
cytoplasm
plasma membrane
integral component of membrane
membrane
0
200
400
600
Counts
Cellular components
- log10(pvalue)
60
40
20
D
transmembrane transport
positive regulation of cell proliferation
positive regulation of cell migration
cell migration
negative regulation of apoptotic process
ion transport
proteolysis
protein transport
lipid metabolic process
cell adhesion
0
20
40
60
80
Counts
Biological processes
- log10(pvalue)
20
15
10
5
E
peptidase activity
oxidoreductase activity
enzyme binding
protein homodimerization activity
macromolecular complex binding
ATP binding
nucleotide binding
hydrolase activity
identical protein binding
protein binding
0
100
200
300
400
Counts
Molecular processes
- log10(pvalue)
20
15
10
5

Figure S4. The repertoire of small RNA and protein cargoes in the EVs isolated from neonatal testis.

(A) Pie chart showing the distribution of different species of small RNAs identified in PND7 testicular EVs by RNA sequencing (n=3 samples, each sample represents the pooled testicular EVs isolated from 10 mice).

(B) Venn analysis showed 943 identified proteins in PND7 testicular EVs (n=3 samples, each sample represents the pooled testicular EVs isolated from 3 mice).

(C-E) Gene Ontology analysis on (C) cellular component, (D) biological process and (E) molecular function of proteins identified in PND7 testicular EVs.

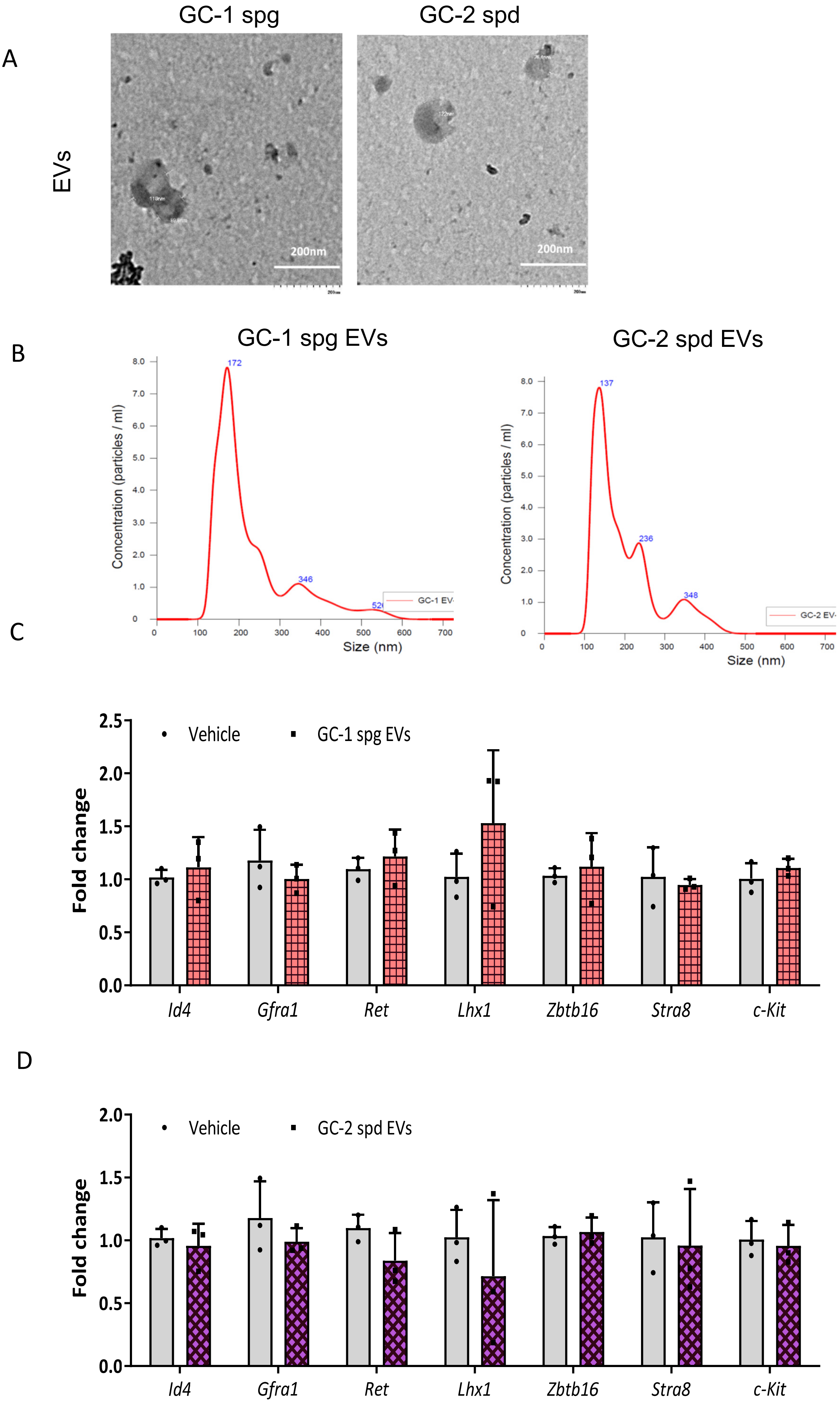
A
GC-1 spg
GC-2 spd
EVs
200nm
200nm
B
GC-1 spg EVs
GC-2 spd EVs
Concentration (particles / ml)
Size (nm)
172
346
137
236
348
C
Fold change
Vehicle
GC-1 spg EVs
Id4
Gfra1
Ret
Lhx1
Zbtb16
Stra8
c-Kit
D
Fold change
Vehicle
GC-2 spd EVs
Id4
Gfra1
Ret
Lhx1
Zbtb16
Stra8
c-Kit

Figure S5. EVs isolated from differentiated germ cell lines pose negligible effect on the expression of spermatogonia markers in primary spermatogonial culture, related to Figure 4.

(A) Representative transmission electron microscopy image of EVs isolated from GC1-spg and GC2-spd cell lines by ultracentrifugation. Scale bar: 200 nm.

(B) Size distribution of cell line-derived EVs isolated as in (A) determined by nanoparticle tracking analysis.

(C-D) Real-time PCR results showing the expression of SSCs marker *Id4*, As spermatogonia marker *Gfra1* and pan-undifferentiated spermatogonia markers *Zbtb16*, *Ret* and *Lhx1*, and spermatogonial differentiation markers *Stra8* and *c-Kit* in primary spermatogonia after treatment with EVs isolated from GC1-spg (C) and GC2-spd (D) cell lines (1 μg/ml). Data is presented as mean ± S.D. *P<0.05, **P<0.01, ***P<0.001, by t-test; n=3 independent experiments.

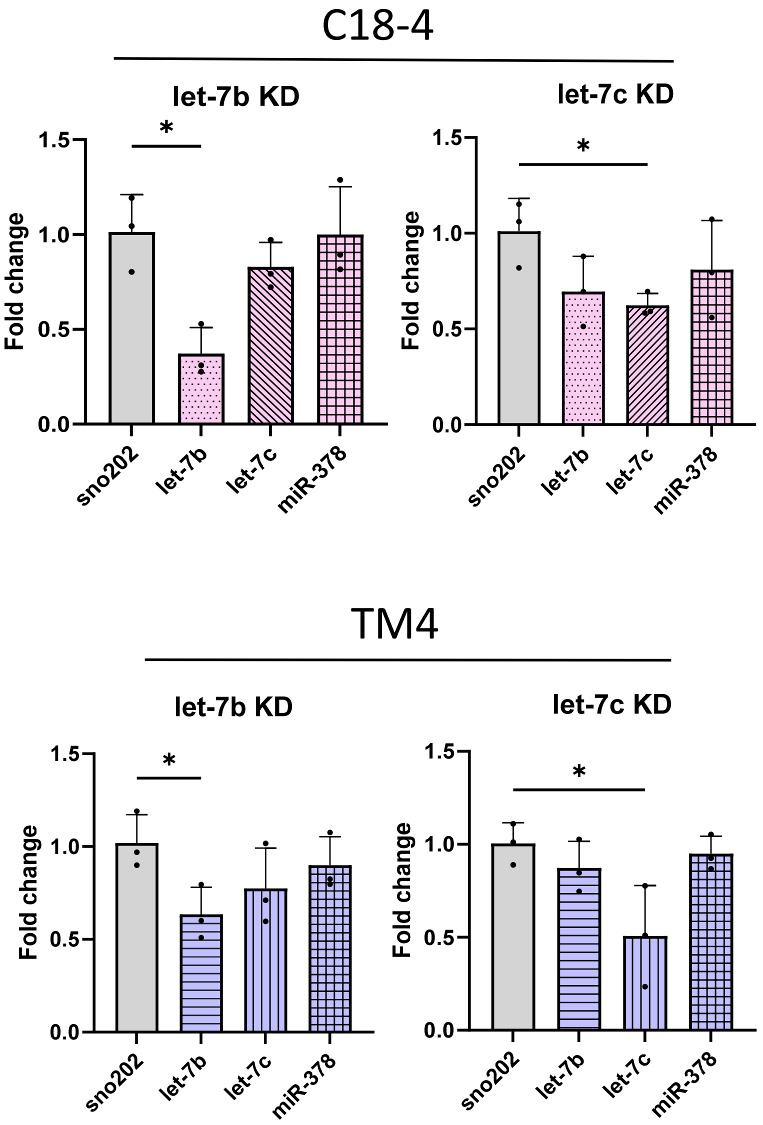

Figure S6. Knockdown of let-7b and 7c in C18-4 and TM4 cell lines, related to Figure 6.

Real-time PCR results showing the levels of let-7b, let-7c and miR378 in wild type (WT) and mutants (let-7b KD and let-7c KD) C18-4 or TM4 cell lines. Data is presented as mean ± S.D. *P<0.05, by t-test; n=3 independent experiments.

Supplemental tables

Table S1. List of top 50 most abundant miRNAs in PND7 testicular EVs and their functions on stem cells.

Table S2. List of proteins identified in PND7 testicular EVs.

Table S3. Protein levels of exosome protein markers in PND7 testicular EVs.

Table S4. Functions of genes encoding proteins identified in PND7 testicular EVs on spermatogenesis or male fertility.

Accession	Gene name	Description	Average		Functions
			#Unique	-10lgP	
P16406|AMPE_MOUSE	Enpep	Glutamyl aminopeptidase	31	389.96	male fertility
P43406|ITAV_MOUSE	Itgav	Integrin alpha-V	27	354.19	Expressed on the surface o f porcine SSCs
Q60675|LAMA2_MOUSE	Lama2	Laminin subunit alpha-2	24	309.88	Sertoli cell functions
Q61739|ITA6_MOUSE	Itga6	Integrin alpha-6	21.67	360.33	SSC marker
Q8BTM8|FLNA_MOUSE	Flna	Filamin-A	21.33	297.93	SSC proterty
P20029|BIP_MOUSE	Hspa5	Endoplasmic reticulum ch aperone BiP	19.67	339.41	male fertility
P02468|LAMC1_MOUSE	Lamc1	Laminin subunit gamma-1	19	321.01	stem cell maintenance
Q3U1J4|DDB1_MOUSE	Ddb1	DNA damage-binding protein 1	17.33	250	SSC maintenance<br>Sertoli cell proliferation
Q9WV91|FPRP_MOUSE	Ptgfrn	Prostaglandin F2 receptor negative regulator	17	302.75	male fertility
Q60597|ODO1_MOUSE	Ogdh	2-oxoglutarate dehydrogena se mitochondrial	15	260.69	hESC maintenance
P57780|ACTN4_MOUSE	Actn4	Alpha-actinin-4	13.67	311.71	CSC property
Q3UHK6|TEN4_MOUSE	Tenm4	Teneurin-4	13.67	252.34	CSC property
P27773|PDIA3_MOUSE	Pdia3	Protein disulfide-isomerase A3	13	273.57	male fertility
P19096|FAS_MOUSE	Fasn	Fatty acid synthase	12.67	259.96	NSPC activity
Q61738|ITA7_MOUSE	Itga7	Integrin alpha-7	12	265.42	stem cell property
Q61503|5NTD_MOUSE	Nt5e	5'-nucleotidase	11.67	274.42	spermatogenesis
P09055|ITB1_MOUSE	Itgb1	Integrin beta-1	11.67	256.26	SSC marker
P62259|1433E_MOUSE	Ywhae	14-3-3 protein epsilon	11.33	257.45	male fertility
P07356|ANXA2_MOUSE	Anxa2	Annexin A2	11.33	243.76	male fertility
P23249|MOV10_MOUSE	Mov10	Putative helicase MOV-10	11	263	male fertility
Q62469|ITA2_MOUSE	Itga2	Integrin alpha-2	10.67	255.04	stem cell property
Q8BKG3|PTK7_MOUSE	Ptk7	Inactive tyrosine-protein kinase 7	10.67	236.13	stem cell property
P47738|ALDH2_MOUSE	Aldh2	Aldehyde dehydrogenase mitochondrial	10.33	278.15	stem cell property
P42703|LIFR_MOUSE	Lifr	Leukemia inhibitory facto r receptor	10.33	233.89	male fertility
Q64521|GPDM_MOUSE	Gpd2	Glycerol-3-phosphate dehydrogenase mitochondria	10	231.68	male fertility

Note:

1. Enriched genes are reported to orchestrate SSC properties (red words) or stem cell properties (blue words).
2. CSC, cancer stem cell; hESC, human embryonic stem cell; NSPC, neural stem and progenitor cell; SSC, spermatogonial stem cell.

Table S5. List of TaqMan probes used for real time PCR analysis of candidate miRNAs, related to Figure 5.

Primer name	Mature sequence	Assay ID
hsa-miR-125b	5’-UCCCUGAGACCCUAACUUGUGA-3’	ThermoFisher, 000449
hsa-miR-21	5’-UAGCUUAUCAGACUGAUGUUGA-3’	ThermoFisher, 000397
hsa-miR-34a	UGGCAGUGUCUUAGCUGGUUGU-3’	ThermoFisher, 000426
hsa-miR-378	5’-ACUGGACUUGGAGUCAGAAGG-3’	ThermoFisher, 002243
mmu-miR-451	5’-AAACCGUUACCAUUACUGAGUU-3’	ThermoFisher, 001141
hsa-miR-652	5’-AAUGGCGCCACUAGGGUUGUG-3’	ThermoFisher, 002352
hsa-let-7a	5’-UGAGGUAGUAGGUUGUAUAGUU-3’	ThermoFisher, 000377
hsa-let-7b	5’-UGAGGUAGUAGGUUGUGUGGUU-3’	ThermoFisher, 000378
hsa-let-7c	5’-UGAGGUAGUAGGUUGUAUGGUU-3’	ThermoFisher, 000379
hsa-let-7f	5’-UGAGGUAGUAGAUUGUAUAGUU-3’	ThermoFisher, 000382
snoRNA202	5’-<br>GCTGTACTGACTTGATGAAAGTACTTT<br>TGAACCCTTTTCCATCTGATG-3’	ThermoFisher, 001232
U6 snRNA	5’- GTGCTCGCTTCGGCAGCACATATAC<br>TAAAATTGGAACGATACAGAGAAGAT<br>TAGCATGGCCCCTGCGCAAGGATGAC<br>ACGCAAATTCGTGAAGCGTTCCATATT<br>TT-3’	ThermoFisher, 001973

Table S6. List of primers and oligos used in this study, related to Figure 2and 4.

Primer name	Sequence	Tm
mId4 F	5'-TGATATGCGCACTCTAACCGT-3'	66°C
mId4 R	5'-CGATCCCTGAACATGTCCATCAG-3'	67°C
mGfra1 F	5'-TTCTTGCAGGTGTCATCCAG-3'	65 °C
mGfra1 R	5'-GATATATTCCGGGCAGTCCC-3'	65 °C
mPlzf F	5'-CGTGCGCAGCTATATTTGCA-3'	66°C
mPlzf R	5'-TGGCTCTTGAGTGTGCTCTCA-3'	68°C
mStra8 F	5'-CATCATCACTGGGTTGGTTG-3'	64°C
mStra8 R	5'-CTGCGTGTTCCACAAGTGTC-3'	67°C
m-c-kit F	5'-GGCCTCACGAGTTCTATTTACG-3'	65°C
m-c-kit R	5'-GGGGAGAGATTTCCCATCACAC-3'	67°C
m-c-Ret F	5'-CCGTAGGGCATGGACATAGA-3'	66°C
m-c-Ret R	5'-GCTGAAGCTGATTTTGCTCC-3'	64°C
mLhx F	5'-AACCAGATCGCTTGGAGAGA-3'	66°C
mLhx R	5'-TGTAAATGCAACCTGACCGA-3'	64°C
mNgn3-rt F	5'-TGGCGCCTCATCCCTTGGATG-3	73°C
mNgn3-rt R	5'-CAGTCACCCACTTCTGCTTCG-3	68°C
mLin28-rt F	5'-AAGATCCAAAGGAGACAGGTGC-3'	67°C
mLin28-rt R	5'-GGAAGTAGGCAGGCTTTCCC-3'	68°C
mRara F	5'-TGTAAGGGCTTCTTCCGACGA-3'	68°C
mRara R	5'-GCTTGGGTGCCTCTTTCTTC-3'	66°C
mRarg F	5'-TGCCTGGTTTTACAGGGCTC-3'	60°C
mRarg R	5'-TCCGAGAATGTCATAGTGTCCT-3'	56°C
mDdx4 F	5'-GCTTCATCAGATATTGGCGAGT-3'	65°C
mDdx4 R	5'-GCTTGGAAAACCCTCTGCTT-3'	66°C
GAPDH F	5'-GACCACAGTCCATGCCATCACTGC-3'	73°C
GAPDH R	5'-GCTGTTGAAGTCGCAGGAGACAAC-3'	70°C
LeGO-PCR-F1	5'-AATATGCATGCGAGAAAAGCCTTG-3'	59°C
LeGO-PCR-F2	5'-GTTTTAGAGCTAACCGGTTAGCAAGT-3'	59°C
LeGO-PCR-R2	5'-TACCACCACACTGGGATCC-3'	57°C
glet7b-48 oligo1	5'-cgagaaaagccttgtttgGGTTGTATAGTTATCTTCGGgttttagagctaa-3'	/
glet7b-48 oligo2	5'-ccggttagctctaaaacCCGAAGATAACTATACAACCcaaacaaggcttttctc-3'	/
glet7c-49 oligo1	5'-cgagaaaagccttgtttgAGGTTGTACAGTTAACTCCCgttttagagctaa-3'	/
glet7c-49 oligo2	5'-ccggttagctctaaaacGGGAGTTAACTGTACAACCTcaaacaaggcttttctc-3'	/

Supplemental experimental procedures

Adipose-derived EVs isolation

Adipose-derived EVs were isolated from mouse visceral adipose tissue, as previously reported (Wei et al., 2020). Tissue samples were washed in PBS, minced into ~2 mm^3 fragments, and incubated in serum-free medium at 37°C for 24 h. The resulting conditioned medium was collected, and EVs were isolated using differential ultracentrifugation, as previously described. Briefly, CCM was centrifuged at 300g 10 min to remove live cells and 3000g 20 min to remove cell debris, followed by 10,000g 30 min 4°C (Beckman Avanti J-E Centrifuge) to remove large vesicles. Lastly, the supernatant was ultracentrifuged (Beckman Optima XPN-100 Ultra-High Speed Refrigerated Centrifuge) at 100,000g 4°C for 90 min to pellet the small vesicles. The pellets were washed by PBS with ultracentrifuge (Hitachi CS150FNX Ultra-Microcentrifuge) at 100,000g 4°C for 90 min. The EV pellet was resuspended in 20 µl PBS and stored at -80℃ for further analysis.

Transmission electron microscope (TEM)

The morphology of EVs was observed using a transmission electron microscope. Briefly, EVs were resuspended in 20 µl PBS and fixed with 2% paraformaldehyde until use. 10 µl EVs were added onto the formvar grid (200 mesh) for 30–60 min, and excess fluid was removed with filter paper. EVs were fixed with 1% glutaraldehyde for 10 min, followed by negative staining with 2% uranyl acetate for 2 min, and three images were captured at different fields using a Hitachi H-7700 transmission electron microscope.

Nanoparticle tracking analysis (NTA)

The concentration of EVs was measured by the Nanosight LM14C (Malvern) instrument. Briefly, EV samples were diluted with filtered PBS and the particle concentration was adjusted to the range of ~107-109 particles/ml, which allows the viewing of approximately 20-100 particles in each field. A thirty-second video was recorded of each sample and captured at 3 different fields. The concentration of testis EVs was analyzed and calculated by the NanoSight software using the Stokes-Einstein equation.

Uptake of PKH67-labeled testis EVs

Purified EVs were labeled using the PKH67 Green Fluorescent Cell Linker Mini Kit (Sigma-Aldrich) according to the manufacturer's instructions. Briefly, 100 µl of EVs resuspended in PBS were mixed with 1 ml Diluent C and 6 µl PKH67 dye, followed by 5-min incubation. Excess dye was quenched by adding 2 mL of 0.5% BSA/PBS, and 20 ml PBS was added to prevent vesicle aggregation during ultracentrifugation. Labeled EVs were washed via ultracentrifugation and resuspended in 50 µl PBS. Primary spermatogonia were co-cultured with labeled EVs for 3, 6, and 24 h, and EV uptake was analyzed by flow cytometry.

Proliferation assay

The culture medium was removed and replaced with fresh medium containing 10 µM BrdU for 12 hours at 37ºC. After incubation, spermatogonial clumps were collected by flushing from the feeder cells as described (Fok et al., 2017). The clumps were pelleted by centrifugation at 300g for 5 min. Then, the clumps were trypsinized into a single-cell suspension (Gibco). The cells

were washed twice with PBS and then fixed by dropwisely adding 5 ml ice-cold 70% ethanol and incubated at -20°C for 2h. After fixation, the cells were permeabilized with 2 M HCl/0.5% Triton X-100 at room temperature for 30 minutes. After washing twice with PBS supplemented with 1% BSA, cells were incubated with the anti-BrdU antibody (Abcam, ab6326, 1:100) and anti-DDX4 antibody (Abcam, ab13840, 1:100) in the solution of 1% BSA in PBS (add 1 g of BSA to 100 ml of PBS) at room temperature for 1 hour. After three times washes with the solution of 1% BSA in PBS, the samples were stained with Alexa Fluor 488 anti-rat IgG (H+L) (Invitrogen, A21208,1:1000) and Alex Fluor 647 anti-rabbit IgG (H+L) (Invitrogen, A31573, 1:1000) and incubated at room temperature for 1 hr. Lastly, the cells were washed with the solution of 1% BSA in PBS three times and resuspended in the solution of 1% BSA in PBS to 106-107 cells/ml and analyzed flow cytometry (BD LSRFortessa™ Cell Analyzer). The results were analyzed by FlowJo_V10.

Single-cell RNA sequencing

Cell clumps were digested into single cells by 0.05% trypsin. Cells were washed thrice with 0.04% BSA in PBS and resuspended in the 0.04% BSA in PBS. Cell concentration and viability were determined using a Countess® II Automated Cell Counter. The cell concentration should be 1000 cells/μl and more than 85% viability. Then, the single-cell library was prepared according to the 10x Genomics® Single Cell Protocol.

The input dataset was aligned with the mouse genome (mm10, GENCODE vM23/Ensembl 98) as appropriate and used the Cell Ranger v.7.0.1 Single-Cell Software Suite from 10x Genomics to estimate partitions containing cells and their unique molecular identifiers (UMIs). The Seurat R package was used for downstream analyses. Additional quality control measures were applied based on the following criteria: 1) the number of expressed genes must be greater than 400 and less than 4,000; 2) cells should have mitochondrial RNA content below 10%. The DoubletFinder R package was employed to remove potential doublets. The filtered gene expression matrix for each sample was normalized and scaled using the "NormalizeData" and "ScaleData" functions in Seurat. Ultimately, we identified 23904 genes and detected 29853 cells across four samples. We performed principal component analysis (PCA) on the corrected expression matrix, focusing on highly variable genes (HVGs) identified by the "FindVariableFeatures" function. Afterward, we conducted PCA using the "RunPCA" function and constructed a K-nearest-neighbor graph with the "FindNeighbors" function. The most representative principal components were then used to classify different cell types with the "FindClusters" function.

To comprehensively annotate the specific types of cell clusters, we examined the expression of the following marker genes and performed hierarchical clustering analysis using the R package pheatmap: Zbtb16, Lhx1, Bcl6b, Etv5, and Ret for SCC cells; Rara, Upp1, and Rarg for progenitor cells; and Col1a1, Col4a1, Aifm2, Epcam, Pdgfra, Pdpn, Ly6c1, Ly6c2, and Cd24a for fibroblasts. In addition, we investigated the lineage relationship among three myofibroblast subtypes using Monocle2. Furthermore, we assessed the cell cycle using the CellCycleScoring function in the Seurat R package, which utilizes the cell cycle genes provided by Seurat. Based on the scores of these cell cycle genes, we classified each cell's phase as follows: if both the S

and G2M phase scores are less than 0, the cell is categorized as being in the G1 phase. If at least one score is greater than 0, the cell is assigned to the phase corresponding to the higher score.

To identify differentially expressed genes between Vehicle samples and PND7 testis EV samples for each cell subtype, we used the "FindMarkers" function (for two-condition comparisons) from the Seurat package with default parameters. A gene was considered differentially expressed if it had an adjusted P-value < 0.05 and an absolute log2 (fold change) > 1. We used the R package clusterProfiler to conduct KEGG pathway enrichment analysis on the significantly differentially expressed genes.

Small RNA sequencing

RNA was extracted from EVs after treatment with proteinase K, PMSF and RNase A using miRNeasy Mini Kit as described above. Small-RNA libraries were prepared, and the PCR products were sequenced using BGISEQ-500 technology. Small RNA sequencing was performed by BGI (Shenzhen, China). After eliminating the low-quality reads, clean reads were mapped to reference genome and to other sRNA databases using Bowtie2 and cmsearch. Classification of sRNA follows the priority rule: MiRbase> pirnabank> snoRNA(human/plant)> Rfam> other sRNA to ensure a unique map of each entry. Novel miRNAs and piRNAs were predicted using miRDeep2 and Piano, respectively.

Mass spectrometer proteomic analysis

Testis EV proteins were extracted with RIPA buffer, and protein concentrations were detected by BCA assay. 6 μg of proteins in each sample were used to perform Bruker timsTOF Pro Mass Spectrometer proteomics analyses by Biosciences Central Research Facility of the Hong Kong University of Science and Technology. Gene ontology (GO) enrichment and KEGG pathway enrichment were analyzed.

Generation of let-7b and let-7c mutant cell lines

The let-7b and le-7c mutant cell lines were generated by the CRISPR/Cas9 system. The sequences of miRNA precursor were downloaded from miRBase v16.0 (http://www.mirbase.org/) and sgRNAs were designed by the online tool CRISPR DESIGN (http://crispr.mit.edu/). Designs were selected by specificity score. Due to the sequence homology and short sequence of the let-7 family, designs that target the seed sequence were not available. Thus, designs that target the Dicer processing sites of precursor miRNAs, which alter the biogenesis of let-7b and let-7c, were used. Oligoes were purchased from Integrated DNA Technologies and their sequences are shown in Table S2. The CRISPR/Cas9 and sgRNAs were delivered to target cells by lentiviral transduction.

Supplemental references

Wei, M., Gao, X., Liu, L., Li, Z., Wan, Z., Dong, Y., Chen, X., Niu, Y., Zhang, J., and Yang, G. (2020). Visceral Adipose Tissue Derived Exosomes Exacerbate Colitis Severity via Pro-inflammatory MiRNAs in High Fat Diet Fed Mice. ACS Nano 14, 5099–5110. https://doi.org/10.1021/acsnano.0c01860.

Fok, K.L., Bose, R., Sheng, K., Chang, C.-W., Katz-Egorov, M., Culty, M., Su, S., Yang, M., Ruan, Y.C., Chan, H.C., et al. (2017). Huwe1 regulates the establishment and maintenance of spermatogonia by suppressing DNA damage response. Endocrinology 158, 4000–4016. https://doi.org/10.1210/en.2017-00396.
